# Styryl-based new organic chromophores bearing free amino and azomethine groups: synthesis, photophysical, NLO, and thermal properties

**DOI:** 10.3762/bjoc.16.189

**Published:** 2020-09-14

**Authors:** Anka Utama Putra, Deniz Çakmaz, Nurgül Seferoğlu, Alberto Barsella, Zeynel Seferoğlu

**Affiliations:** 1 Gazi University, Department of Chemistry, Yenimahalle, Ankara, 06560, Turkey; 2 Gazi University, Department of Advanced Technology, Yenimahalle, Ankara, 06560, Turkey,; 3 Strasbourg University, Department of Ultra-Fast Optics and Nanophotonics, IPCMS, UMR CNRS, 7504, 67034 Strasbourg Cedex 2, France

**Keywords:** DFT calculations, NLO, pH sensitive dyes, Schiff base, solvent effect, styryl dyes

## Abstract

Herein we report the synthesis and characterization of a new series of styryl-based push-pull dyes containing a free amino group and their Schiff base derivatives. The dyes include the dicyanomethylene group as an acceptor and different *para*-substituted alkylamines as donors. Morever as a proton-sensitive group a pyridin-2-yl substituent was attached to the *para*-position of the phenyl moiety in both series of compounds. The photophysical properties of the dyes were examined in various solvents with different polarities and showed absorption in the visible region and green-red emission with low quantum yields. The absorption and the emission maxima were shifted bathocromically by increasing the solvent’s polarity. However, there was no correlation with the polarity parameters of the solvents. The pH-sensitive properties of all prepared Schiff bases were examined against TBAOH in DMSO, via deprotonation of the OH group in the salicylidene moiety and their reverse protonation was also investigated using TFA. The Schiff bases exhibited a bathochromic shift upon the addition of TBAOH to their solutions in DMSO. Therefore, they showed potential to be utilized as colorimetric and luminescence pH sensors. The second-order nonlinear optical (NLO) responses of the dyes were measured by the electric field-induced second harmonic (EFISH) generation method. The highest μβ values were obtained for the dyes bearing the julolidine donor as 1430 × 10^−48^ esu (for free amino derivative) and 1950 × 10^−48^ esu (for Schiff base derivative), respectively. The structural and electronic properties of the dyes as well as their NLO properties were further studied using DFT calculations. The thermal stabilities of all dyes were evaluated by thermogravimetric analysis (TGA). The TGA data showed that all dyes were thermally stable up to 250 °C.

## Introduction

Push-pull organic molecules are a class of organic dyes comprising of electron-donating and accepting groups in a donor–π–acceptor (D–π–A) system. The dyes exhibit strong absorption and emission properties in solution and in the solid state [1–3]. One important feature of these molecules is an exceptional polarizability which is a crucial criterion for NLO materials. Nowadays, organic NLO materials bearing strong donor–acceptor groups with a π-bridge have shown extensive usages in signal processing, optical storage, and telecommunication devices [4–10]. The flexibility to change electron-donor and acceptor groups in the molecules allows to tune intramolecular charge transfer (ICT) intensity and it can increase NLO properties [11–14].

Some ions such as proton (H^+^) and hydroxide (OH^−^), in aqueous environments, play significant roles in industrial, biological, and environmental processes [15–17]. Their presence in aqueous media directly determines the pH level that affects organisms living in the corresponding area [18–22]. Therefore, pH-sensitive dyes play a critical role in various sensor applications for easy determination of such ions. These dyes show different spectral properties upon protonation/deprotonation processes [23–25]. Fluorescent dyes as chemosensors offer unique merits such as low energy consumption, ease of handling, high selectivity, and notable sensitivity [26–27]. Moreover, some fluorescent dyes demonstrate remarkable ICT characteristics, and could therefore serve as NLO dyes [11–14].

Among dyestuffs classes, the push-pull fluorescent dyes are renowned to own such special behaviors. The push-pull dyes generate higher charge delocalization upon excitation, thus enhance both polarizability and fluorescence emission [12–14,18]. The charge delocalization upon excitation leads to a red-shifted emission which is viable for the detection of various substrates in biological tissues and samples [28–30]. Recently, there was an increasingly growing interest in studies regarding push-pull organic molecules comprising of the dicyanomethylene group as a strong electron-accepting group coupled with various donors connected via a π-conjugation bridge [31–33]. Such dyes offer good NLO characteristics when compared to Disperse Red 1 as well as remarkable thermal stabilities with dissociation temperatures up to 300 °C [31–32].

Schiff bases containing an azomethine group are one of the most widely used organic dyes because of their easy and cheap synthetic accessibility through various methodologies and suitable photophysical properties. In addition, they exhibit a broad range of biological activities such as antimicrobial, antifungal, antiviral, and anticancer activity, to name a few. Moreover, Schiff bases bearing a salicylidene moiety are also used as chemosensors for sensing of specific ions. In addition, some derivatives also show NLO activity [25,34–37].

Herein, we report on the synthesis and full photophysical characterization of a series of new styryl-based organic chromophores containing a free amino group and the corresponding Schiff base derivatives. The photophysical, pH sensitivity, NLO properties, and thermal stabilities of all synthesized dyes were investigated. Density Functional Theory (DFT) calculations were also employed to gain insight into the experimental data.

## Results and Discussion

### Synthesis

**Scheme 1:**
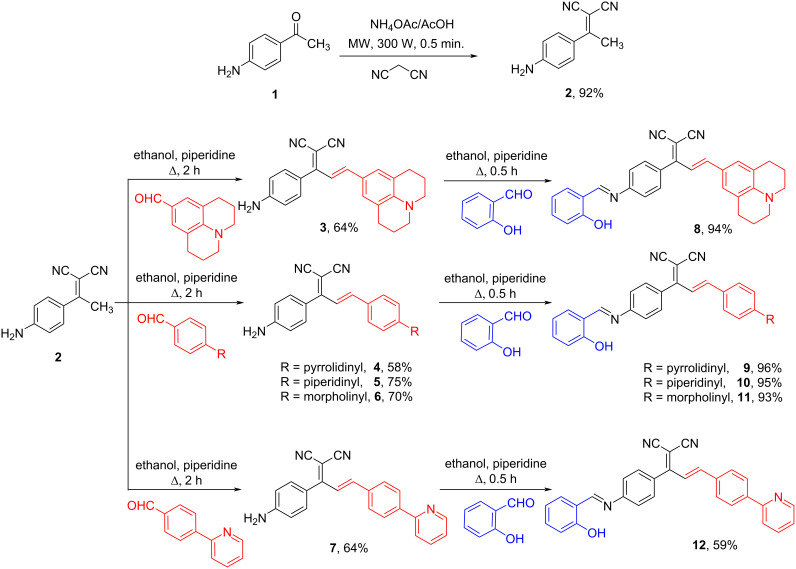
Synthetic pathways of dyes **3**–**7** and Schiff base analogs **8**–**12**.

As depicted in Scheme 1, dye **2** was obtained in good yield by reacting malononitrile and 4-aminoacetophenone (compound **1**) according to our previous published procedure [31–32].

The styryl-based organic chromophores **3**–**7** having a free amino and strong donor-acceptor groups and a pyridin-2-yl moiety were synthesized by the base-catalyzed condensation of **2** with different aldehyde derivatives in ethanol, as depicted in Scheme 1. Further reactions of the dyes **3**–**7** with salicylaldehyde under basic conditions resulted in the formation of an azomethine bridge on each amine end yielding dyes **8**–**12** as the final products. All dyes were obtained in moderate to excellent yields (58–96%) and generally without the need for chromatographic purification. The structures of all the new synthesized dyes were confirmed by FTIR, ^1^H, ^13^C NMR, and HRMS analyses (Figures S1–S40 in Supporting Information File 1). Moreover, the stereochemistry of the double bonds was determined on the basis of the coupling constants of the vinylic hydrogens in the ^1^H NMR spectra (*J* ≈ 15–16 Hz) and revealed that the dyes are stable as *E*-stereoisomers [31].

### Optimized geometries

**Figure 1:**
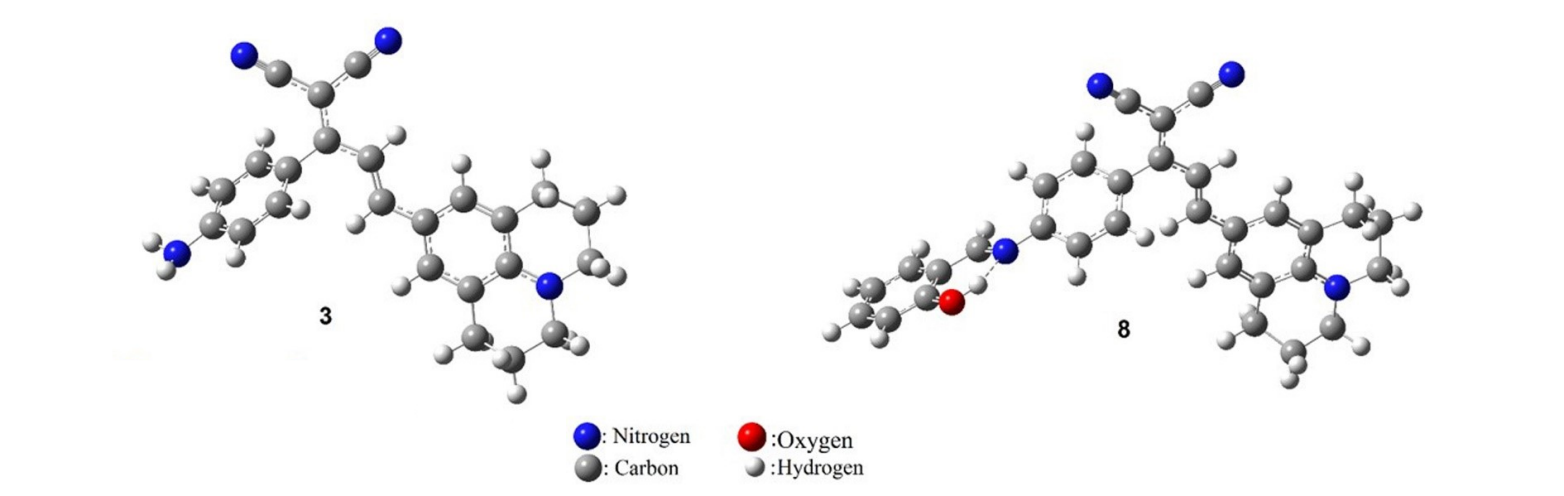
The optimized geometry of dyes **3** and **8**.

The optimized geometries of dyes **3**–**7** and **8**-**12** were obtained by performing DFT calculations at the B3LYP/6-31+G(d,p) level of theory. The structures are illustrated in Figure 1 and Figures S41 and S42 (Supporting Information File 1). As shown in Figure 1, the different substituents were planar to the dicyanomethylene group. A twist was observed between the dicyanomethylene and aminophenyl groups (salicylidenyl moiety for **8**–**12**) with an angle of about 50°. In addition, the dyes **8**–**12** exhibited strong intramolecular O–H···N hydrogen bonds with lengths in the range of 2.63–2.64 Å (Figure 1, Figures S41 and S42 in Supporting Information File 1).

### Absorption and emission properties

**Figure 2:**
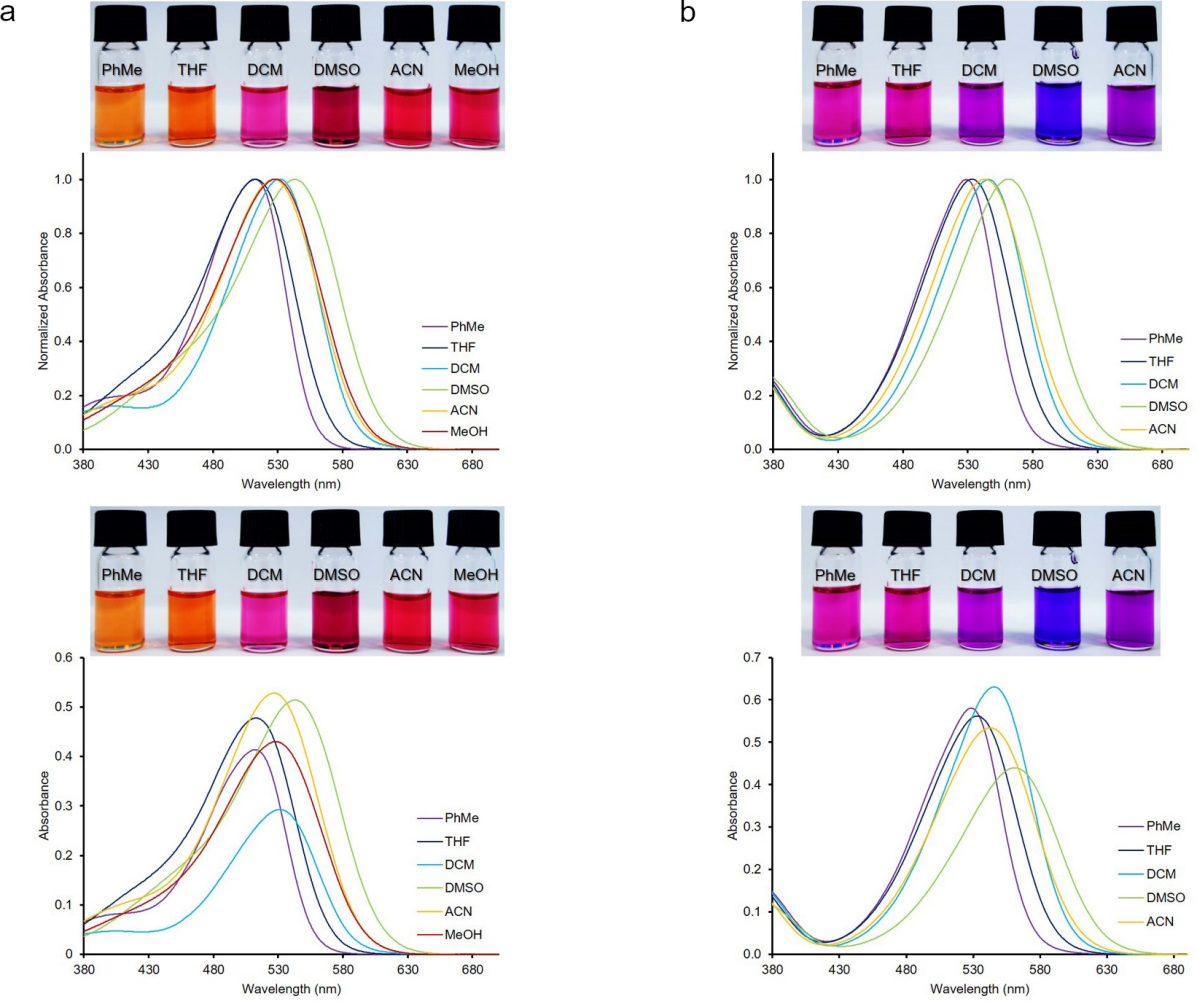
Absorption spectra of dyes **3** (a, left) and **8** (b, right). Inset: Color of dyes **3** and **8** in the given solvents of different polarities in ambient light, *c* = 10 μM.

**Figure 3:**
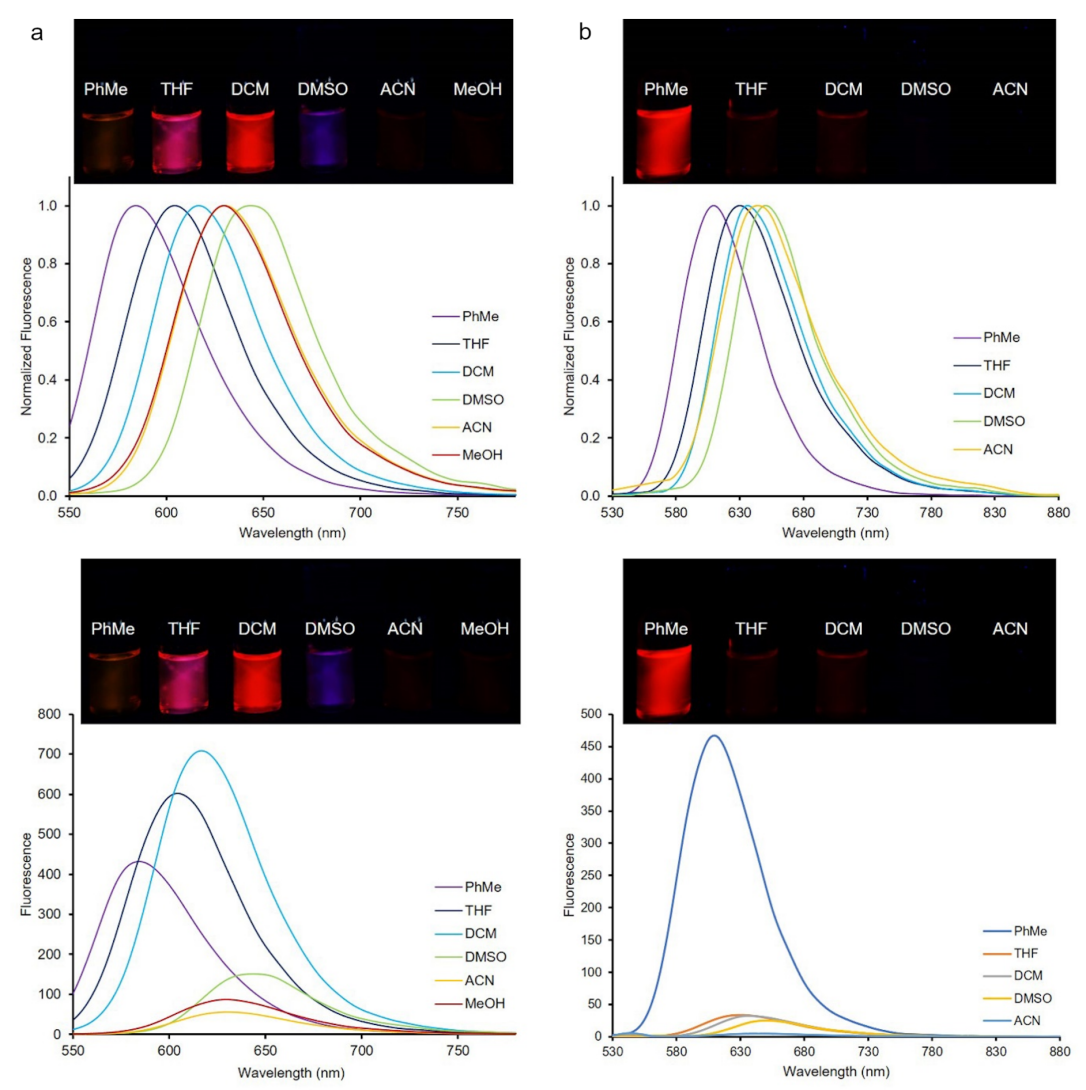
Emission spectra of dyes **3** (a, left) and **8** (b, right). Inset: Color of dyes **3** and **8** in the indicated solvents of different polarities under UV irradiation (λ_ex_ = 365 nm, *c* = 1 μM).

To assess the effect of the solvent on the absorption and emission spectra of **3**–**7** and **8**–**12** (*c* = 10 μM for absorption, and *c* = 1 μM for emission), various solvents with different polarity were employed at room temperature (Figure 2, Table 1, and Table S1 in Supporting Information File 1). The dyes showed generally good solubility in organic solvents. However, the dyes **8**–**12** had a low solubility in MeOH. Therefore, the photophysical properties of the dyes **8**–**12** could not be investigated in this solvent. The Dimroth–Reichardt polarity parameters (E_T_^30^, Dimroth–Reichardt polarity parameter in kcal mol^−1^ of MeOH = 55.4, ACN = 45.6, DMSO = 45.1, DCM = 40.7, THF = 37.4, and PhMe = 33.9) were used in the study [38]. In addition, the absorption spectra of all dyes were obtained by TD-DFT calculations using the PCM model. The absorption maxima (λ^abs^_max_), oscillator strengths (f), and relevant transitions and their contributions (w) are given in Table 1 and Table S1 in Supporting Information File 1). The shifts of the absorption maxima of the dyes were little dependent on the solvent polarity and there was no obvious correlation with increasing polarity parameters. There were only small differences observed for the absorption maxima values for both series of compounds. As representative examples, the spectra of **3** and **8** are provided in Figure 2 and Figure 3. The absorption spectra of **3** and **8** in all solvents tested displayed absorption maxima in the range of 512–543 and 529–562 nm, respectively. Of note, the largest bathochromic shifts of the absorption maxima for both series of dyes were observed in DMSO compared to the other solvents applied. Switching the solvent from PhMe to DMSO, bathochromic shifts of 31 nm for **3**, 22 nm for **4**, 19 nm for **5**, and 23 nm for **6** were observed. Similar behaviors were observed for the absorption spectra of compounds **8**–**12** (Table S1, Supporting Information File 1). The TD-DFT calculation results obtained for the values of absorption wavelengths for the dyes were in the order **3** > **4** > **5** > **6** > **7** and **8** > **9** > **10** > **11** > **12** in DMSO, which was consistent with the experimental data (Table 1 and Table S1 in Supporting Information File 1). A solvent effect on the absorption spectra was observed for compound **4** resulting in a bathochromic shift of about 22 nm from PhMe to DMSO, whereas there was only a little effect on the shift for dye **3** (6 nm). In case of the Schiff bases **8**–**11**, similar bathochromic shifts were observed. In addition, experimentally, there was no solvatochromic behavior observed for dyes **7** and **12** as was predicted by theoretical calculations.

**Table 1:** Photophysical properties of dyes **3**–**7** in various solvents with different polarity and the calculated absorption spectra data.

		Experimental						Calculated		

	solvent^a^	λ^abs^_max_ (nm)	λ^em^_max_ (nm)	Stokes shift (nm)	Stokes shift (cm^−1^)	Φ_F_^b^	ε (mM^−1^cm^−1^)	λ^abs^_max_ (nm)	*f*	transitions, w (%)

**3**	MeOH, (55.4^c^)	528	630	102	3074	<0.01	42.6	506	1.1731	HOMO→LUMO, 96.2
	ACN (45.6^c^)	527	630	103	3125	<0.01	51.7	507	1.1786	HOMO→LUMO, 96.2
	DMSO (45.1^c^)	543	644	101	2879	<0.01	49.8	511	1.2023	HOMO→LUMO, 96.6
	DCM (40.7^c^)	531	617	86	2632	0.01	36.8	506	1.1985	HOMO→LUMO, 96.5
	THF (37.4^c^)	513	604	91	2961	0.01	45.9	504	1.1916	HOMO→LUMO, 96.4
	PhMe (33.9^c^)	512	584	72	2414	<0.01	41.4	494	1.2083	HOMO→LUMO, 96.6

**4**	MeOH	498	606	108	3568	<0.01	55.4	491	1.1403	HOMO→LUMO, 89.9
	ACN	495	611	116	3819	<0.01	48.6	492	1.1469	HOMO→LUMO, 90.2
	DMSO	510	622	112	3515	<0.01	49.6	495	1.1750	HOMO→LUMO, 91.3
	DCM	502	589	87	2954	0.01	30.1	490	1.1746	HOMO→LUMO, 90.9
	THF	486	582	96	3403	<0.01	33.7	488	1.1674	HOMO→LUMO, 90.5
	PhMe	488	558	70	2592	<0.01	41.9	478	1.2037	HOMO→LUMO, 91.1

**5**	MeOH	476	609	133	4599	<0.01	39.9	488	1.0314	HOMO-1→LUMO, 15.2 HOMO→LUMO, 84.6
	ACN	473	611	134	4770	<0.01	35.9	489	1.0384	HOMO-1→LUMO, 14.8 HOMO→LUMO, 85.0
	DMSO	486	624	138	4561	<0.01	42.1	492	1.0686	HOMO-1→LUMO, 13.2 HOMO→LUMO, 86.7
	DCM	483	592	109	3828	0.01	25.3	487	1.0750	HOMO-1→LUMO, 13.4 HOMO→LUMO, 86.4
	THF	457	583	126	4741	<0.01	31.4	485	1.0687	HOMO-1→LUMO, 13.9 HOMO→LUMO, 85.9
	PhMe	467	557	90	3476	<0.01	33.9	476	1.1242	HOMO-1→LUMO, 12.2 HOMO→LUMO, 87.5

**6**	MeOH	446	600	154	5786	<0.01	40.8	451	0.5104	HOMO-1→LUMO, 62.3 HOMO→LUMO, 37.0
	ACN	442	603	161	6052	<0.01	34.6	451	0.5053	HOMO-1→LUMO, 63.2 HOMO→LUMO, 36.1
	DMSO	463	614	151	5346	<0.01	42.7	453	0.4843	HOMO-1→LUMO, 66.7 HOMO→LUMO, 32.6
	DCM	448	580	132	5099	<0.01	27.8	451	0.4988	HOMO-1→LUMO, 63.2 HOMO→LUMO, 36.1
	THF	439	573	134	5359	<0.01	34.7	450	0.5077	HOMO-1→LUMO, 61.4 HOMO→LUMO, 37.9
	PhMe	440	546	106	4399	<0.01	30.6	446	0.4885	HOMO-1→LUMO, 57.8 HOMO→LUMO, 41.4

**7**	MeOH	374	–	–	–	–	51.4	419	1.2899	HOMO-1→LUMO, 95.0
	ACN	376	–	–	–	–	43.4	419	1.2939	HOMO-1→LUMO, 95.0
	DMSO	386	–	–	–	–	40.4	422	1.3123	HOMO-1→LUMO, 95.2
	DCM	382	606	224	9705	n.d.	42.1	421	1.3265	HOMO-1→LUMO, 94.0
	THF	379	434	55	3357	n.d	40.4	420	1.3246	HOMO-1→LUMO, 93.7
	PhMe	382	577	195	8853	n.d	53.1	419	1.3788	HOMO-1→LUMO, 88.8

^a^Solvents arranged in order of decreasing E_T_^30^ values. ^b^Fluorescence quantum yield (±10%) determined relative to fluorescein in pH 9 solution (Φ_F_ = 0.95) as standard. ^c^The values for relative polarity are taken from [38]. n.d, could not be determined.

Based on theoretical calculations, the main peaks were found to correspond to transitions from the highest occupied molecular orbital (HOMO) to the lowest unoccupied molecular orbital (LUMO) with the highest contributions from **3**–**5** and **8**–**11**. For dyes **6**, **7**, and **12**, the highest contributions were from transitions HOMO-1→LUMO. The picture of the frontier molecular orbitals of the dyes are shown in Figure S92 in Supporting Information File 1.

Solvatochromic studies on emissions were also done to gain insights into the photophysical behavior of the new push-pull dyes bearing free amino and azomethine groups. Therefore, the fluorescence spectra of all dyes **3**–**7** and **8**–**12** were recorded in the same solvents of various polarities that were used in the UV–vis studies, and the results are summarized in Table 1 and Table S1 (Supporting Information File 1). All dyes showed fluorescence properties and there was no direct correlation of the increase of maximum emission wavelengths with increasing polarity parameters of the solvents. However, the largest bathochromic shifts of the emission maxima for both series of dyes were observed in DMSO. This phenomenon is observed when highly fluorescent polar excited states of push-pull dyes are stabilized by solvents with different polarity [39–45]. The emission spectra of the dyes **3** and **8** and the color changes observed upon UV irradiation of both dyes in various solvents are shown in Figure 3. Both series of the dyes showed fluorosolvatochromic behavior in all solvents used except for MeOH and ACN, and the emission band of dye **3** was red-shifted by 60 nm from PhMe (λ^em^_max_ = 584 nm) to DMSO (λ^em^_max_ = 644 nm) (Figure 3). The dyes showed considerably different fluorescence color magnitudes with changing polarity except for compound **12** that was non-fluorescent. The fluorescence intensity of all dyes was high in solvents with low polarities such as PhMe and THF, while it decreased with increasing solvent polarities such as DMSO and MeOH. The fluorescence quantum yields of the dyes were found to be very low as compared to fluorescein (Φ_F_= 0.95 at pH 9). For example, the quantum yield for **3** in DCM was 0.01 and that of **8** in PhMe was 0.11.

### Substituent effects

**Figure 4:**
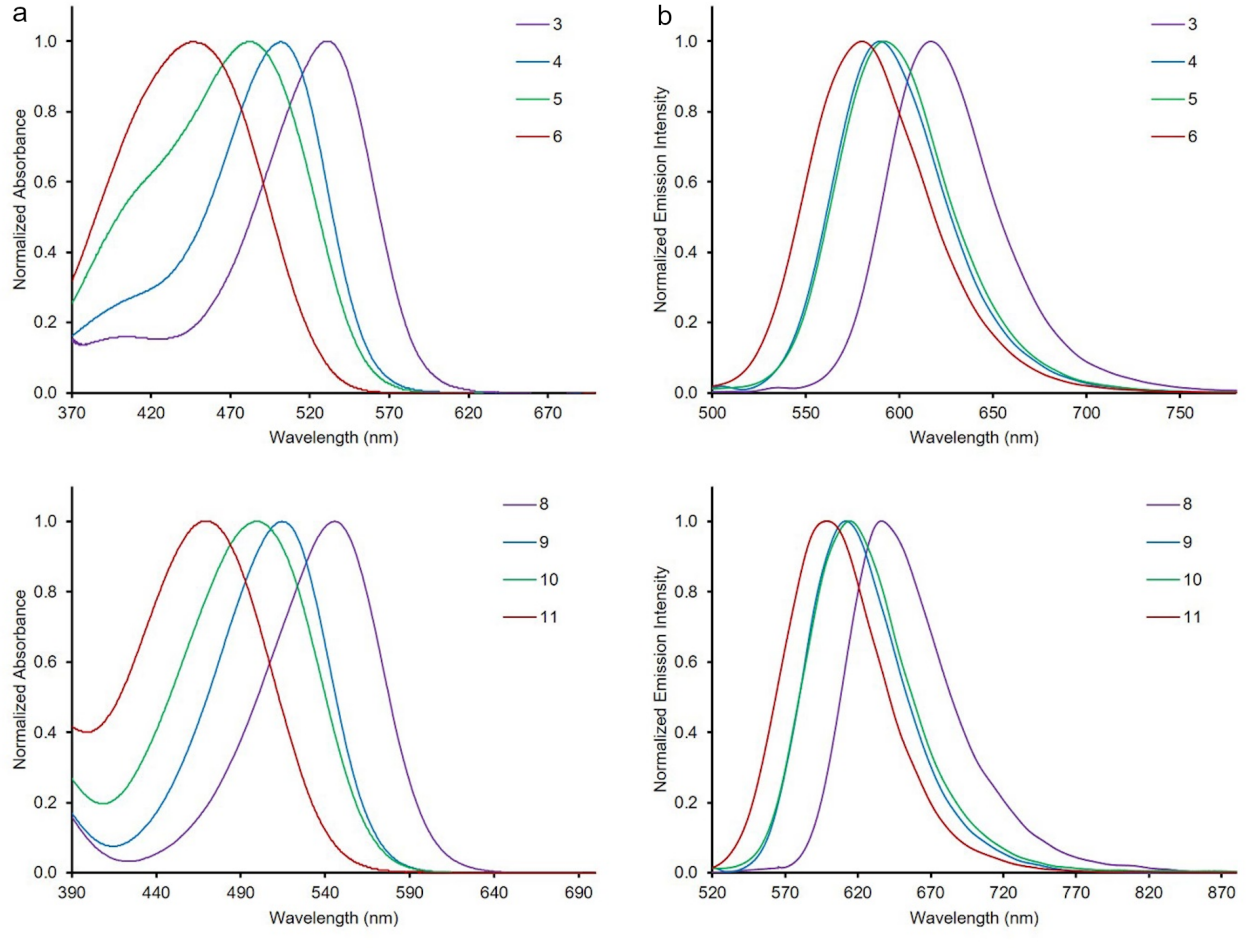
Red shift phenomena with changing substituents in absorption (a, left) and emission (b, right) spectra of dyes **3**–**6** (top) and **8**–**11** (bottom) in DMSO.

The prepared Schiff bases exhibited greater red shifts than the styryl counterparts (**3**–**7**) in both, absorption and emission spectra. For instance, in DCM, dye **8** showed absorption and emission maxima at 546 and 636 nm, whereas the maxima of dye **3** were observed at 531 and 617 nm, respectively. Moreover, both the styryl and the Schiff bases showed greater red shifts compared to the previously reported counterparts [31–32]. As can be seen from Figure 4, the absorption maxima of dyes **3**–**6** and **8**–**11** depended on the electron-donating nature of the substituents, and the values of the maximum absorption wavelengths increased in the following order: 4-morpholinyl (**6**, **11**) < 4-piperidinyl (**5**, **10**) < 4-pyrolidinyl (**4**, **9**) < 4-julolidinyl (**3**, **8**). The dyes **3** and **8** containing a julolidinyl group exhibited the most pronounced red shift while the dyes containing a morpholinyl substituent (**6** and **11**) showed the least one. In addition, dyes **7** and **12** bear a proton-sensitive and electron-accepting pyridin-2-yl group and therefore, the absorption maxima for dyes **7** and **12** were lower than the maximum absorption wavelengths of the other dyes. The maximum absorption wavelengths of these dyes were in the range of 374–386 nm and 377–385 nm. Moreover, almost the same trends which were obtained from the UV–vis absorption spectra were also observed in the emission maxima. The photophysical properties of dyes **3**–**7** were similar to those of the corresponding Schiff base derivatives **8**–**12**. However, the Schiff bases **8**–**12** had higher maximum absorption and emission wavelength values than the dyes **3**–**7** because of the extended conjugation system present in the Schiff bases.

The observation of an intense red shift might be related to a significant contribution of ICT for electronic state modification in the excited state of the molecules [45]. In this state, all dyes have a notably higher dipole moment and a lower LUMO energy level as compared to their ground state [46–49]. Increasing the electron-donating strength of the various heterocyclic amines led to the increase in the Stokes shift and a significant red shift between the absorption and the emission maxima. This was due to the varying electronic contributions to the host structure induced by the heterocyclic amino constituents [50–52] (Figure 4).

### Hydroxide anion sensing properties

**Figure 5:**
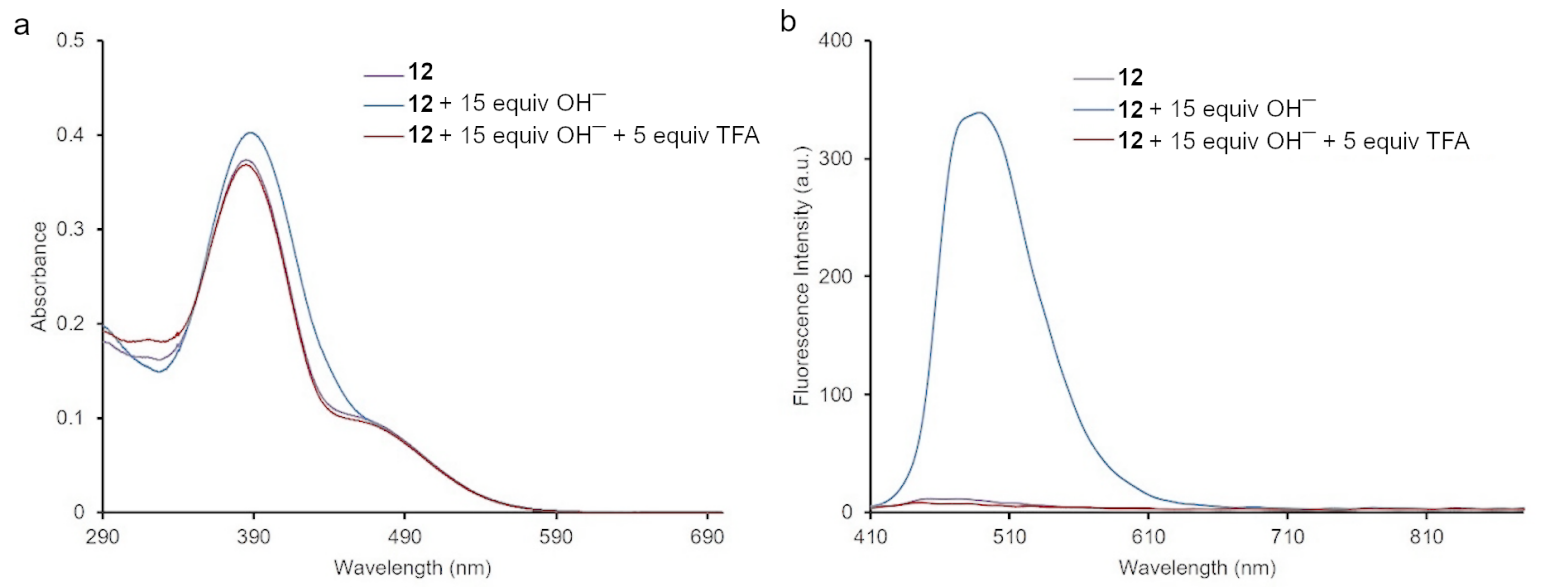
Absorption (a, left) and emission (b, right) change of dye **12** upon addition of 15 equiv of TBAOH and reverse protonation by adding 5 equiv of TFA in DMSO solution.

**Figure 6:**
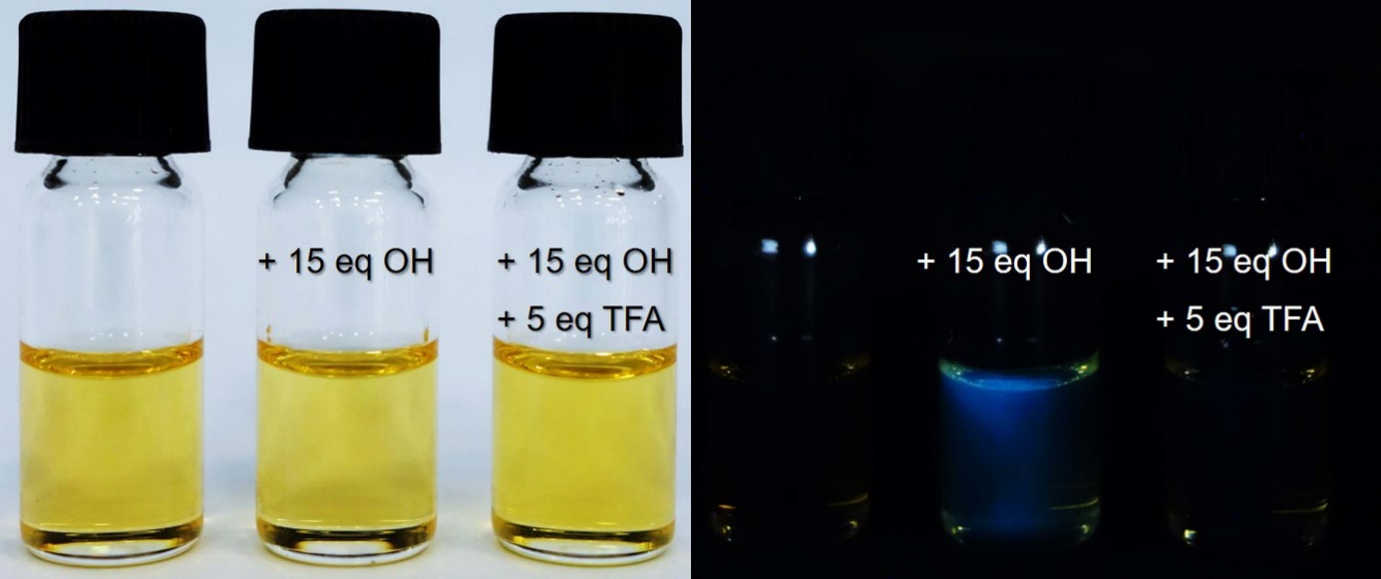
Photographs of dye **12** (left, ambient light), without, after the addition of 15 equiv of TBAOH (middle), and reverse protonation by 5 equiv of TFA in DMSO solution (right photograph: the same solutions under UV light (UV lamp, λ_ex_ = 365 nm)).

The dyes **8–12** could have the ability to detect hydroxide anions due to the presence of an OH substituent at the salicylidene moiety. Therefore, the OH^−^ sensing activity of compounds **8–12** was investigated by the addition of hydroxide anions in the form of the corresponding tetrabutylammonium (TBA) salt. An interaction between the dyes and hydroxide anion was investigated in DMSO as the solvent. The presence of hydroxide led to small changes in the UV–vis absorption spectra of the dyes. As presented in Figure 5, the interaction of dye **12** with the hydroxide anion, led to a small hyperchromic effect in the UV–vis absorption spectrum. Moreover, dyes **8**–**12** showed an enhancement in fluorescence intensity upon the incremental addition of hydroxide anions to their solutions in DMSO. As shown in Figure 5 and Figure 6, dye **12** showed the highest increase in fluorescence (approx. a 20-fold fluorescence enhancement) upon addition of 15 equiv OH^−^, thus reflecting its superior sensitivity among the others (Supporting Information File 1). The increased emission intensity and the formation of a new absorption band could likely be due to the formation of a phenoxide group, which is a more pronounced electron donor than the phenol group [52–53]. Furthermore, trifluoroacetic acid (TFA) was added to a solution containing the deprotonated forms of dyes **8**–**12** in DMSO, so as to investigate the reversible protonation of the dyes. As can be seen in Figure 5, the addition of 5 equiv of TFA to a basic solution of **12** led to the restoration of its emission intensity. Figure 6 shows the deprotonation and reverse protonation of dye **12** by color changes of the dye followed under a UV lamp (λ_ex_ = 365 nm). Since under the ambient light all solutions seemed to have the same color, a UV lamp was used to observe the color changes. As shown in Figure 6, the color of the free dye **12** dissolved in DMSO was light yellow. The addition of 15 equiv of TBAOH to the solution led to a change of the colorless solution to blue under UV light, whereas no color change was observed in ambient light. The observed color change under UV light indicated that the dye was deprotonated. Indeed, the addition of only 5 equiv of TFA regenerated the light yellow color of the solution indicative for the reversed protonation of **12**. All dyes, except **8**, showed this reversibility. Furthermore, the limits of detection (LOD) and quantification (LOQ) were determined for dyes **8**–**12**. The LOD and the LOQ were respectively found to be as follow: 73.1 μM and 244 μM for **8**, 47.2 μM and 157 μM for **9**, 47.3 μM and 158. μM for **10**, 50.9 μM and 170 μM for **11**, 33.1 μM, and 110 μM for **12** (Figures S87–S91, Supporting Information File 1) [25].

### pH-Sensitive study and p*K*_a_ determination of **8** in aqueous solution

The pH value of aqueous systems is important to maintain the physiological stability, both inside and outside a living organism. The hydroxide anion plays a special and critical role in the ecosystem. Therefore, the necessity to develop new pH sensors is vital to control pH conditions in various environments. Due to their low-cost of manufacturing and maintenance, pH sensors were developed and used for both, clinical and environmental analyses [54–56]. Nevertheless, fluorescent pH indicators offer higher selectivity and sensitivity than other classes [28]. Therefore, we investigated dye **8** as a prospect for a fluorescent pH sensor in a basic environment.

**Figure 7:**
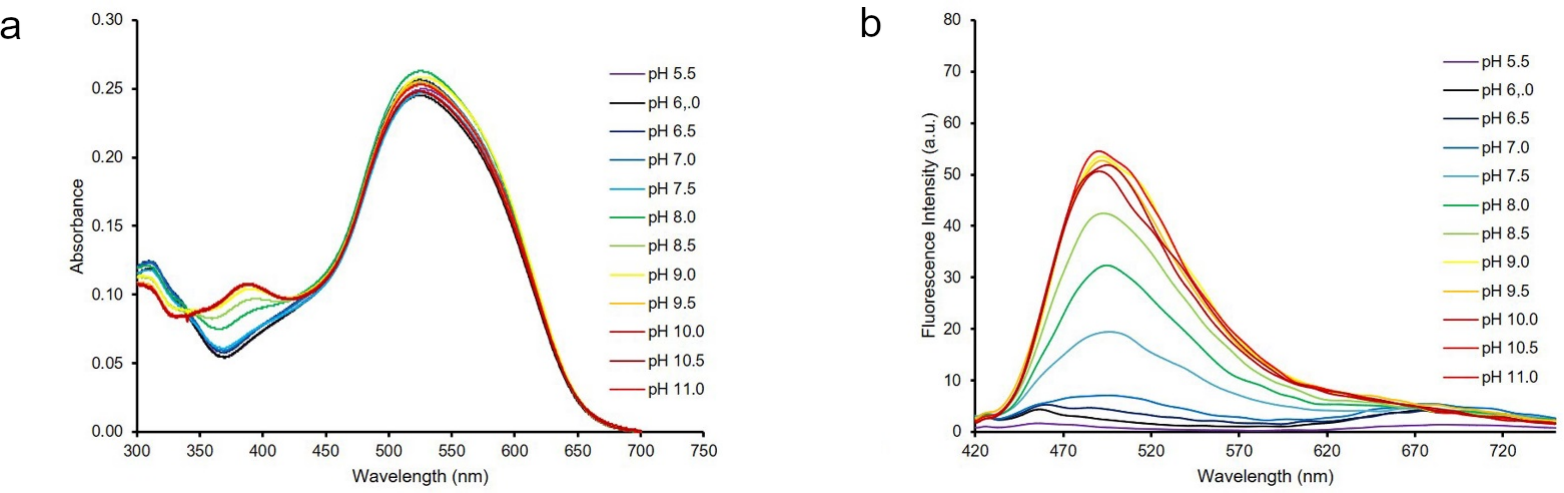
Absorption (a, left) and emission (b, right) change of **8** in Britton–Robinson buffer solutions at different pH values.

**Figure 8:**
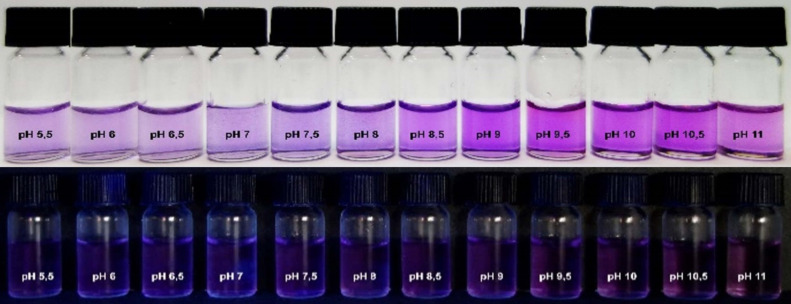
Photographs of dye **8** in Britton–Robinson buffer solutions at different pH values.

**Figure 9:**
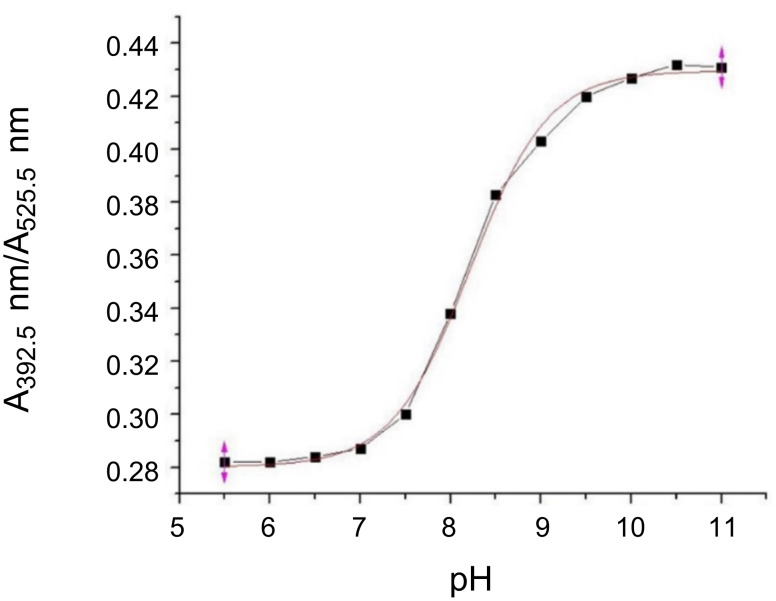
Sigmoid function obtained from dye **8** UV–vis absorption spectra during pH investigation.

After having determined the solubility of dye **8** in aqueous solution, the sensing capability of **8** toward hydroxide anions was tested. Several Britton–Robinson buffer solutions, with different pH values ranging from 5.5–11, were used to mimic basic environments and to preserve pH stability in solution. As depicted in Figure 7, increasing the pH value of the solution led to the formation of a new absorption band at 393 nm (in UV region) and a weak fluorescence increase at 505 nm. This phenomenon was accompanied by darkening purple color shifts of the solutions with increased pH value. However, the slight increase in the fluorescence was hardly noticeable below 365 nm UV light as is shown in Figure 8. The p*K*_a_ of **8** was calculated by using a spectrophotometric method and was 8.22 ± 0.03 (Figure 9).

### NLO properties

The second-order NLO responses of all prepared dyes were measured by the EFISH technique in CHCl_3_ solution with a nonresonant incident wavelength of 1907 nm. Experimental details on the EFISH measurements are given elsewhere [57]. EFISH measurements provide information about the scalar product, μβ(2ω), of the vector component of the first hyperpolarizability tensor, β*,* and the dipole moment vector [58–61]. This product is derived according to Equation 1 considering γ_0_(−2ω,ω,ω,0), the third-order term, as negligible for the push-pull dyes under consideration. This approximation is usually used for push-pull organic and organometallic molecules.

[1] $${\text{γ}}_{\text{EFISH}}=\mu \beta /5kT+{\text{γ}}_{0}(-2\text{ω}\text{,ω}\text{,ω},0)$$

The results of the EFISH measurements are presented in Table 2. The obtained positive µβ values indicated that the excited states are more polarized than the ground states and that both, the ground and the excited states were polarized in the same direction for all dyes. All studied dyes exhibited a higher NLO response than Disperse Red 1, which is generally used as a reference (µβ = 450 × 10^−48^ esu), except for dyes **7** and **12**. For the latter two compounds (**7** and **12**), relatively low µβ values were observed. However, the dyes **8–12** exhibited a higher NLO response than their analogs **3–7**.

**Table 2:** Experimental and calculated NLO properties and energy gap values for dyes **3**–**12**.

Compound	Δ*E*^a^ (eV)	µβ^b^ (× 10^−48^) (esu)	µ^a^ (Debye)	β^a^ (× 10^−30^) (esu)	Compound	Δ*E*^a^ (eV)	µβ^b^ (× 10^−48^) (esu)	µ^a^ (Debye)	β^a^ (× 10^−30^) (esu)

**3**	2.64	1430	19.42	337	**8**	2.56	1950	19.45	354
**4**	2.77	1150	18.64	320	**9**	2.68	1540	18.56	342
**5**	2.76	1080	18.59	339	**10**	2.70	1340	17.18	363
**6**	2.83	600	16.07	314	**11**	2.74	800	17.59	328
**7**	3.00	260	15.62	141	**12**	3.12	250	14.00	176

^a^DFT results at the B3LYP/6-31+G(d,p) level of theory in CHCl_3_. ^b^EFISH: µβ (2ω) at 1907 nm in CHCl_3_, molecular concentrations used for the measurements were in the range of 10^−3^ to 10^−2^ M. µβ ± 10% (for **3**–**11**), µβ ± 50% (for **12**); esu: electrostatic unit.

In order to obtain an insight into the NLO properties of the studied dyes, the first-order hyperpolarizability (β) and dipole moment (µ) were calculated at the B3LYP/6-31+G(d,p) level of theory in CHCl_3_. In general, a high NLO response for a typical organic NLO chromophore is related to the presence of donor (D) and acceptor (A) groups linked through a π-conjugation path and is characterized by a large first-order hyperpolarizability value (β). However, a small energy gap between the HOMO and the LUMO (*E*_gap_) is an important indicator for high NLO responses. Based on the results in Table 2, the *E*_gap_ values obtained from the ground state geometries of the dyes changed as: **3** < **4** ≈ **5** < **6** < **7** for dyes **3**–**7** and **8** < **9** ≈ **10** < **11** < **12** for dyes **8**–**12**. The smallest *E*_gap_ was obtained for dyes **3** and **8** due to the presence of the strongest electron-donor group (julolidine), and a high NLO response could be expected. Indeed, dyes **3** and **8** had the highest µ and β values while **7** and **12** had the lowest ones. It should be noted that the hyperpolarizability values were dominated by the component β_xxx_ (for **3**–**7**, **10**, and **11**) and β_xxy_ (for **8**, **9**, and **12**), which indicated a substantial delocalization of charges in this direction (Tables S2 and S3, Supporting Information File 1).

### Thermal properties

**Figure 10:**
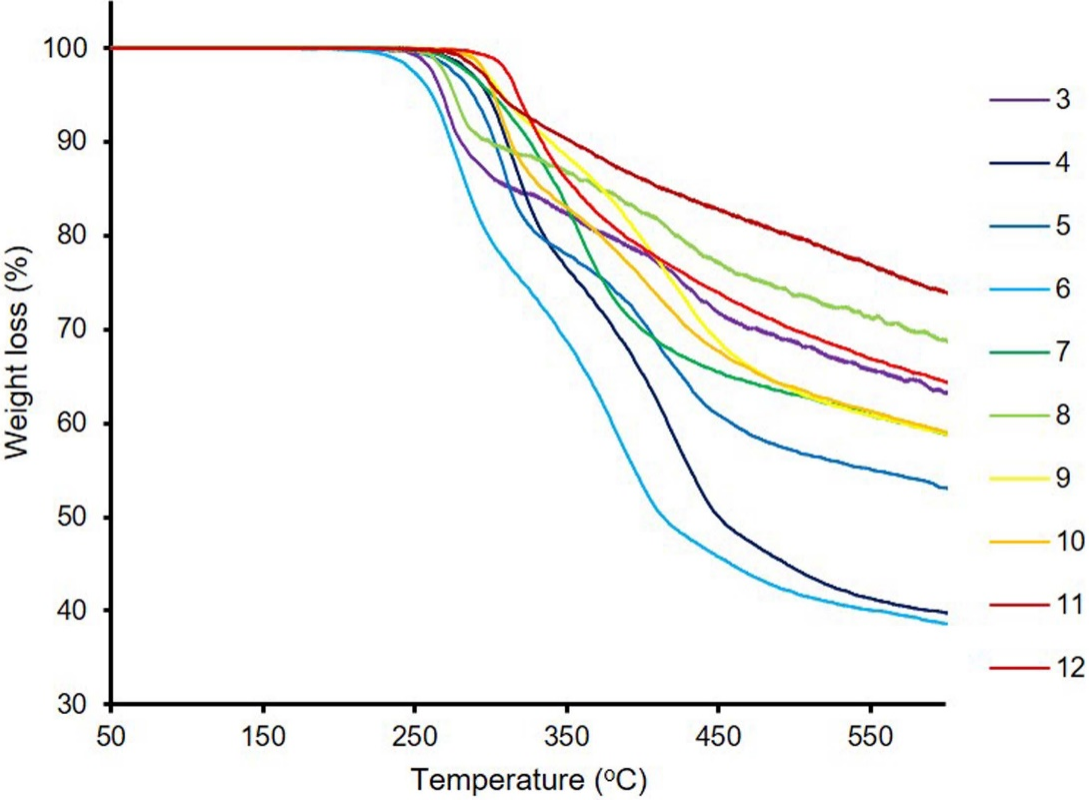
TGA curves of all synthetized dyes.

The thermal stability is quite important for NLO chromophores when using them in electro-optic materials. Therefore, we conducted a thermogravimetric analysis to understand whether the dyes were suitable as NLO candidate chromophores. NLO candidate chromophores must be thermally stable (>200 °C) and should have a high thermal dissociation value to be applied in electric field poling [62]. The thermal properties of the chromophores were evaluated by thermal gravimetric analysis (TGA) under a nitrogen atmosphere and the results are shown in Figure 10. As depicted in Figure 10, mass wise, all the dyes show noncomplete dissociation under inert environment up to 600 °C. The dyes showed zero weight loss from 0 up to 150 °C which indicated that there was no water or other organic solvent residues on the surface of the dyes.

Again, the results showed that no dye underwent more than one dissociation step. All dyes dissociated at the very first step but in a nonuniform manner. Regardless, all dyes showed good thermal stability up to around 250 °C, while dye **12**, as the least thermally stable dye, started to dissociate at around 310 °C. The Schiff base-based dyes had higher thermal stabilities than the dyes **3**–**7** having a free amino group. The highest thermal stability was observed for dyes **10**–**12**; their rapid degradation values were above 300 °C.

## Conclusion

In summary, we successfully synthesized and characterized a series of new push-pull styryl dyes with free amine functionalities and the corresponding Schiff base analogus. The Schiff bases were found to be able to detect hydroxide anions in DMSO and therefore could act as a pH sensor in a fully aqueous environment. The dyes **8–12** showed increasing fluorescence intensity upon the incremental addition of hydroxide anions in DMSO. Among them, dye **12** showed the highest fluorescence increase (approx. 20-fold fluorescence enhancements upon addition 15 equiv of TBAOH) marking for its superior sensitivity among the other dyes. Furthermore, a reversibility test using TFA showed that all Schiff base-based dyes, except **8**, could be brought to the initial state and hence marked their repeatability usage for every single analysis process. The interaction of a Schiff base and hydroxide anion in aqueous environment was conducted successfully using dye **8**, which had good solubility in water and a slight rise in fluorescence was observed with increasing pH of the solution.

In addition, the NLO properties of dyes **3**–**6** and **8**–**11** were investigated experimentally and theoretically. The dyes **3** and **8** bearing a julolidinyl donor exhibited the highest NLO response (μβ = 1430 × 10^−48^ esu and 1950 × 10^−48^ esu, respectively). Thermogravimetric analysis was conducted to understand the thermal stability of the synthesized dyes. In general, all dyes showed good thermal stability up to around 250 °C.

In conclusion, the results indicate that the styryl-based new push-pull dyes are promising candidate materials for optoelectronic device usages and various NLO applications and could be used for applications as water-soluble fluorescent probes for determination of pH.

## Experimental

### Materials, methods, and instrumentation

Tetrabutylammonium hydroxide (TBAOH) was purchased from Sigma-Aldrich. The solvents were of analytical grade and commercially available chemicals were purchased from Alfa Aeser Chemicals and used without further purification. FTIR (ATR) spectra were recorded on a Thermo Scientific Nicolet iS5 FTIR spectrometer. NMR spectra were recorded on a Bruker Avance 300 (^1^H: 300 MHz, ^13^C: 75 MHz) spectrometer at 20 °C (293 K). Chemical shifts (δ) are given in parts per million (ppm) using the residual solvent peaks as reference relative to TMS. Coupling constants (*J*) are given in hertz (Hz). Signals are abbreviated as follows: broad, br; singlet, s; doublet, d; doublet-doublet, dd; doublet-triplet, dt; triplet, t; multiplet, m. High-resolution mass spectra (HRMS) were recorded at Gazi University Faculty of Pharmacy using electron ionization (EI) mass spectrometry (Waters-LCT-Premier-XE-LTOF (TOF-MS) instruments; in *m*/*z* (rel. %). Elemental analysis was performed using a Thermo Scientific Flash 2000 analyzer at the Gazi University Department of Chemistry. The microwave syntheses were carried out in a Milestone Start microwave reaction system. The melting points were measured using an Electrothermal IA9200 apparatus. Absorption spectra were recorded on a Shimadzu 1800 spectrophotometer; fluorescence spectra were recorded on a Hitachi F-7000 fluorescence spectrophotometer. Thermal analyses were performed with a Shimadzu DTG-60H system, up to 700 °C (10 °C min^−1^) under a dynamic nitrogen atmosphere (15 mL min^−1^).

### Photophysical studies

Dyes **3–7** (10 µM for absorption and 1 µM for emission) were studied in six various solvents with different polarity (toluene, THF, DCM, DMSO, ACN, and MeOH), whereas dyes **8–12** were studied in the same solvents, however, excluding MeOH due to the low solubility of dyes **8–12** in this solvent. All samples were measured in quartz cuvettes (1 cm × 1 cm) with approximately 2 mL in volume. The deprotonation and reverse protonation studies of dyes **8–12** were conducted in DMSO. To each DMSO solution, 5 equiv of TBAOH were introduced and immediately the UV–vis absorption spectra taken at the same concentration mentioned above. This titration was repeated until there was no observable change in the sample spectra. The titrant used in this titration was 10 mM TBAOH in DMSO. The reverse protonation process for the dyes **8–12** was conducted by adding 10 mM TFA to the DMSO solution of the dye and TBAOH. Five equiv of TFA were introduced to fully achieve reprotonation. All of the procedures described above were performed at room temperature (25 °C). Emission changes during the deprotonation and reverse protonation processes for dyes **8–12** were also recorded using a fluorimeter in the same manner with the same concentration as stated earlier for the UV–vis absorption study at room temperature (25 °C). All photographs were taken using a SONY RX100 pocket camera with ISO values of 200 and variable aperture at “Program Auto” mode.

### p*K*_a_ Determination of dye **8**

The solubility of dye **8** in deionized water was investigated by studying its calibration graph at 505 nm. The calibration graph was obtained using UV–vis absorption spectrophotometry by the following procedure: A 5 µM solution of **8** was prepared and immediately its absorbance at 505 nm recorded. In the same solution, 10 µL from a 1 mM stock solution of dye **8** was added incrementally, followed by recording its absorbance value after each addition. From the results, the absorbance vs concentration graph was drawn in order to obtain the calibration graph. The linearity was investigated by calculating the R^2^ value using Microsoft Excel. The spectra of dye **8** were recorded using a Shimadzu 1800 UV–vis spectrophotometer in Britton–Robinson buffer solutions [63] with pH values ranging from 5.5 to 11 at room temperature (25 °C). The p*K*_a_ values were obtained using the Origin software by recording the curve data.

### Computational methods

The geometries of the dyes in their ground states were calculated by Density Functional Theory at the B3LYP/6-31+G(d,p) level of theory in the gas phase [64–65]. This method was also applied to study the nonlinear optic (NLO) properties of the dyes in their ground states. The theoretical absorption spectra in different solvents were calculated using the time dependent DFT (TD-DFT) method at the same level of theory. For calculations in solvents, the Polarizable Continuum Model (PCM) was used [66–67]. All calculations were done using the Gaussian 09 program [68].

### General synthetic procedure for dyes **2** and **3–7**

The protocol for the synthesis of dye **2** was published previously [31–32]. Dyes **3–7** were synthesized using **2** and the appropriate benzaldehyde. Equimolar (3 mmol) amounts of 2-(1-(4-aminophenyl)ethylidene)malononitrile (**2**) and the appropriate benzaldehyde derivative in 20 mL of ethanol were refluxed for 2 h. A colored solid formed for all dyes **3–7** which was collected by filtration and recrystallized from ethanol to obtain the pure dyes.

((*E*)-2-(1-(4-Aminophenyl)-3-(2,3,6,7-tetrahydro-1*H*,5*H*-pyrido[3,2,1-*ij*]quinolin-9-yl)allylidene)malononitrile) (**3**)


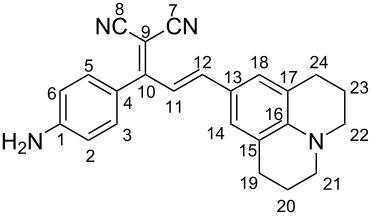


Dark purple solid; yield: 64%; mp 236–238 °C; FTIR (cm^−1^): 3441, 3349, 3224, 2920, 2836, 2202, 1633, 1603, 1568, 1519, 1467; ^1^H NMR (300 MHz, DMSO-*d*_6_) δ 7.15 (d, *J* = 8.5 Hz, 2H), 7.05 (m, 3H), 6.85 (d, *J* = 14.9 Hz, 1H), 6.65 (m, 2H), 5.96 (s, 2H), 3.27 (t, 4H), 2.66 (t, 4H), 1.84 (p, *J* = 6.2 Hz, 4H); ^13^C NMR (75 MHz, DMSO-*d*_6_) δ 171.7 (C10), 153.5 (C1), 153.4 (C16), 149.1 (C3-C5), 132.3 (C12), 131.2 (C11), 124.8 (C13), 120.6 (C4), 120.0 (C14-C18), 116.5 (C15-C17), 115.7 (C7), 114.5 (C8), 113.6 (C2-C6), 72.4 (C9), 66.3 (C21-C22), 47.3 (C19-C24), 44.6 (C20-C23); HRMS (*m*/*z*): [M – H]^+^ calcd for C_24_H_22_N_4,_ 367.1923; found, 367.1918; anal. calcd for C_24_H_22_N_4_, C, 78.66; H, 6.05; N, 15.29; found: C, 78.58; H, 6.09; N, 15.18.

### General synthetic procedure for dyes **8–12**

For the preparation of dyes **8–12**, a mixture of 1 mmol of the appropriate styryl dye **3**–**7** and 8 mmol of salicyaldehyde in 10 mL ethanol was refluxed for half an hour. The colored solid was then collected by filtration and recrystallized from ethanol to obtain the pure dyes.

(2-((*E*)-1-(4-(((*E*)-2-Hydroxybenzylidene)amino)phenyl)-3-(2,3,6,7-tetrahydro-1*H*,5*H*-pyrido[3,2,1-*ij*]quinolin-9-yl)allylidene)malononitrile) (**8**)


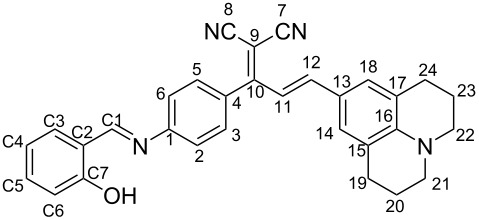


Dark purple solid; yield 94%; mp 202–203 °C; FTIR (cm^−1^): 3479 (broad), 3062, 2943, 2835, 2208, 1614, 1560, 1510, 1469; ^1^H NMR (300 MHz, DMSO-*d_6_*) δ 12.80 (s, 1H), 9.05 (s, 1H), 7.70 (d, *J* = 7.6 Hz, 1H), 7.52 (m, 5H), 7.09 (m, 5H), 6.75 (d, *J* = 14.9 Hz, 1H), 2.65 (t, 4H), 1.83 (m, 4H); ^13^C NMR (75 MHz, DMSO-*d*_6_) δ 171.0 (C10), 165.2 (C1’), 160.8 (C7’), 153.6 (C1), 150.8 (C16), 150.3 (C3-C5), 136.9 (C5’), 134.2 (C3’), 133.1, 132.3 (C12), 132.1 (C11), 131.0, 123.5 (C13), 123.0 (C2’), 122.3 (C4’), 119.8 (C4), 118.9 (C14-C18), 117.2 (C15-C17), 115.4 (C7), 114.6 (C8), 114.5 (C2-C6), 114.4, 114.3 (C6’), 95.7, 75.8 (C9), 48.1 (C21-C22), 25.4 (C19-24), 24.4 (C20-C23). HRMS (*m*/*z*): [M − H]^+^ calcd for C_31_H_26_N_4_O, 471.2185; found, 471.2162; anal. calcd for C_31_H_26_N_4_O; C, 79.12; H, 5.57; N, 11.91; found: C, 79.01; H, 5.59; N, 11.98.

## Supporting Information

File 1 Additional experimental, photophysical, and calculated data.
