# Supplementary material for: Styryl-based new organic chromophores bearing free amino and azomethine groups: synthesis, photophysical, NLO, and thermal properties
Source: Beilstein J Org Chem. 2020 Sep 14;16:2282–96. doi: 10.3762/bjoc.16.189 (PMC7509381; doi:10.3762/bjoc.16.189)
Supplement: File 1 — Additional experimental, photophysical, and calculated data. [file Beilstein_J_Org_Chem-16-2282-s001.pdf]

BEILSTEIN JOURNAL OF ORGANIC CHEMISTRY

Supporting Information

for

Styryl-based new organic chromophores bearing free amino and azomethine groups: synthesis, photophysical, NLO, and thermal properties

Anka Utama Putra, Deniz Çakmaz, Nurgül Seferoğlu, Alberto Barsella and Zeynel Seferoğlu

Beilstein J. Org. Chem. **2020,** *16,* 2282–2296. doi:10.3762/bjoc.16.189

Additional experimental, photophysical, and calculated data

License and Terms: This is a supporting information file under the terms of the Creative Commons Attribution License (https://creativecommons.org/licenses/by/4.0). Please note that the reuse, redistribution and reproduction in particular requires that the authors and source are credited. The license is subject to the *Beilstein Journal of Organic Chemistry* terms and conditions: (https://www.beilstein-journals.org/bjoc)

Table of Contents

**Contents**	**Page**
1. Characterization data of dyes **4–7** and **9–12**	S3
2. The spectroscopy of FTIR, ^{1}H NMR, ^{13}C NMR and HRMS spectra of dyes **3–12**	S9
2.1. FTIR, ^{1}H NMR, ^{13}C NMR and HRMS spectra of dye **3**	S9
2.2. FTIR, ^{1}H NMR, ^{13}C NMR and HRMS spectra of dye **4**	S11
2.3. FTIR, ^{1}H NMR, ^{13}C NMR and HRMS spectra of dye **5**	S13
2.4. FTIR, ^{1}H NMR, ^{13}C NMR and HRMS spectra of dye **6**	S15
2.5. FTIR, ^{1}H NMR, ^{13}C NMR and HRMS spectra of dye **7**	S17
2.6. FTIR, ^{1}H NMR, ^{13}C NMR and HRMS spectra of dye **8**	S19
2.7.FTIR, ^{1}H NMR, ^{13}C NMR and HRMS spectra of dye **9**	S21
2.8. FTIR, ^{1}H NMR, ^{13}C NMR and HRMS spectra of dye **10**	S23
2.9. FTIR, 1H NMR, ^{13}C NMR and HRMS spectra of dye **11**	S25
2.10.FTIR, ^{1}H NMR, ^{13}C NMR and HRMS spectra of dye **12**	S27
3. Calculated data	S29
3.1. Optimized geometries of dyes **4–7** and **9–12**	S29
3.2. The experimental and calculated photophysical properties of dyes **8–12**	S30
4. Photophysical properties of dyes **3–12**	S31
4.1. Relative quantum yield calculation formula	S31
4.2. Photoluminescence properties of dye **3**	S31
4.3. Photoluminescence properties of dye **4**	S33
4.4. Photoluminescence properties of dye **5**	S34
4.5. Photoluminescence properties of dye **6**	S36
4.6. Photoluminescence properties of dye **7**	S37
4.7. Photoluminescence properties of dye **8**	S39
4.8. Photoluminescence properties of dye **9**	S40
4.9. Photoluminescence properties of dye **10**	S42
4.10. Photoluminescence properties of dye **11**	S43
4.11. Photoluminescence properties of dye **12**	S45
5. Interaction of dyes **8–12** with hydroxide anion	S46
5.1. Interaction of dye **8** with hydroxide anion	S46
5.2. Interaction of dye **9** with hydroxide anion	S47
5.3. Interaction of dye **10** with hydroxide anion	S48
5.4. Interaction of dye **11** with hydroxide anion	S49
5.5. Interaction of dye **12** with hydroxide anion	S50
6. p*K*a calculation and pH properties of dye **8**	S52
7. The Frontier Orbitals of dyes **3–12**	S56
8. The Calculated NLO Properties of dyes **3–12**	S57

1. Characterization data of dyes

((*E*)-2-(1-(4-aminophenyl)-3-(4-(pyrrolidin-1-yl)phenyl)allylidene)malononitrile) (**4**)

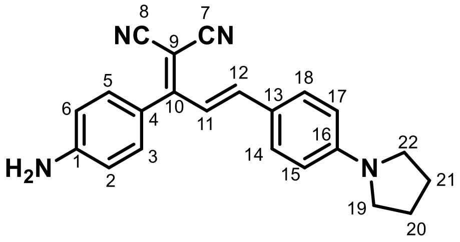

(Yield: 58 %, mp: 199-201 °C, dark green solid) FT-IR (cm^{-1}): 3435, 3343, 3227, 2971, 2860, 2205, 1609, 1569, 1521, 1471. ^{1}H NMR (300 MHz, DMSO-d_6) δ 7.52 (d, *J* = 8.5 Hz, 2H), 7.18 (m, 3H), 6.98 (d, *J* = 15.1 Hz, 1H), 6.67 (d, *J* = 8.3 Hz, 2H), 6.60 (d, *J* = 8.6 Hz, 2H), 6.01 (s, 2H), 3.34 (s, 4H), 1.98 (d, 4H). ^{13}C NMR (75 MHz, DMSO-d_6) δ 171.7 (C10), 153.0 (C1), 150.5 (C16), 150.2 (C3-C5), 132.1 (C12), 131.8 (C11), 122.0 (C13), 120.2 (C4), 118.2 (C14-C18), 116.8 (C15-C17), 116.0 (C2-C6), 113.6 (C7), 112.6 (C8), 70.7 (C9), 47.9 (C19-C22), 25.4 (C20-C21). HR-MS (*m/z*), (M-H)$^{+\cdot}$: $C_{22}H_{20}N_4$ calculated: 341.1766; found: 341.1777. Elemental analysis: anal. calcd. for $C_{22}H_{20}N_4$; C, 77.62; H, 5.92; N, 16.46; found C, 77.58; H, 6.01; N, 16.48.

((*E*)-2-(1-(4-aminophenyl)-3-(4-(piperidin-1-yl)phenyl)allylidene)malononitrile) (**5**)

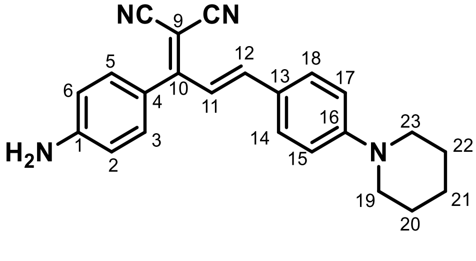

(Yield: 75 %, mp: 193-194 °C, black solid) FT-IR (cm^{-1}): 3460, 3366, 3222, 2933, 2918, 2814, 2204, 1628, 1573, 1545, 1471. ^{1}H NMR (300 MHz, DMSO-d_6) δ 7.50 (m, 2H), 7.20 (m, 3H), 6.96 (m, 3H), 6.66 (m, 2H), 6.05 (s, 2H), 3.39 (s, 4H), 1.57 (s, 6H). ^{13}C NMR (75 MHz, DMSO-d_6) δ 171.7 (C10), 153.5 (C1), 153.4 (C16), 149.1 (C3-C5), 132.3 (C12), 131.2 (C11), 124.8 (C13), 120.6 (C4), 120.0 (C14-C18), 116.5 (C15-C17), 115.7 (C7), 114.5 (C8), 113.6 (C2-C6), 72.4 (C9), 66.3 (C19-C23), 47.3 (C20-C22), 44.6 (C21). HR-MS (*m*/*z*), (M-H)$^{+\cdot}$: $C_{23}H_{22}N_4$ calculated: 355.1923; found: 355.1924. Elemental analysis: anal. calcd. for $C_{23}H_{22}N_4$; C, 77.94; H, 6.26; N, 15.81; found C, 77.88; H, 6.21; N, 15.88.

((*E*)-2-(1-(4-aminophenyl)-3-(4-(morpholin-1-yl)phenyl)allylidene)malononitrile) (**6**)

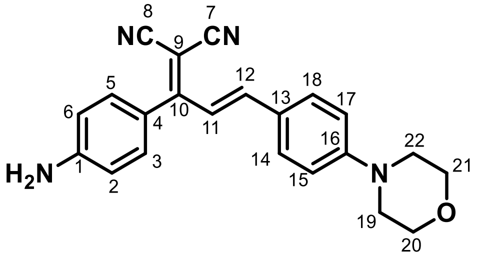

(Yield: 70 %, mp: 168-169 °C, red solid) FT-IR (cm^{-1}): 3467, 3372, 3215, 3033, 2955, 2828, 2210, 1629, 1581, 1512, 1478. ^{1}H NMR (300 MHz, DMSO-d_6) δ 7.57 (d, *J* = 8.6 Hz, 2H), 7.24 (m, 3H), 6.99 (m, 3H), 6.67 (d, *J* = 8.4 Hz, 2H), 6.09 (s, 2H), 3.72 (t, 4H), 3.29 (d, 4H). ^{13}C NMR (75 MHz, DMSO-d_6) δ 171.7 (C10), 153.5 (C1), 153.4 (C16), 149.1 (C3-C5), 132.3 (C12), 131.2 (C11), 124.8 (C13), 120.6 (C4), 120.0 (C14-C18), 116.5 (C15-C17), 115.7 (C7), 114.5 (C8), 113.6 (C2-C6), 72.4 (C9), 66.3 (C20-C21), 47.3 (C19-C22). HR-MS (*m*/*z*), (M-H)$^{+\cdot}$: $C_{22}H_{20}N_4O$ calculated: 357.1715; found: 357.1716. Elemental analysis: anal. calcd. for $C_{22}H_{20}N_4O$; C, 74.14; H, 5.66; N, 15.72; found C, 74.18; H, 5.61; N, 15.81.

((*E*)-2-(1-(4-aminophenyl)-3-(4-(pyridin-1-yl)phenyl)allylidene)malononitrile) (**7**)

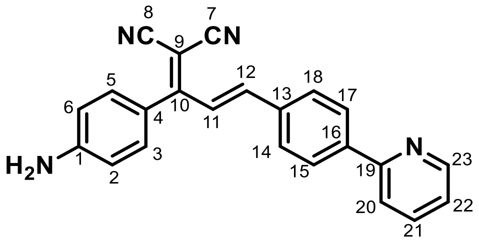

(Yield: 64 %, mp: 214-215 °C, orange solid) FT-IR (cm^{-1}): 3488, 3384, 3221, 3036, 2211, 1597, 1512, 1476, 1463. ^1H NMR (300 MHz, DMSO-d_6) δ 8.70 (m, 1H), 8.19 (d, *J* = 8.3 Hz, 2H), 8.05 (m, 1H), 7.91 (m, 1H), 7.84 (d, *J* = 8.4 Hz, 2H), 7.51 (d, *J* = 15.6 Hz, 1H), 7.37 (m, 3H), 7.17 (d, *J* = 15.6 Hz, 1H), 6.70 (m, 2H), 6.25 (s, 2H). ^{13}C NMR (75 MHz, DMSO-d_6) δ 170.9 (C10), 155.3 (C19), 154.0 (C1), 150.2 (C23), 147.4 (C21), 141.2 (C16), 137.8 (C12), 135.6 (C11), 132.8 (C13), 129.6 (C15-C17), 127.6 (C3-C5), 125.8 (C14-C18), 123.7 (C4), 121.1 (C20), 119.6 (C22), 116.1 (C7), 115.2 (C8), 113.7 (C2-C6), 75.0 (C9). HR-MS (*m/z*), $(M-H)^{+\cdot}$: $C_{23}H_{16}N_4$ calculated: 349.1453; found: 349.1458. Elemental analysis: anal. calcd. for $C_{23}H_{16}N_4$; C, 79.29; H, 4.63; N, 16.08; found C, 79.27; H, 4.60; N, 16.11.

(2-((*E*)-1-(4-(((*E*)-2-hydroxybenzylidene)amino)phenyl)-3-(4-(pyrrolidin-1-yl)phenyl)allylidene)malononitrile) (**9**)

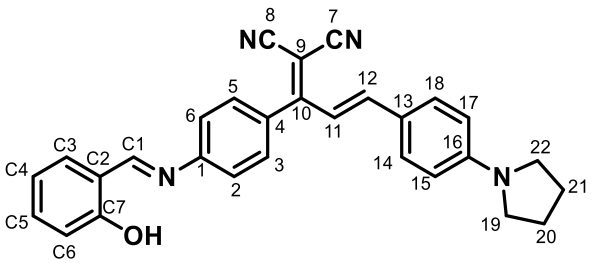

(Yield: 96 %, mp: 234-235 ^{o}C, green solid) FT-IR (cm^{-1}): 3394 (broad), 3035, 2972, 2865, 2206, 1616, 1588, 1523, 1475. ^{1}H NMR (300 MHz, DMSO-d_6) δ 12.81 (s, 1H), 9.06 (s, 1H), 7.71 (d, J = 7.7 Hz, 1H), 7.53 (m, 7H), 7.26 (d, J = 15.0 Hz, 1H), 7.01 (t, J = 8.0 Hz, 2H), 6.89 (d, J = 15.1 Hz, 1H), 6.60 (d, J = 8.5 Hz, 2H), 3.35 (s, 4H), 1.96 (s, 4H). ^{13}C NMR (75 MHz, DMSO-d_6) δ 192.2, 165.2 (C10), 161.2 (C1'), 160.8 (C7'), 153.0 (C1), 150.5 (C16), 150.2 (C3-C5), 136.9 (C5'), 133.1 (C3'), 132.1 (C12), 131.8 (C11), 130.9, 129.7, 122.3 (C2'), 122.0 (C4'), 120.3 (C13), 119.9 (C4), 118.2 (C14-C18), 117.7, 117.2 C15-C17, 116.8 (C2-C6), 113.6 (C7), 112.8 (C8), 112.6 (C6'), 70.7 (C9), 47.9 (C19-C22), 25.3 (C20-C21). HR-MS (m/z), (M-H)$^{+\cdot}$: $C_{29}H_{24}N_4O$ calculated: 453.1715; found: 453.1715. Elemental analysis: anal. calcd. for $C_{29}H_{24}N_4O$; C, 78.36; H, 5.44; N, 12.60; found C, 78.27; H, 5.50; N, 12.61.

(2-((*E*)-1-(4-(((*E*)-2-hydroxybenzylidene)amino)phenyl)-3-(4-(piperidin-1-yl)phenyl)allylidene)malononitrile) (**10**)

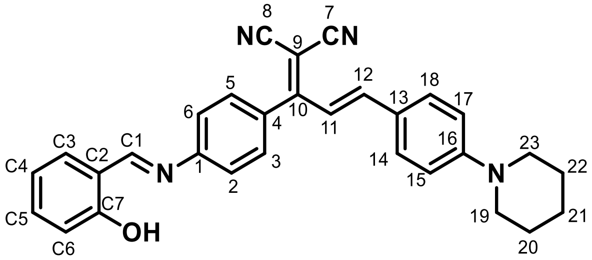

(Yield: 95 %, mp: 212-213 ^{o}C, black solid) FT-IR (cm^{-1}): 3319 (broad), 3043, 2925, 2846, 2212, 1620, 1574, 1515, 1464. ^{1}H NMR (300 MHz, DMSO-d_6) δ 12.80 (s, 1H), 9.06 (d, 1H), 7.71 (d, J = 7.5 Hz, 1H), 7.54 (m, 8H), 7.30 (dd, 1H), 6.95 (m, 4H), 3.41 (t, 4H), 1.57 (s, 6H). ^{13}C NMR (75 MHz, DMSO-d_6) δ 171.0 (C10), 165.2 (C1'), 160.8 (C7'), 153.6 (C1), 150.8 (C16), 150.3 (C3-C5), 136.9 (C5'), 134.2 (C3'), 133.1 (C12), 132.3 (C11), 132.1 (C13), 131.0 (C2'), 123.5 (C4'), 123.0 (C4), 122.3 (C14-C18), 119.8 (C15-C17), 117.2 (C7), 115.4 (C8), 114.6 (C2-C6), 114.4 (C6'), 75.8 (C9), 48.1 (C19-C23), 25.4 (C20-C22), 24.4 (C21). HR-MS (m/z), $(M-H)^{+\cdot}$: $C_{30}H_{26}N_4O$ calculated: 459,2185; found: 459.2175. Elemental analysis: anal. calcd. for $C_{30}H_{26}N_4O$; C, 78.58; H, 5.72; N, 12.22; found C, 78.57; H, 5.70; N, 12.28.

(2-((*E*)-1-(4-(((*E*)-2-hydroxybenzylidene)amino)phenyl)-3-(4-(morpholin-1-yl)phenyl)allylidene)malononitrile) (**11**)

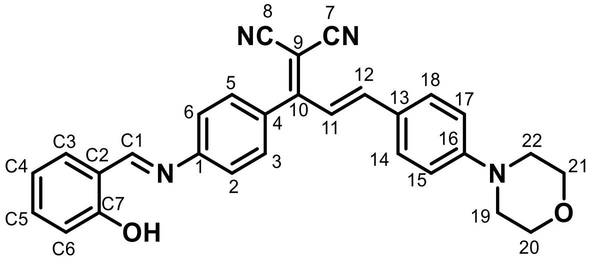

(Yield: 93 %, mp: 187-188 ^{o}C, red solid) FT-IR (cm^{-1}): 3404 (broad), 3094, 2969, 2848, 2217, 2207, 1615, 1586, 1516, 1489. ^{1}H NMR (300 MHz, DMSO-d_6) δ 12.79 (s, 1H), 9.07 (s, 1H), 7.71 (dd, 1H), 7.59 (q, 6H), 7.46 (m, 1H), 7.36 (d, J = 15.3 Hz, 1H), 6.97 (m, 5H), 3.71 (t, 4H), 3.31 (t, 4H). ^{13}C NMR (75 MHz, DMSO-d_6) δ 192.2, 171.1 (C10), 165.2 (C1'), 160.8 (C7'), 153.8 (C1), 150.9 (C16), 150.1 (C3-C5), 136.9 (C5'), 134.2 (C3'), 133.1 (C12), 132.3, 132.1 (C11), 131.8 (C), 131.2 (C13), 131.0 (C4'), 129.6 (C6'), 124.4 (C4),

122.3 (C14-C18), 119.9 (C15-C17), 117.7 (C7), 117.2 (C8), 114.4 (C), 113.6 (C2-C6), 77.1 (C9), 66.3 (C20-C21), 47.3 (C19-C22). HR-MS (*m*/*z*), $(M\text{-}H)^{+\cdot}$: $C_{29}H_{24}N_4O_2$ calculated: 461.1978; found: 461.1961. Elemental analysis: anal. calcd. for $C_{29}H_{24}N_4O_2$; C, 75.63; H, 5.25; N, 12.17; found C, 75.71; H, 5.30; N, 12.22.

(2-((*E*)-1-(4-(((*E*)-2-hydroxybenzylidene)amino)phenyl)-3-(4-(pyridin-1-yl)phenyl)allylidene)malononitrile) (**12**)

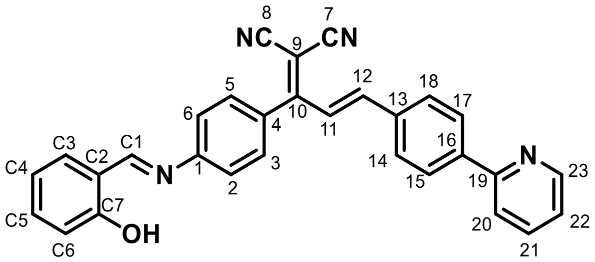

(Yield: 59 %, mp: 205-206 ºC, orange solid) FT-IR (cm^{-1}): 3600-3000 (broad), 3048, 2217, 1620, 1594, 1568, 1488, 1453. ^{1}H NMR (300 MHz, DMSO-d_6) δ 12.77 (s, 1H), 9.08 (s, 1H), 8.70 (m, 1H), 8.21 (d, J = 8.2 Hz, 2H), 8.06 (d, J = 8.0 Hz, 1H), 7.90 (m, 3H), 7.73 (m, 1H), 7.66 (m, 5H), 7.48 (m, 1H), 7.41 (m, 1H), 7.12 (s, 1H), 7.03 (m, 3H). ^{13}C NMR (75 MHz, DMSO-d_6) δ 192.2, 191.6, 165.3 (C10), 160.8 (C1’), 155.2 (C7’), 154.0 (C19), 150.2 (C1), 149.6 (C23), 137.9 (C21), 136.9 (C5’), 133.1 (C21), 132.8 (C16), 131.2 (C3’), 130.7 (C12), 130.0 (C11), 129.6 (C13), 129.4 (C15-C17), 129.1 (C3-C5), 127.6 (C2’), 127.6 (C14-C18), 127.0 (C4), 122.5 (C20), 119.9 (C4’), 119.8 (C22), 119.4 (C6’), 117.7 (C7), 117.2 (C8), 113.7 (C2-C6), 113.1, 95.7 (C9). HR-MS (*m*/*z*), $(M\text{-}H)^{+\cdot}$: $C_{30}H_{20}N_4O$ calculated: 453.1715; found: 453.1715. Elemental analysis: anal. calcd. for $C_{30}H_{20}N_4O$; C, 79.63; H, 4.46; N, 12.38; found C, 79.74; H, 4.49; N, 12.42.

2. The spectroscopy of FTIR, ^{1}H NMR, ^{13}C NMR and HRMS spectra of dyes 3-12

2.1. FTIR, ^{1}H NMR, ^{13}C NMR and HRMS spectra of dye 3

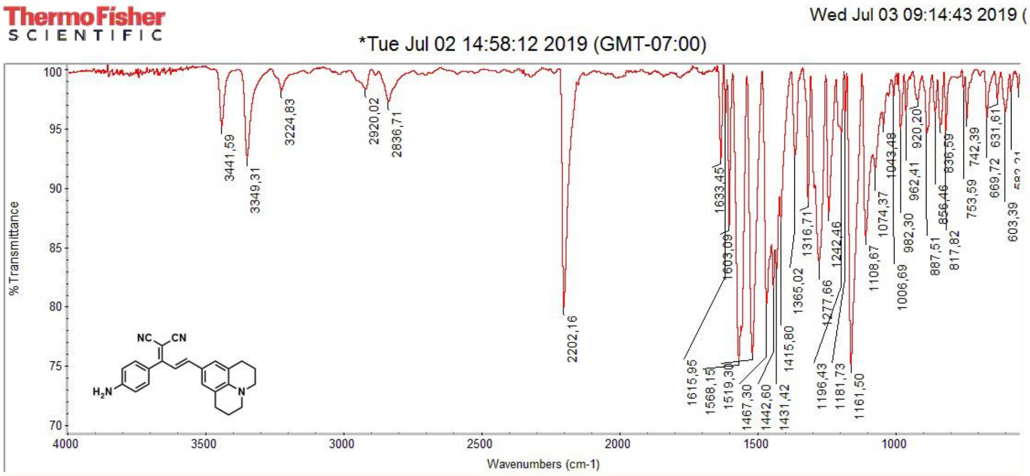

Figure S1: FTIR spectrum of dye **3**.

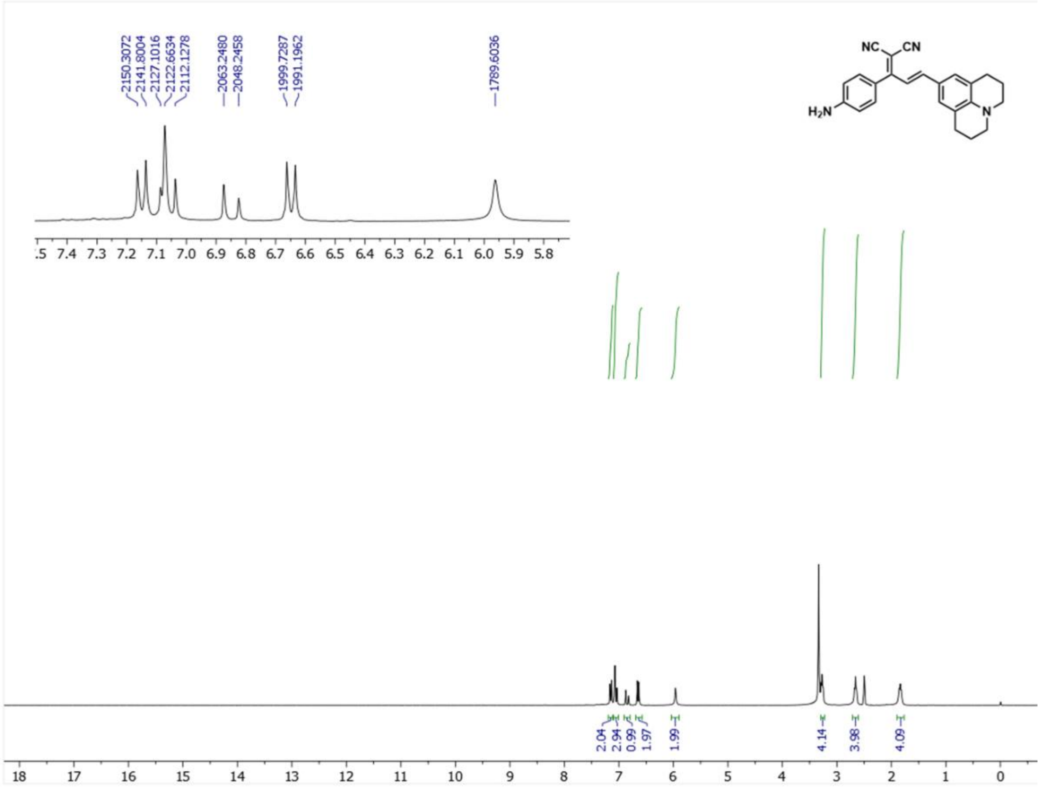

Figure S2: ^{1}H NMR spectrum of dye **3** in DMSO-d_6.

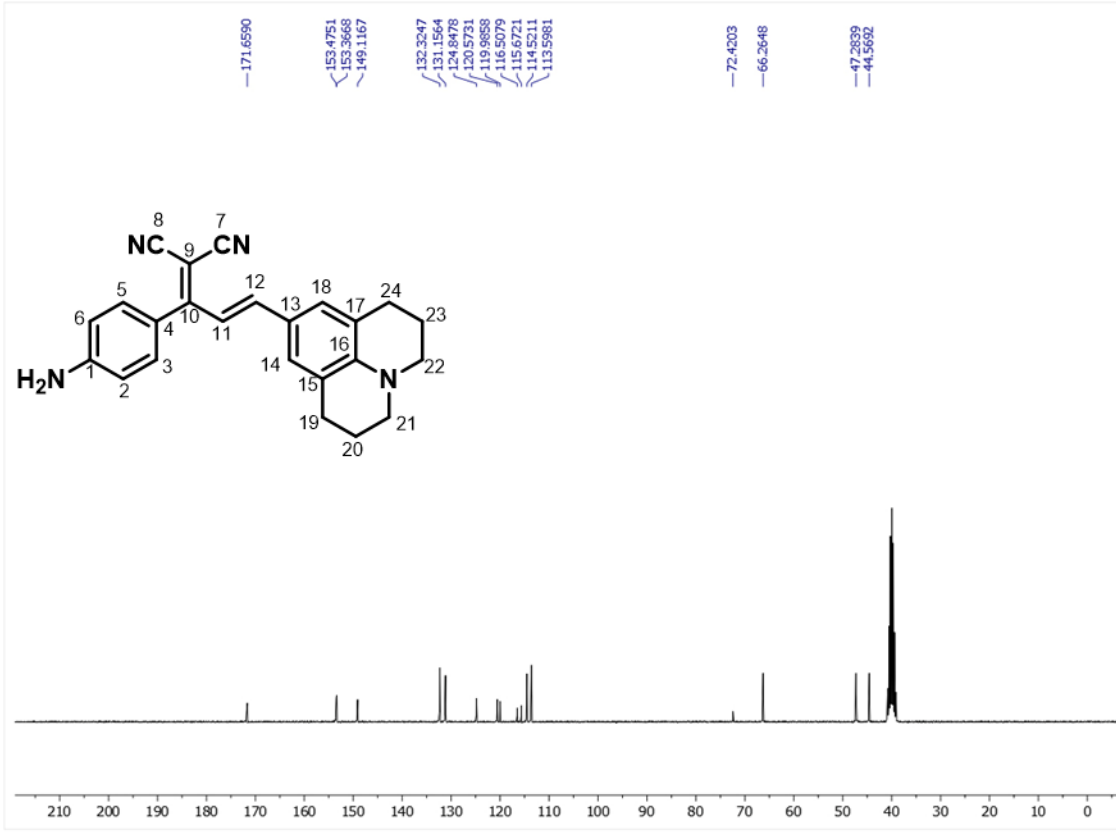

Figure S3: ^{13}C NMR spectrum of dye **3** in DMSO-d_6.

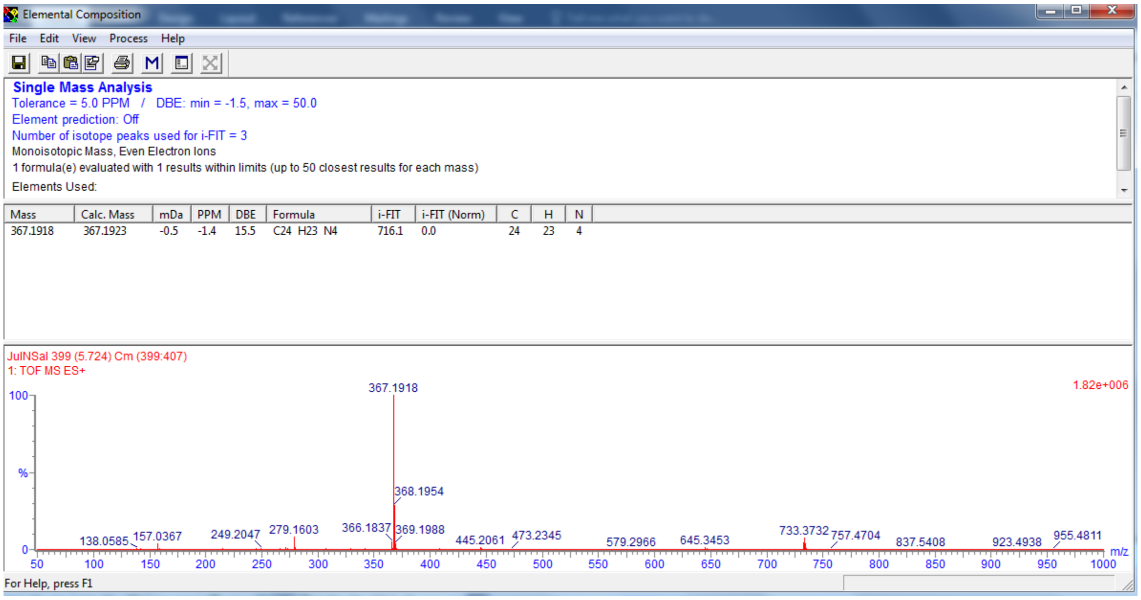

Figure S4: HRMS spectrum of dye **3**.

2.2. FTIR, ^{1}H NMR, ^{13}C NMR and HRMS spectra of dye 4

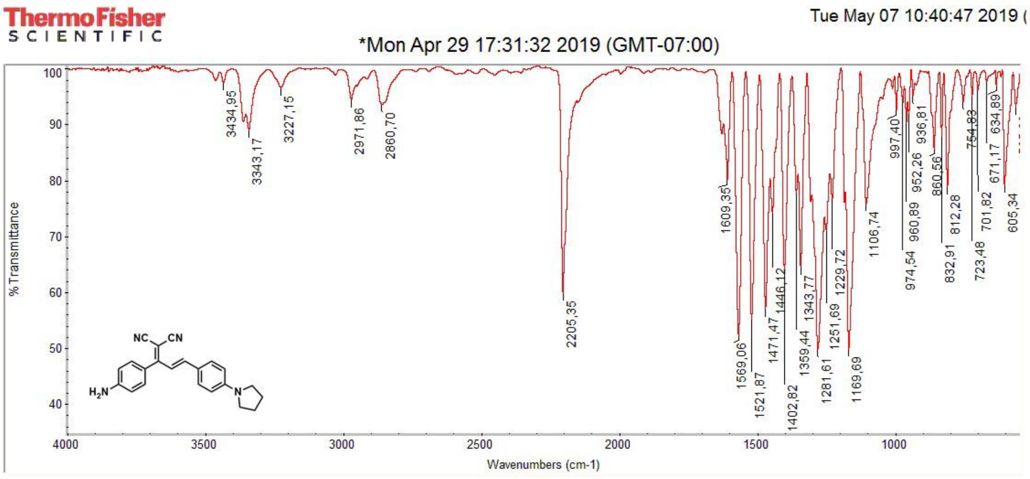

Figure S5: FTIR spectrum of dye **4**.

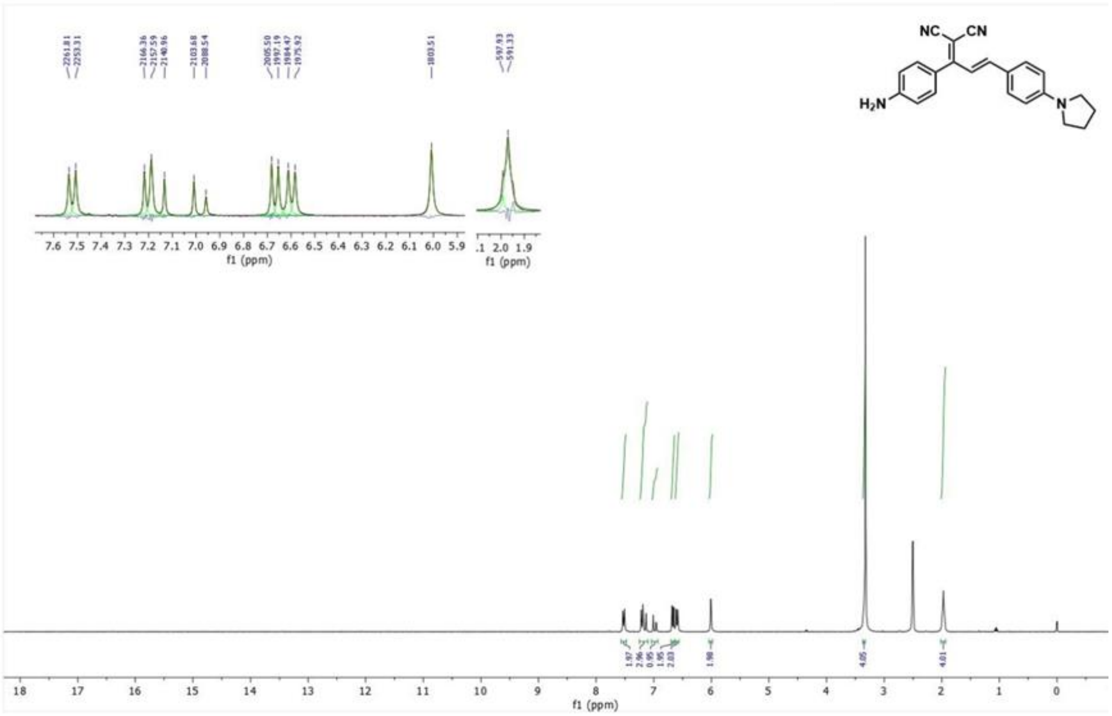

Figure S6: ^{1}H NMR spectrum dye **4** in DMSO-d_6.

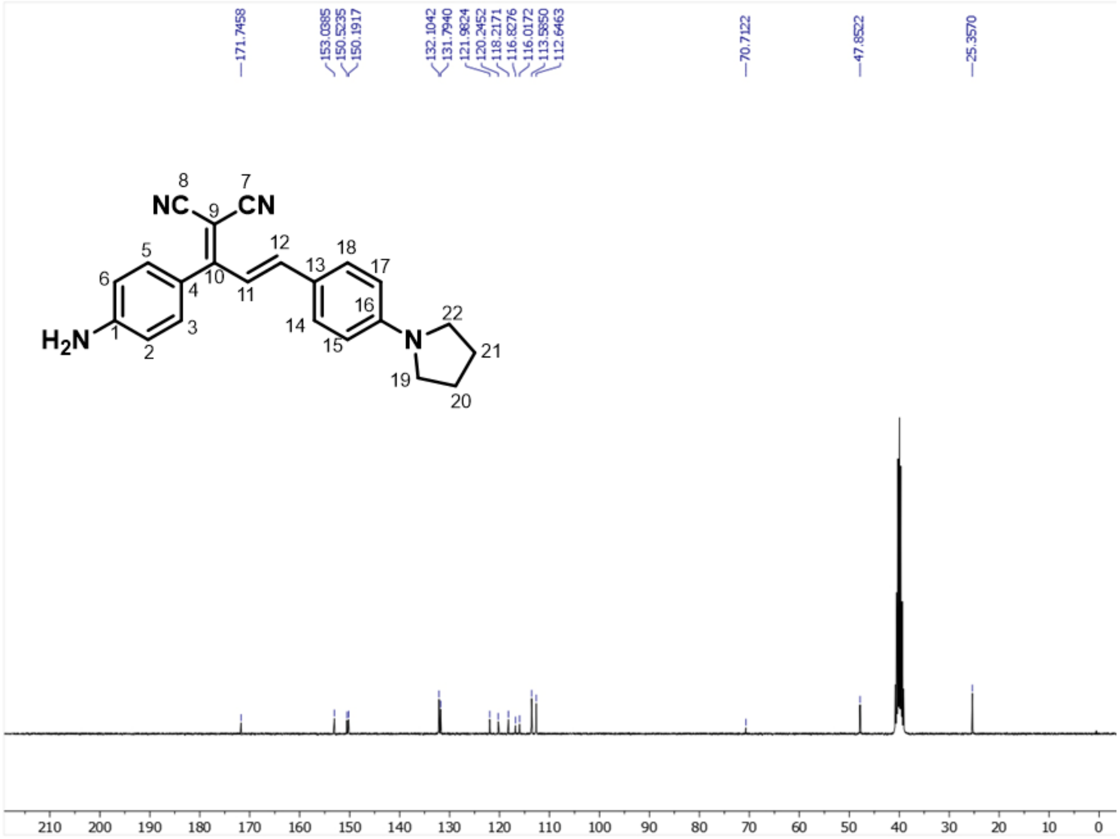

Figure S7: ^{13}C NMR spectrum of dye **4** in DMSO-d_6.

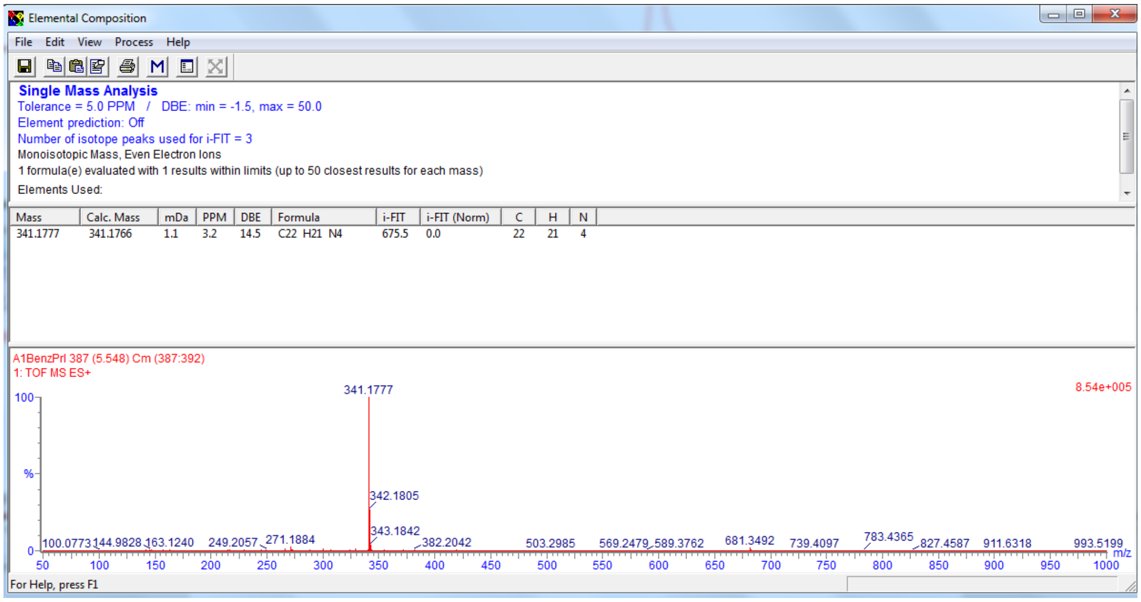

Figure S8: HRMS spectrum of dye **4**.

2.3. FTIR, ^{1}H NMR, ^{13}C NMR and HRMS spectra of dye 5

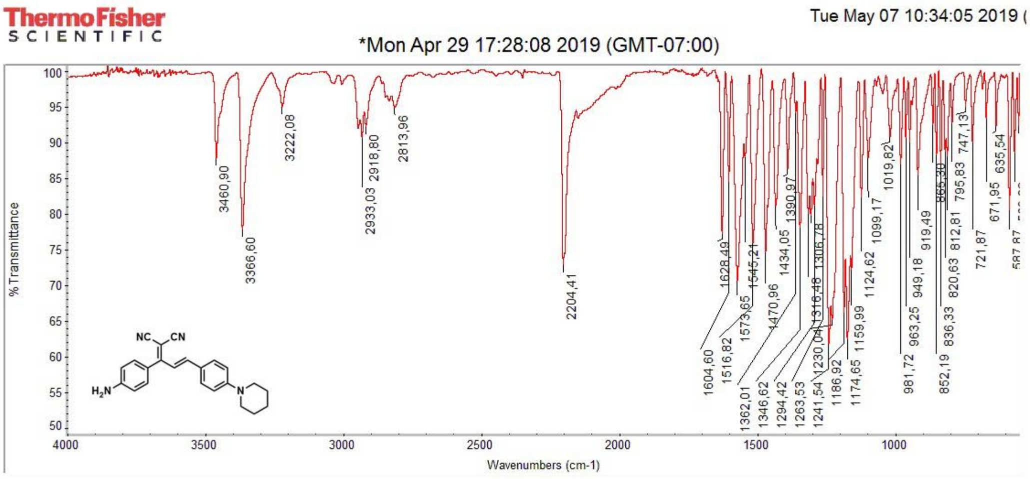

Figure S9: FTIR spectrum of dye **5**.

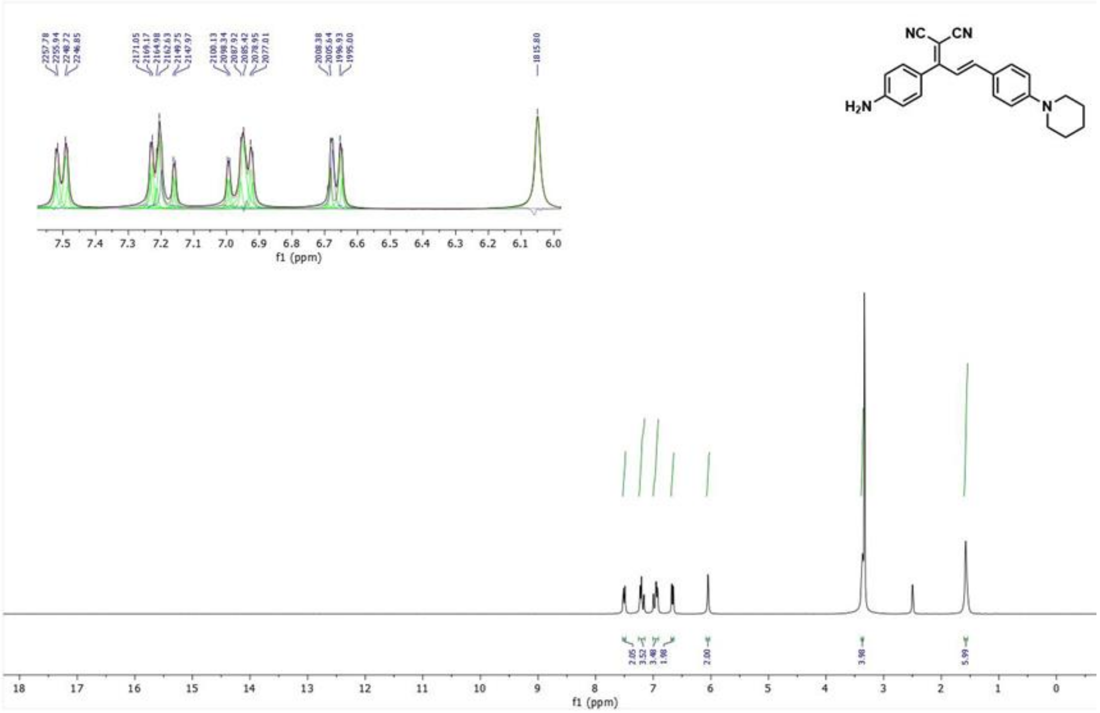

Figure S10: ^{1}H NMR spectrum of dye **5** in DMSO-d_6.

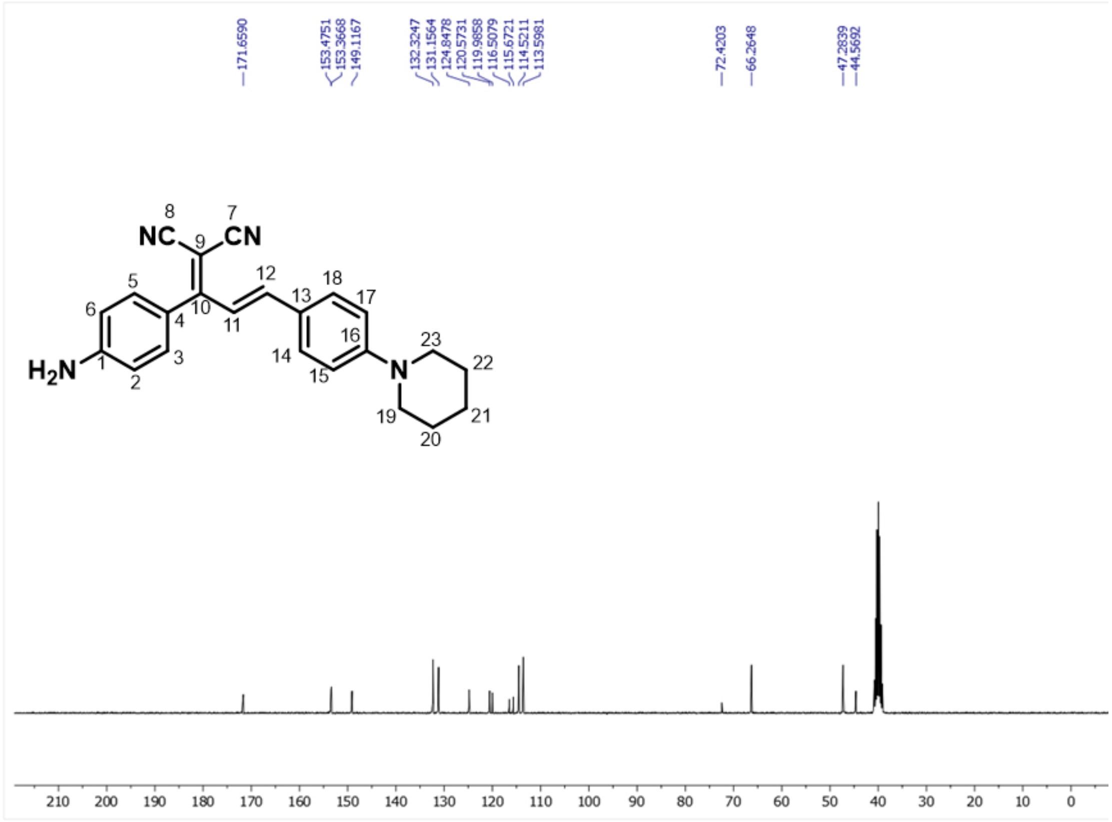

Figure S11: ^{13}C NMR spectrum of dye **5** in DMSO-d_6.

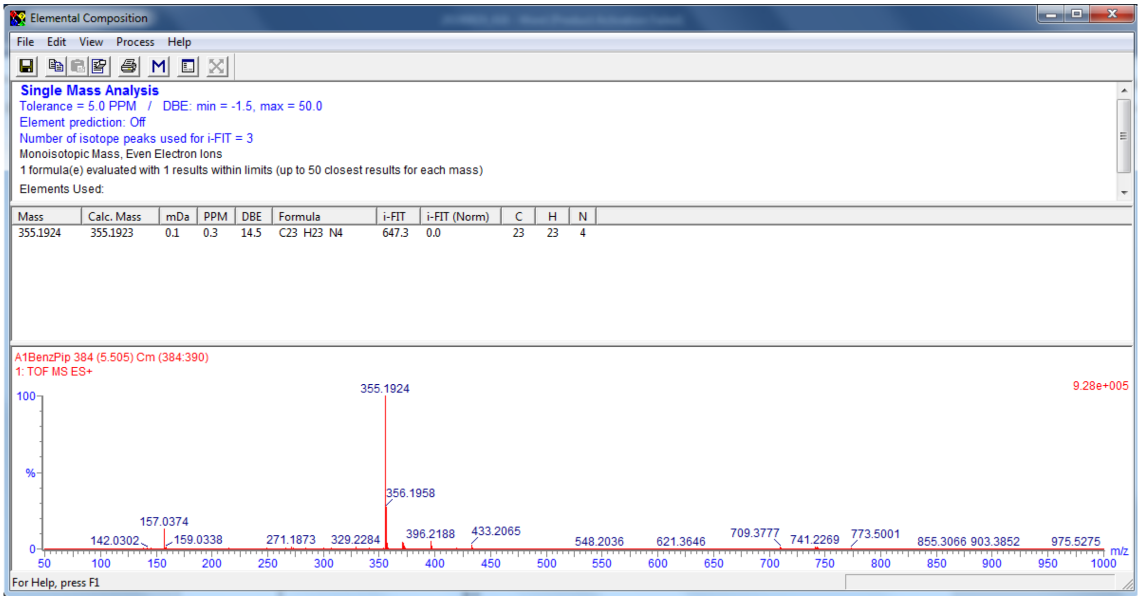

Figure S12: HRMS spectrum of dye **5**.

2.4. FTIR, ^1H NMR, ^{13}C NMR and HRMS spectra of dye 6

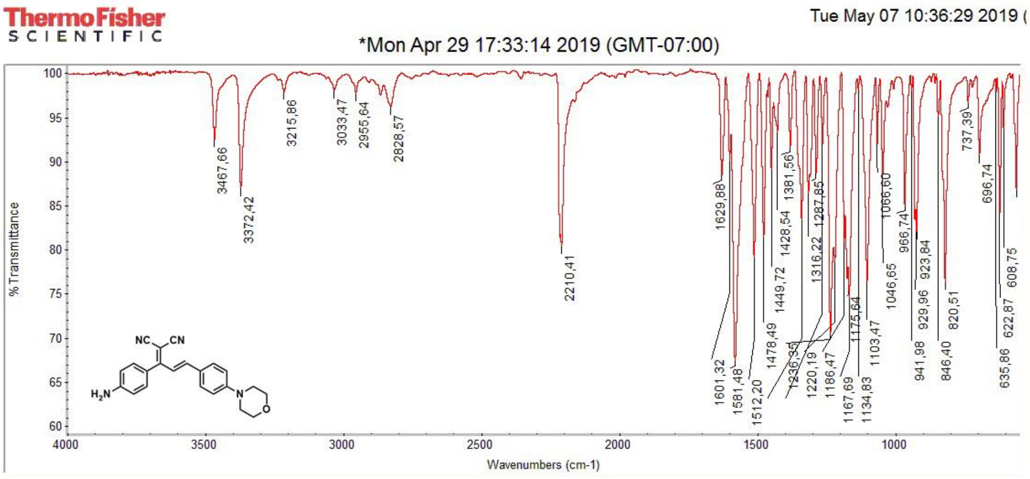

Figure S13: FTIR spectrum of dye **6**.

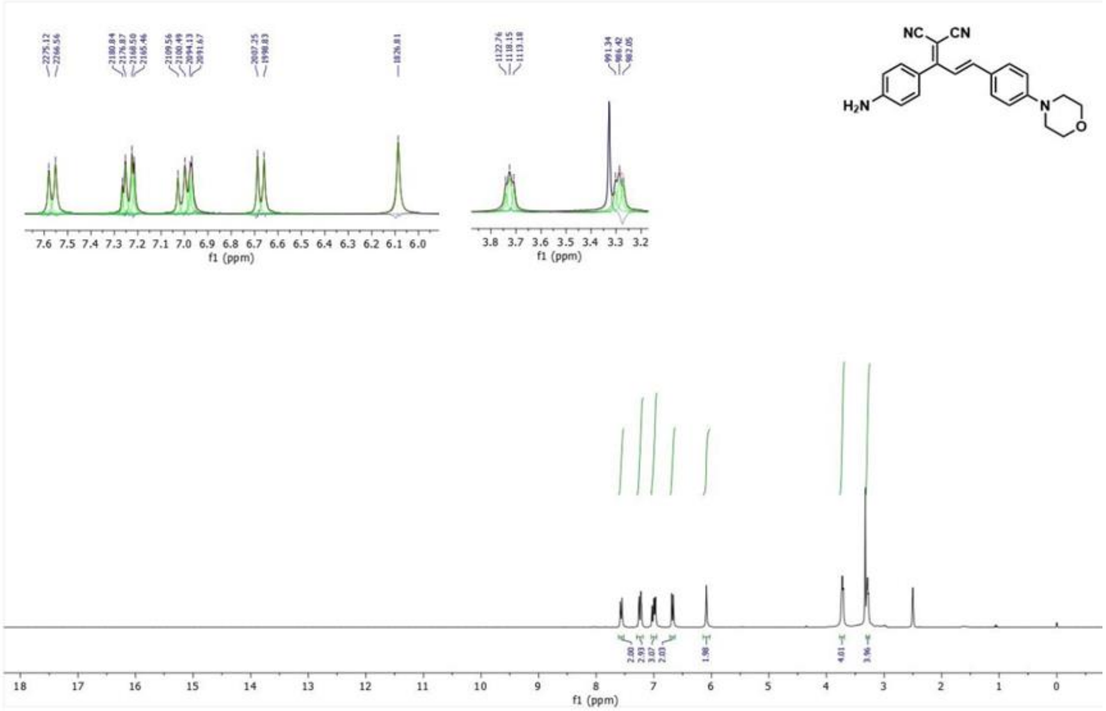

Figure S14: ^1H NMR spectrum of dye **6** in DMSO-d_6.

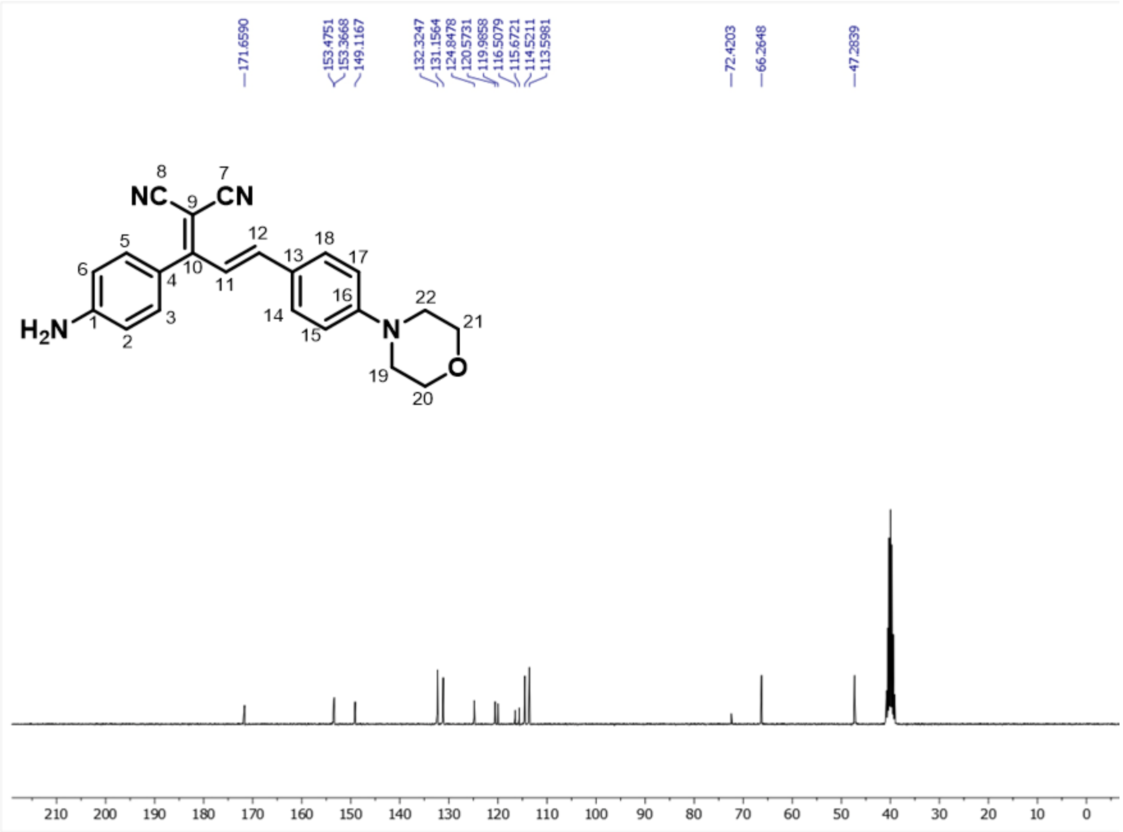

Figure S15: ^{13}C NMR spectrum of dye **6** in DMSO-d_6.

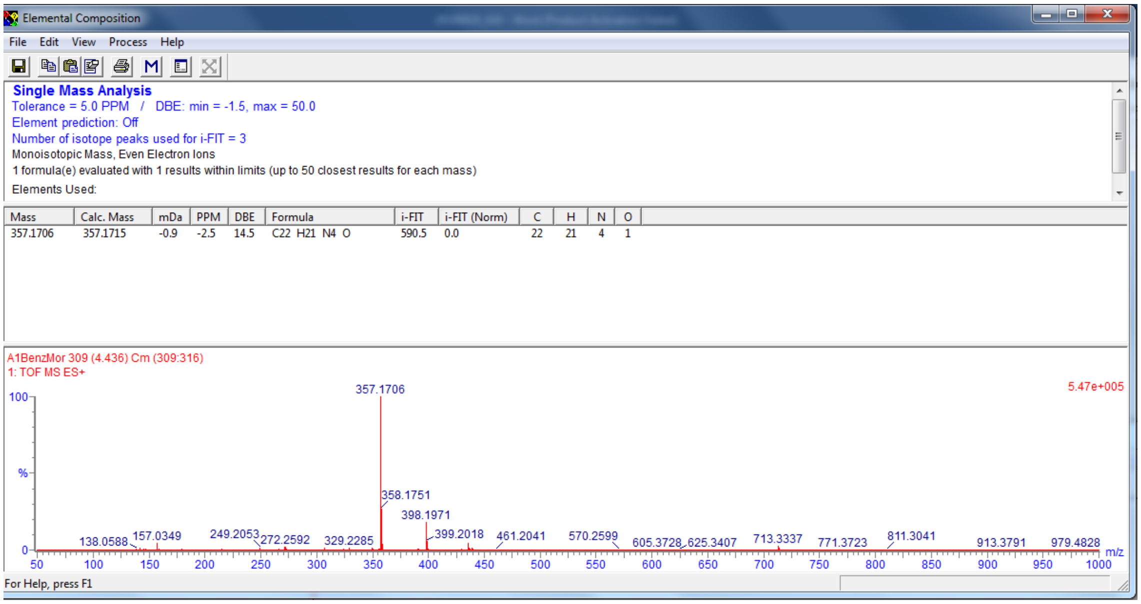

Figure S16: HRMS spectrum of dye **6**.

2.5. FTIR, ^{1}H NMR, ^{13}C NMR and HRMS spectra of dye 7

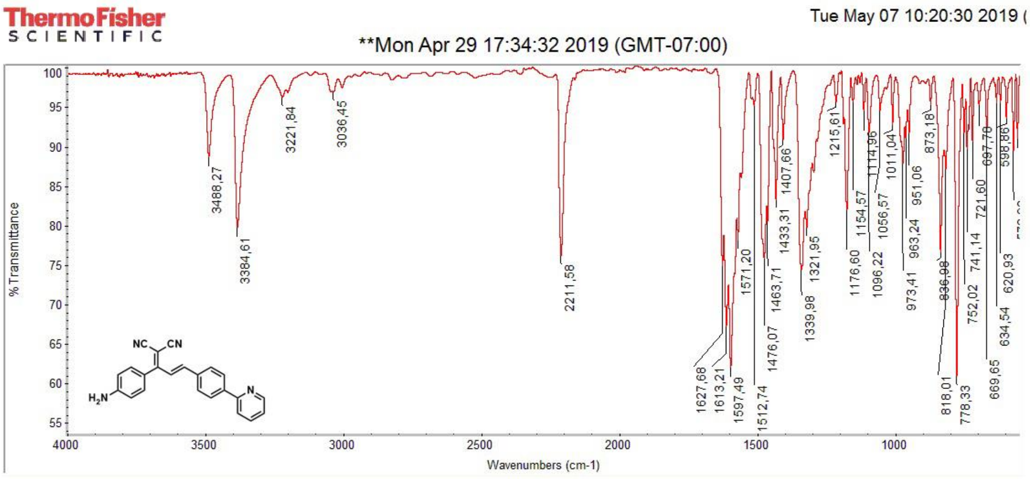

Figure S17: FTIR spectrum of dye **7**.

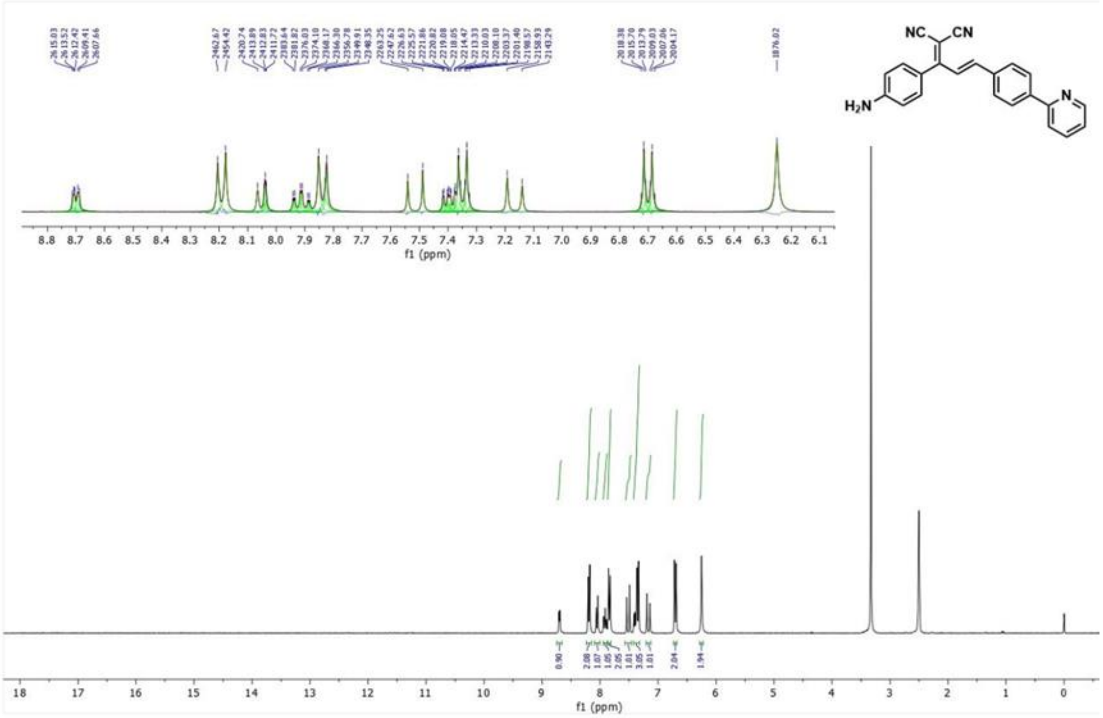

Figure S18: ^{1}H NMR spectrum of dye **7** in DMSO-d_6.

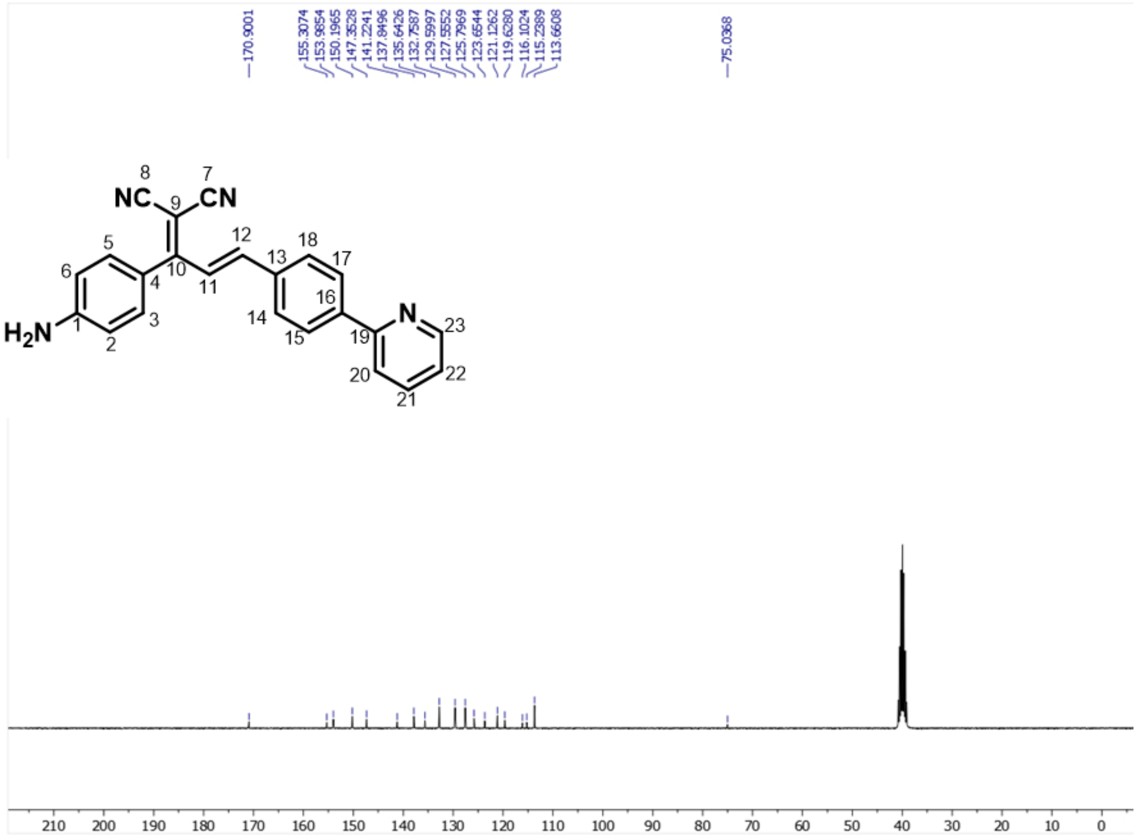

Figure S19: ^{13}C NMR spectrum of dye **7** in DMSO-d_6.

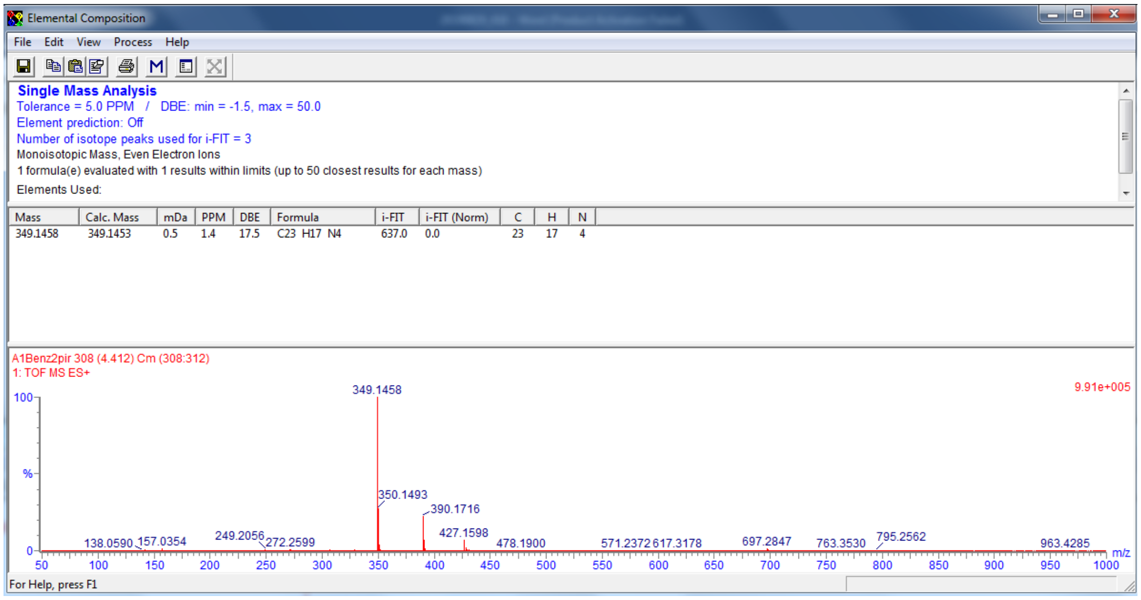

Figure S20: HRMS spectrum of dye **7**.

2.6. FTIR, ^{1}H NMR, ^{13}C NMR and HRMS spectra of dye 8

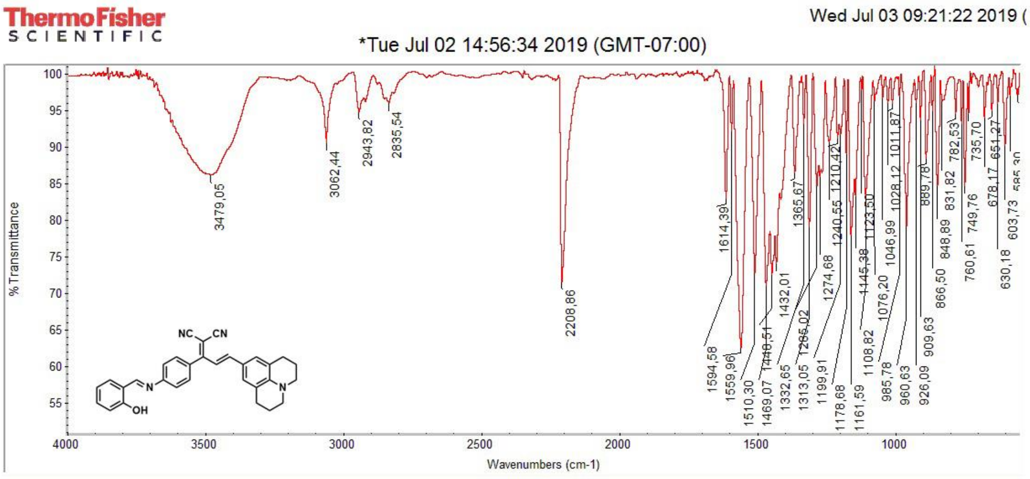

Figure S21: FTIR spectrum of dye **8**.

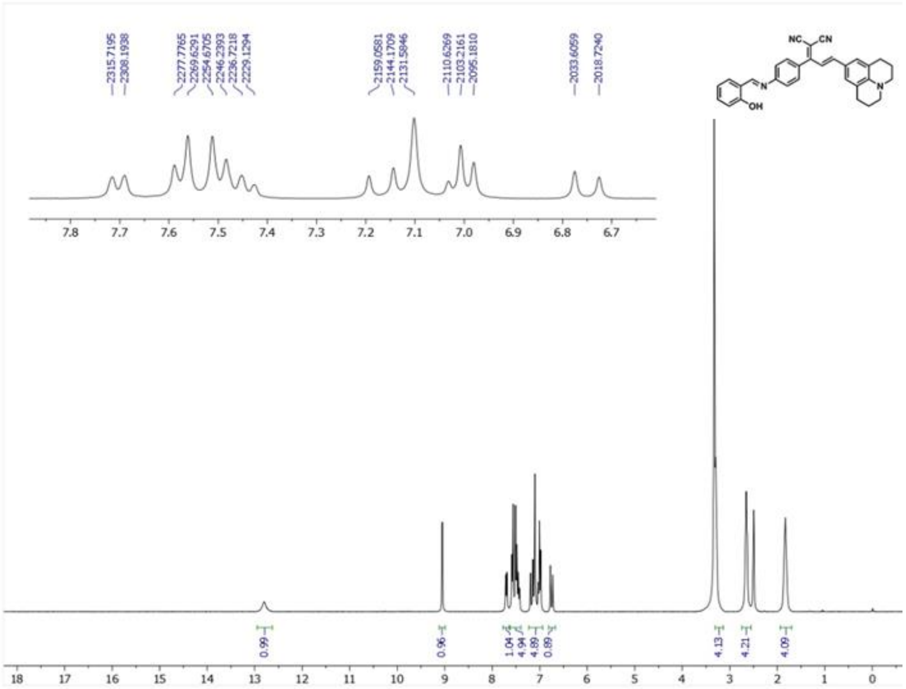

Figure S22: ^{1}H NMR spectrum of dye **8** in DMSO-d_6.

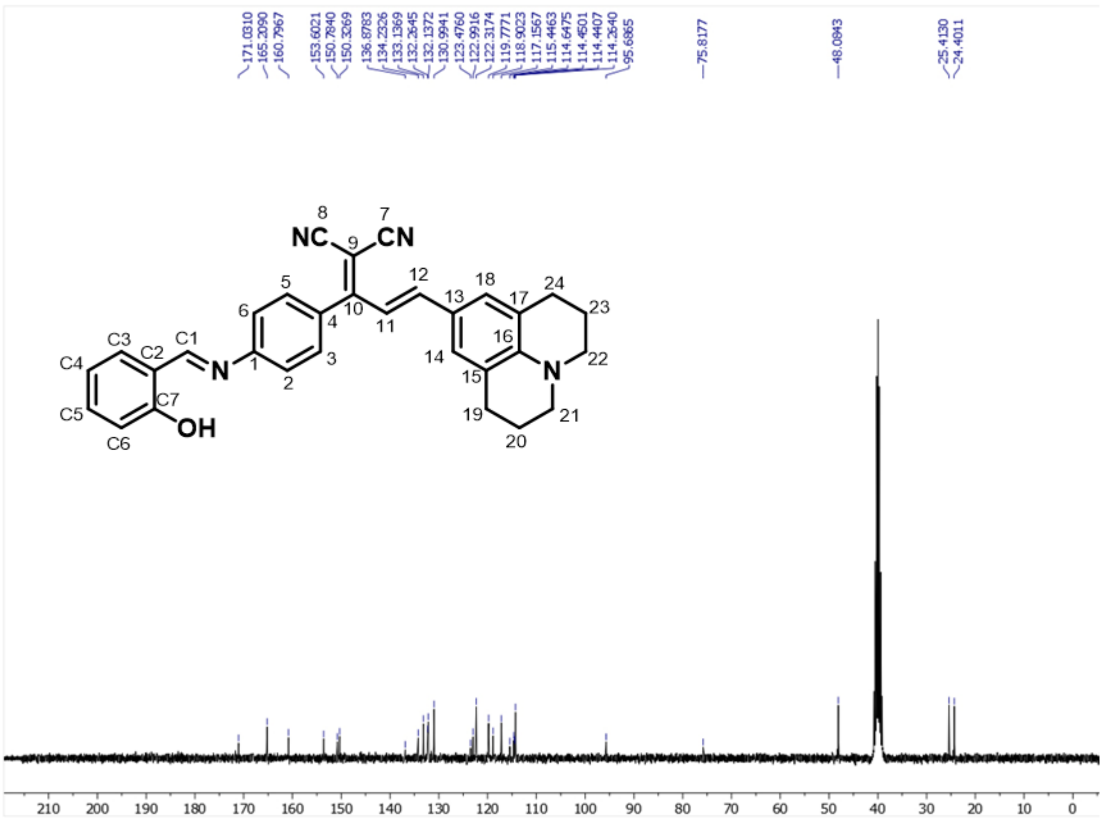

Figure S23: ^{13}C NMR spectrum of dye **8** in DMSO-d_6.

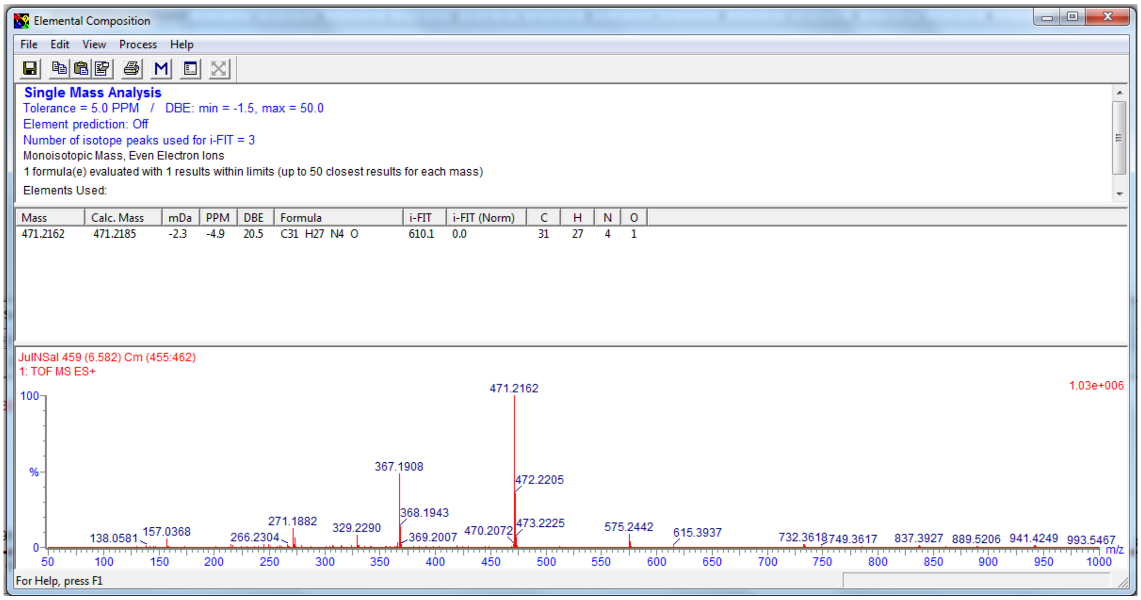

Figure S24: HRMS spectrum of dye **8**.

2.7. FTIR, ^{1}H NMR, ^{13}C NMR and HRMS spectra of dye 9

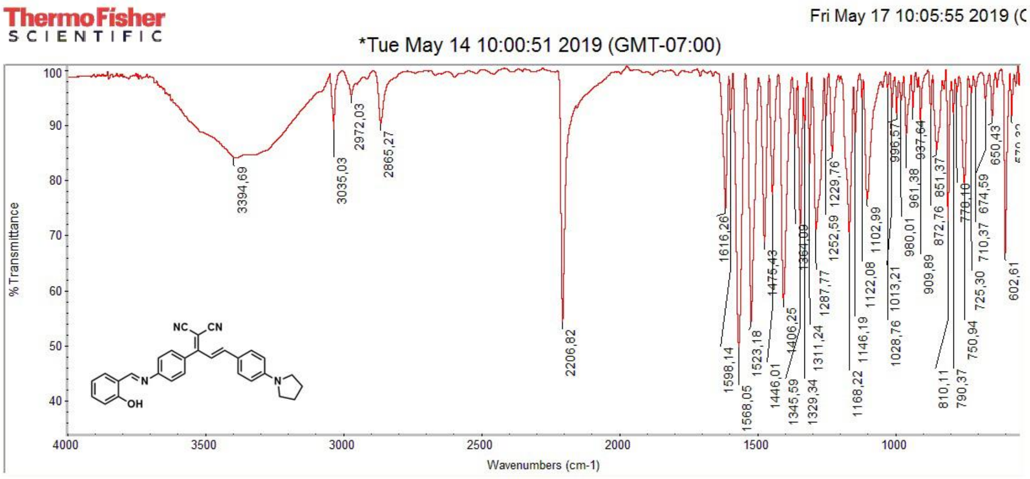

Figure S25: FTIR spectrum of dye **9**.

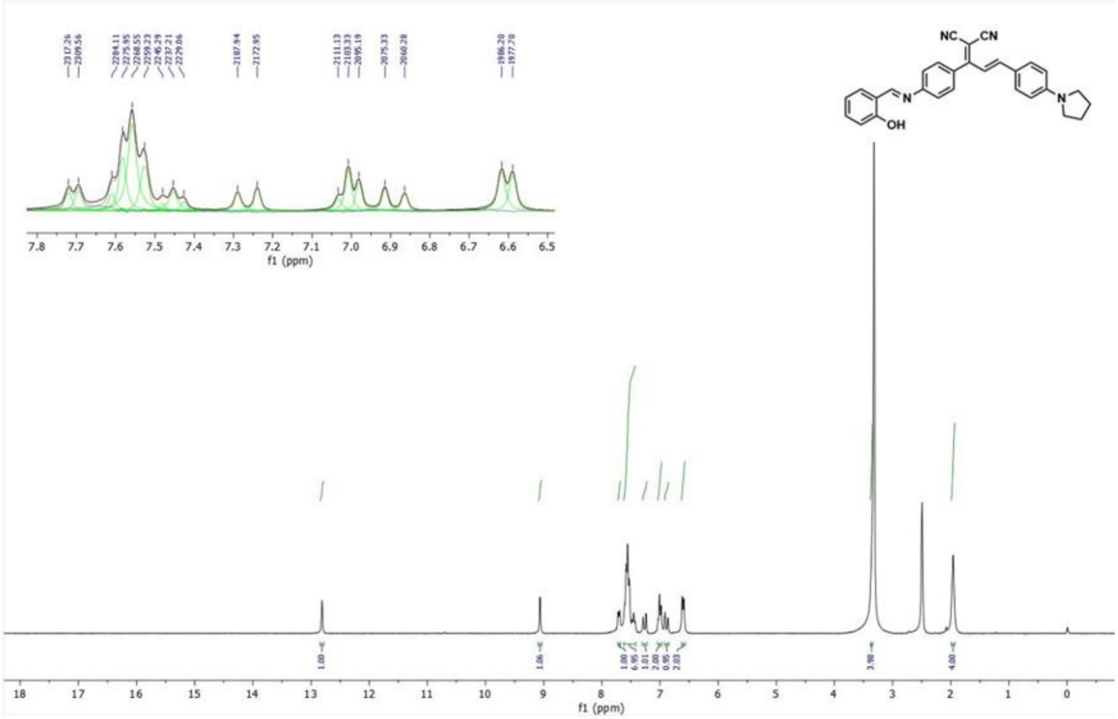

Figure S26: ^{1}H NMR spectrum of dye **9** in DMSO-d_6.

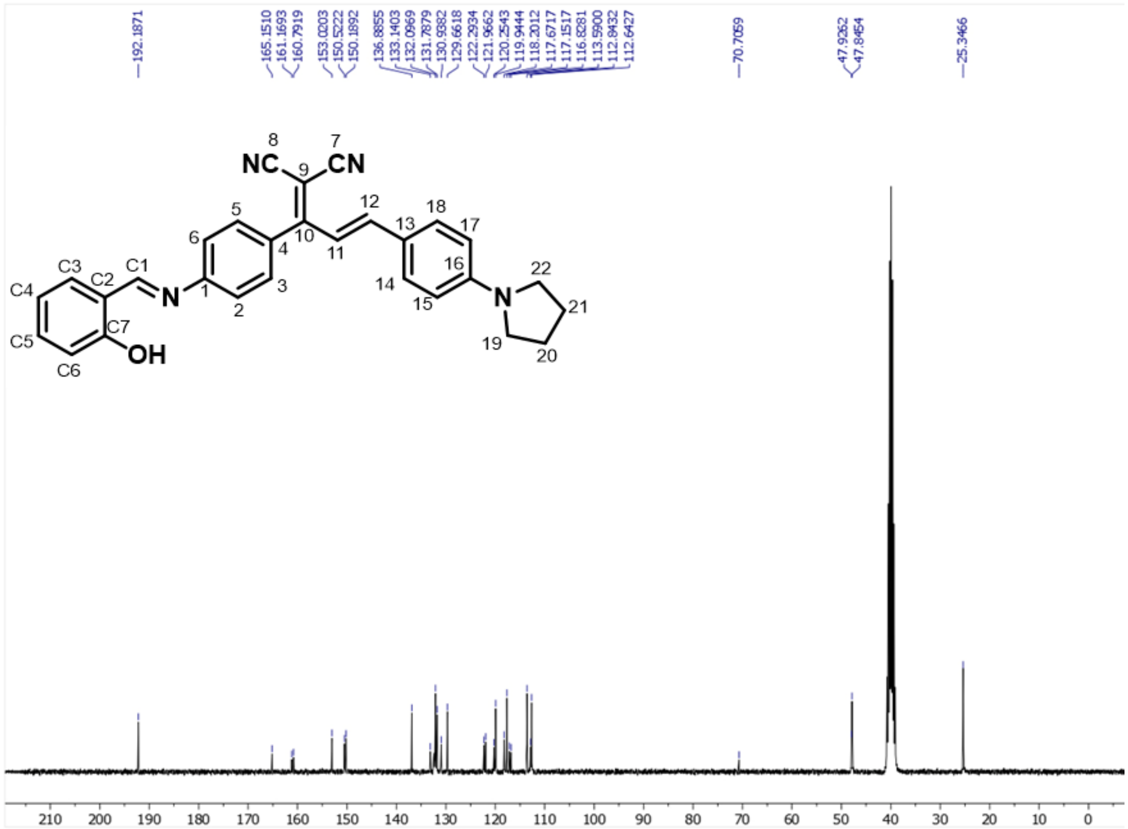

Figure S27: ^{13}C NMR spectrum of dye **9** in DMSO-d_6.

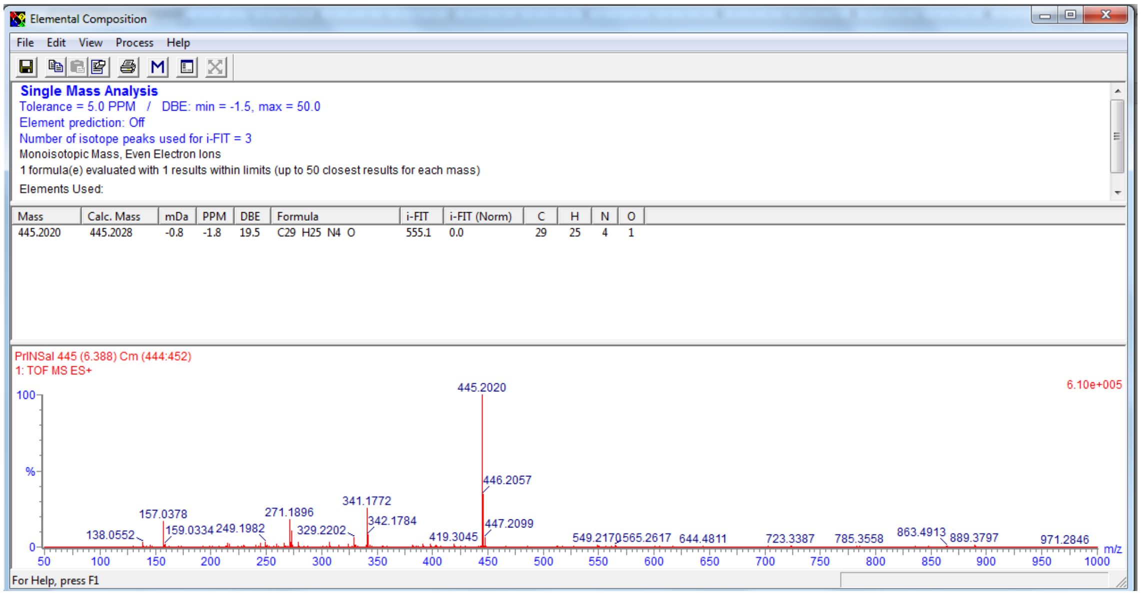

Figure S28: HRMS spectrum of dye **9**.

2.8. FTIR, ^{1}H NMR, ^{13}C NMR and HRMS spectra of dye 10

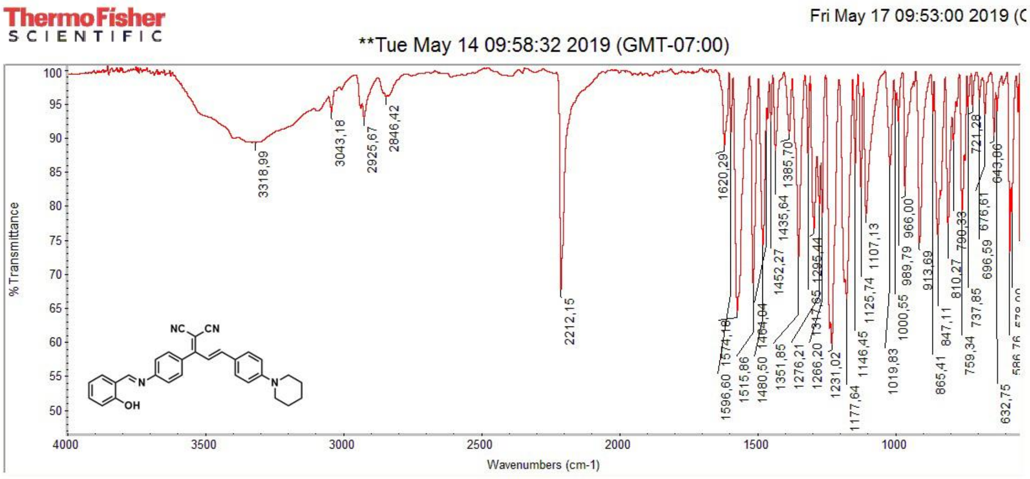

Figure S29: FTIR spectrum of dye **10**.

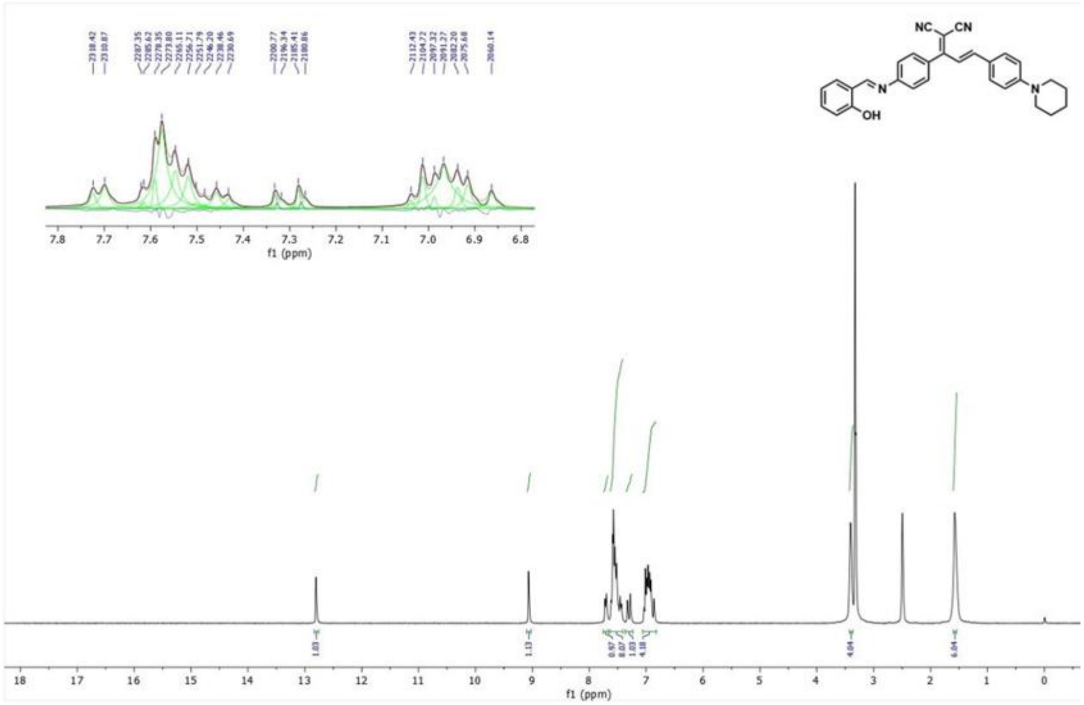

Figure S30: ^{1}H NMR spectrum of dye **10** in DMSO-d_6.

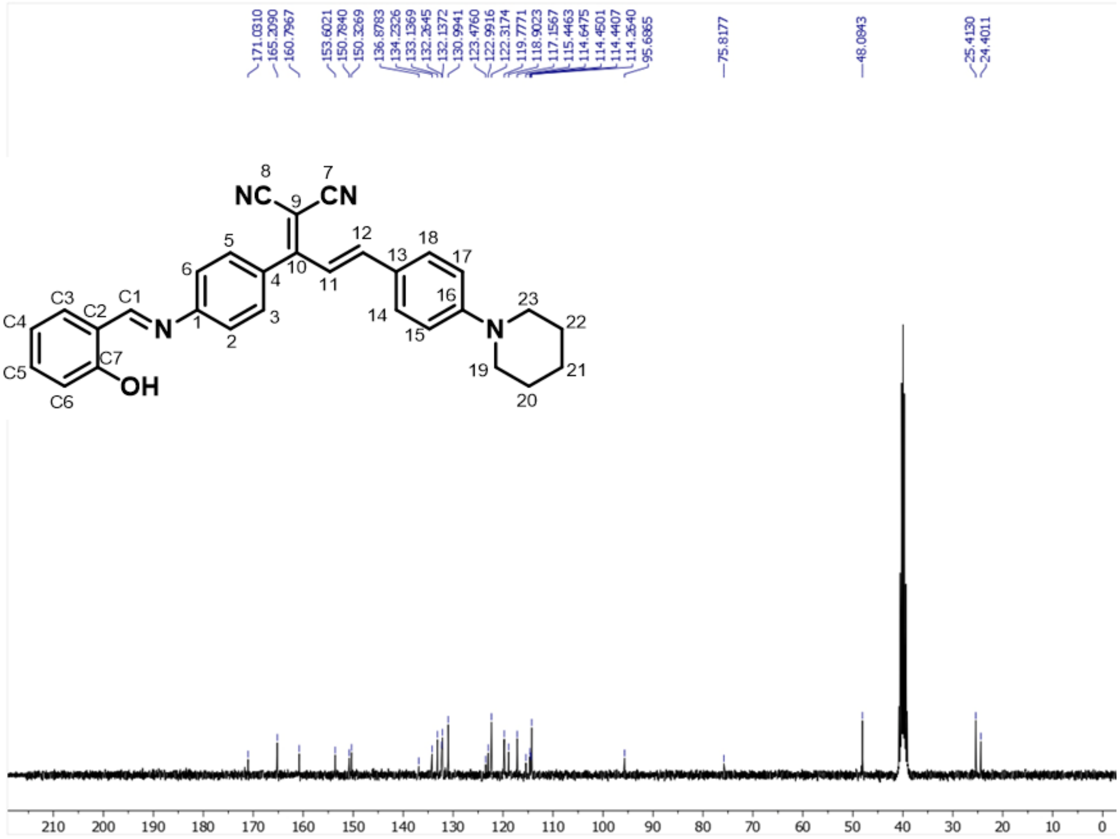

Figure S31: ^{13}C NMR spectrum of dye **10** in DMSO-d_6.

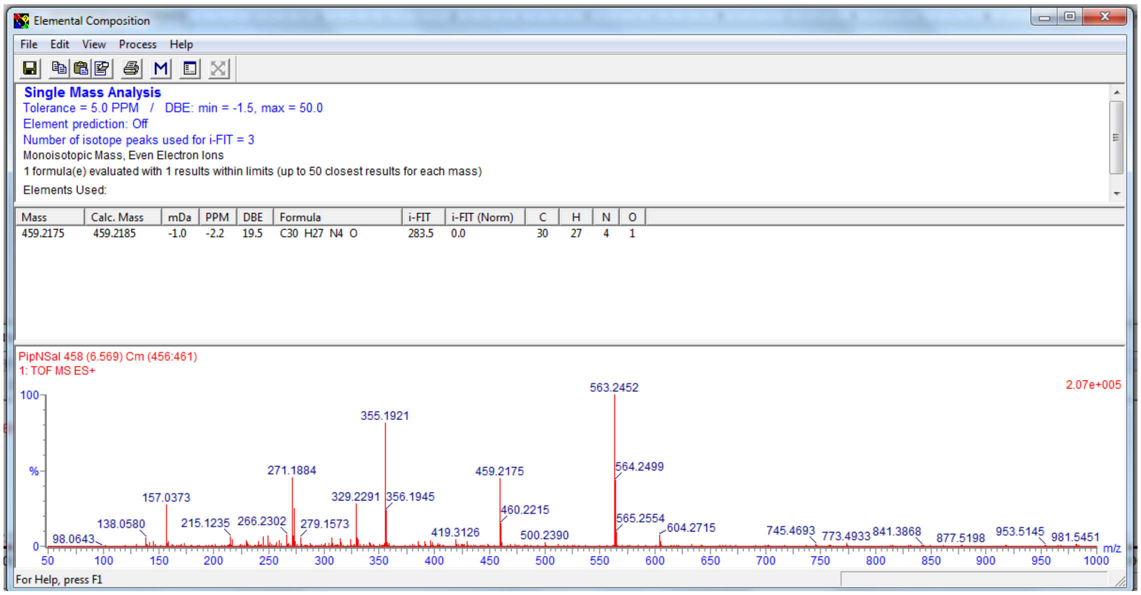

Figure S32: HRMS spectrum of dye **10**.

2.9. FTIR, ^{1}H NMR, ^{13}C NMR and HRMS spectra of dye 11

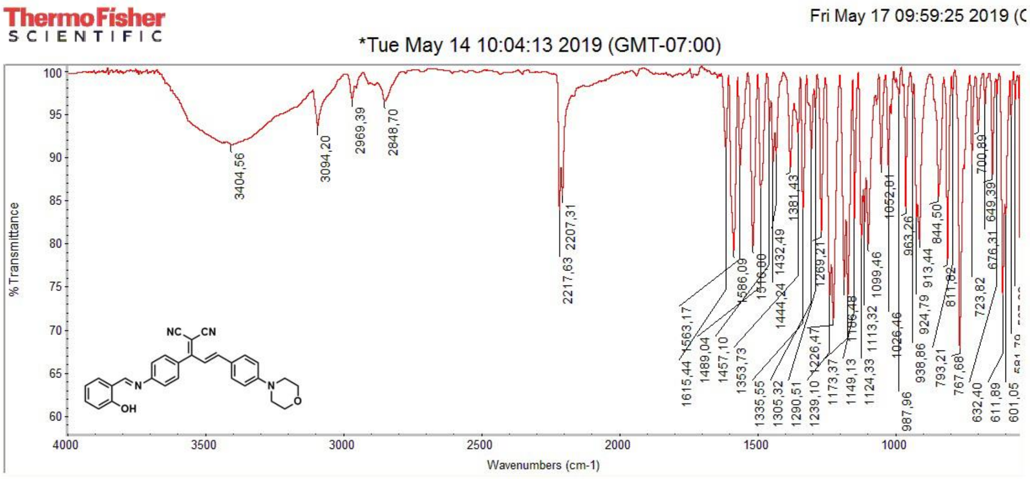

Figure S33: FTIR spectrum of dye **11**.

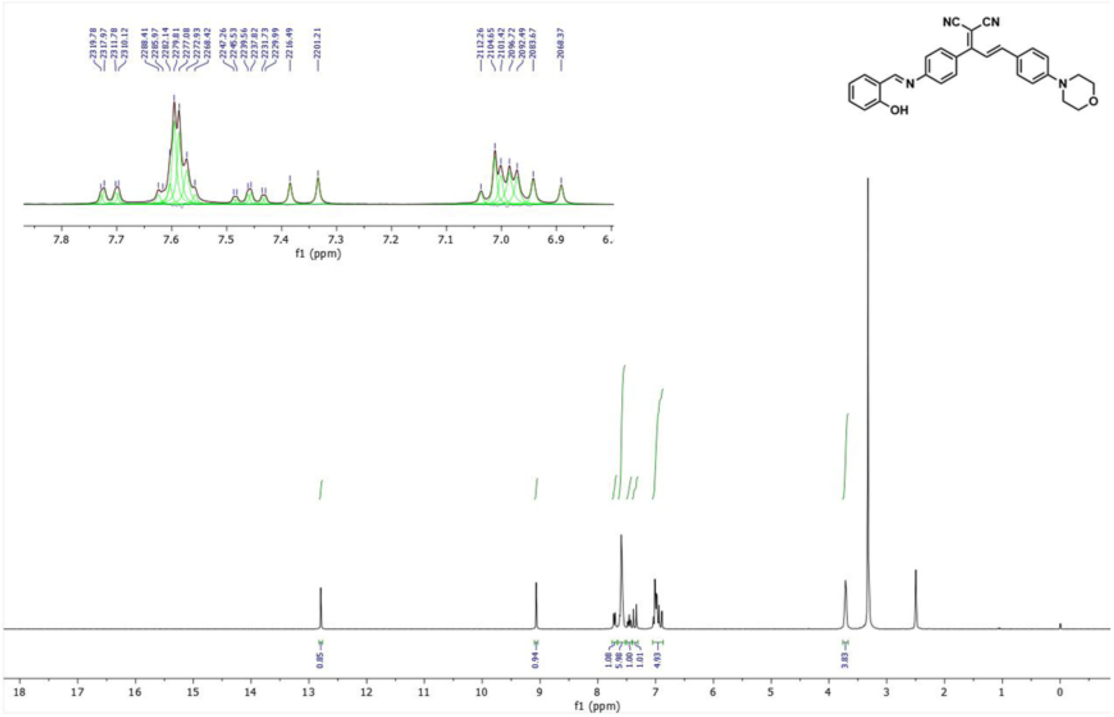

Figure S34: ^{1}H NMR spectrum of dye **11** in DMSO-d_6.

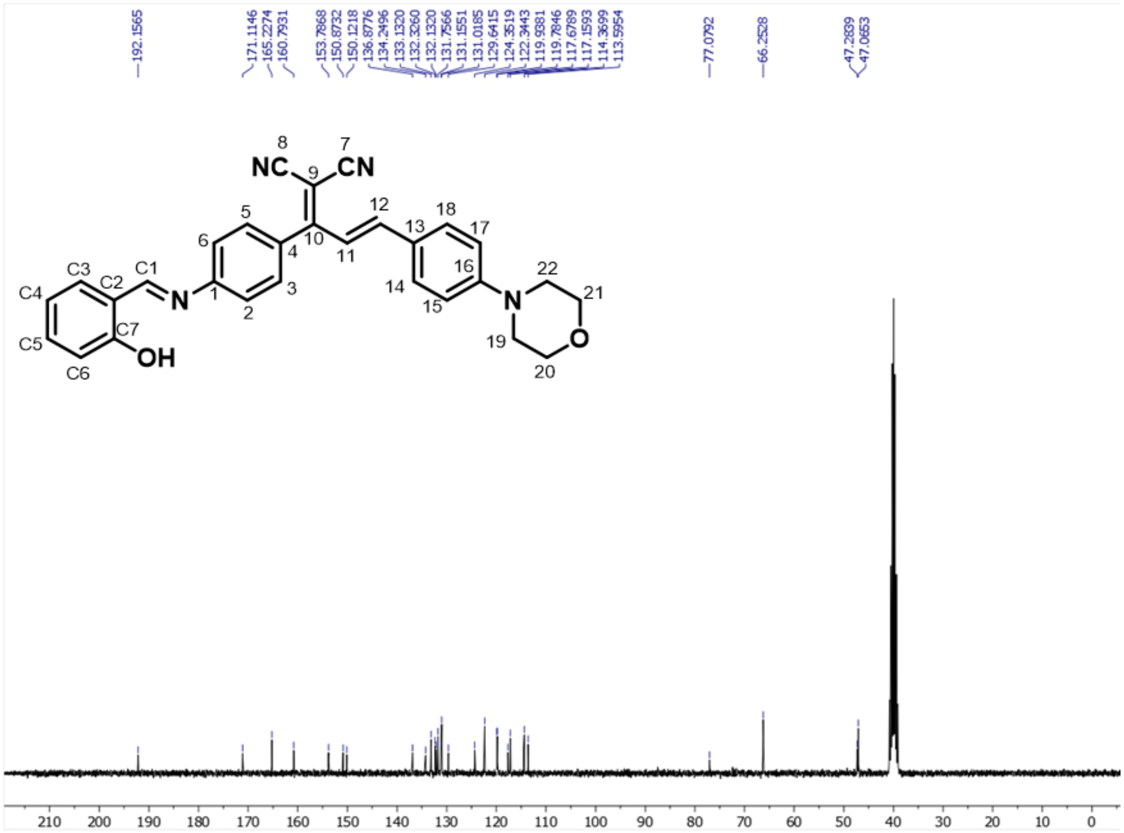

Figure S35: ^{13}C NMR spectrum of dye **11** in DMSO-d_6.

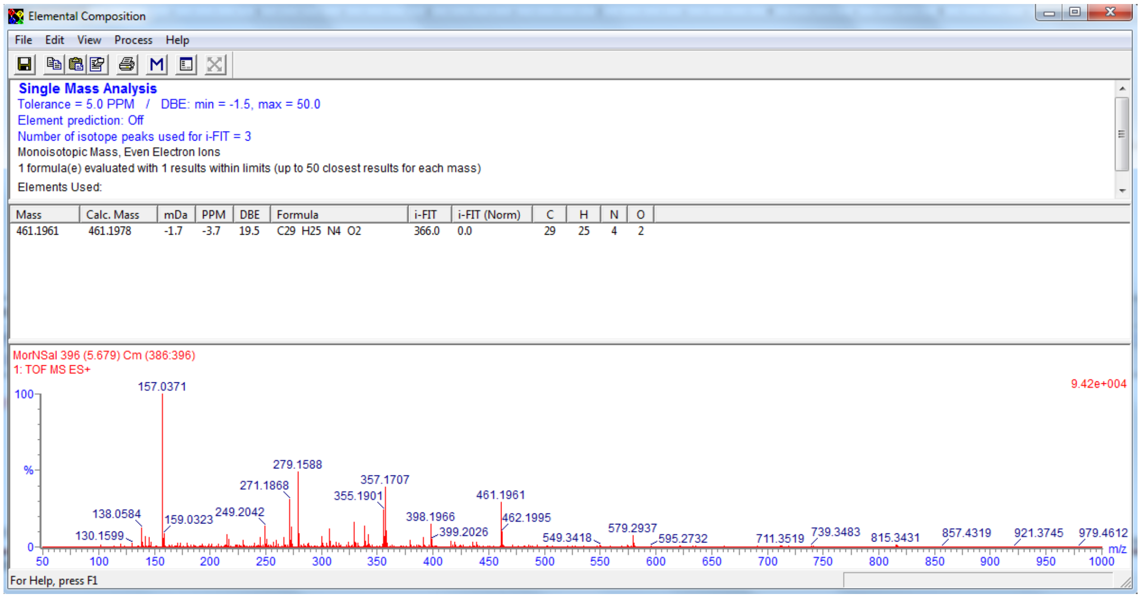

Figure S36: HRMS spectrum of dye **11**.

2.10. FTIR, ^{1}H NMR, ^{13}C NMR and HRMS spectra of dye 12

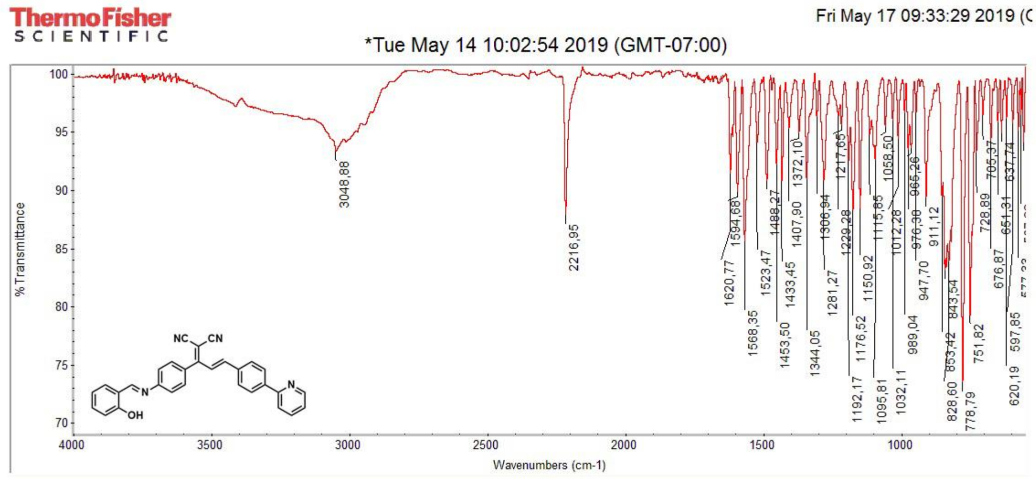

Figure S37: FTIR spectrum of dye **12**.

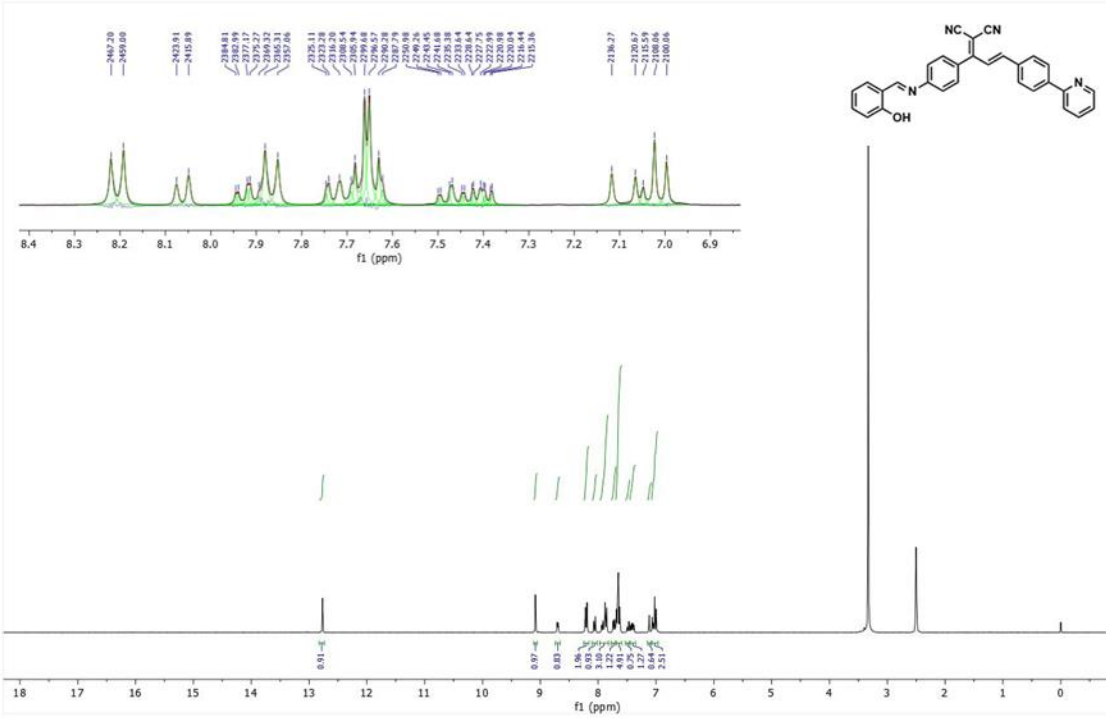

Figure S38: ^{1}H NMR spectrum of dye **12** in DMSO-d_6.

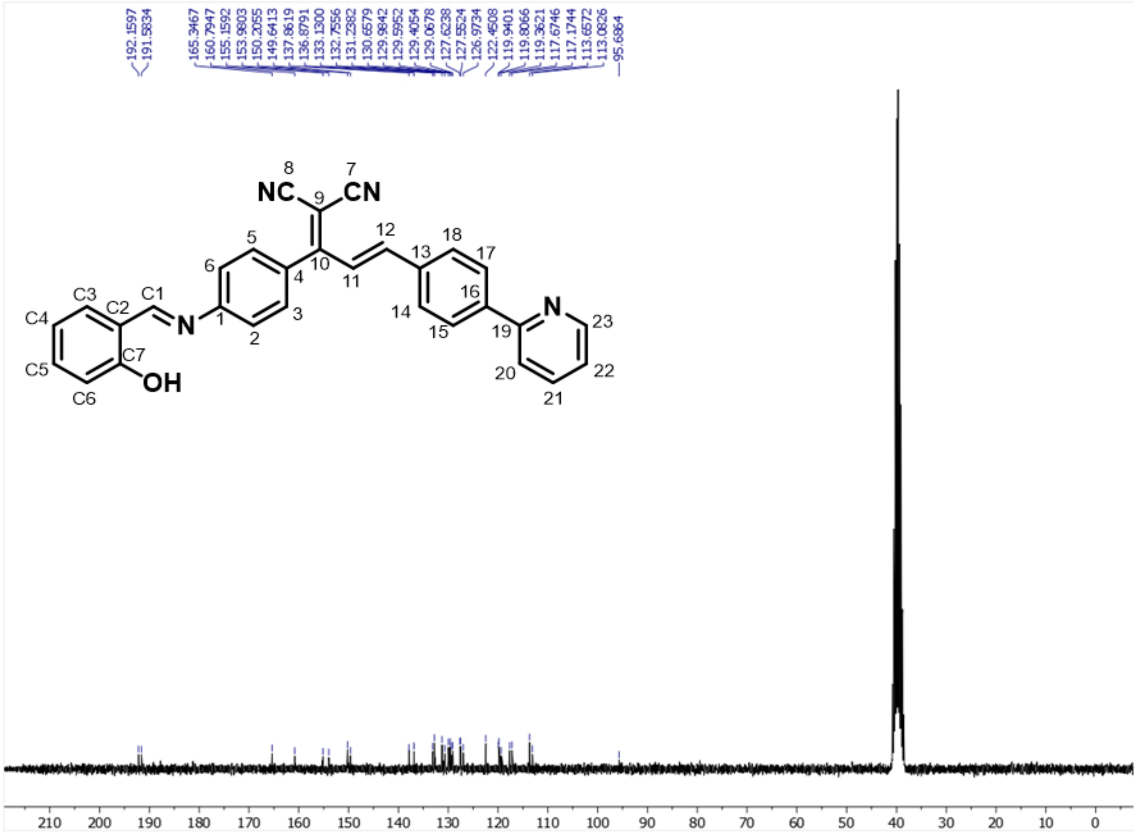

Figure S39: ^{13}C NMR spectrum of dye **12** in DMSO-d_6.

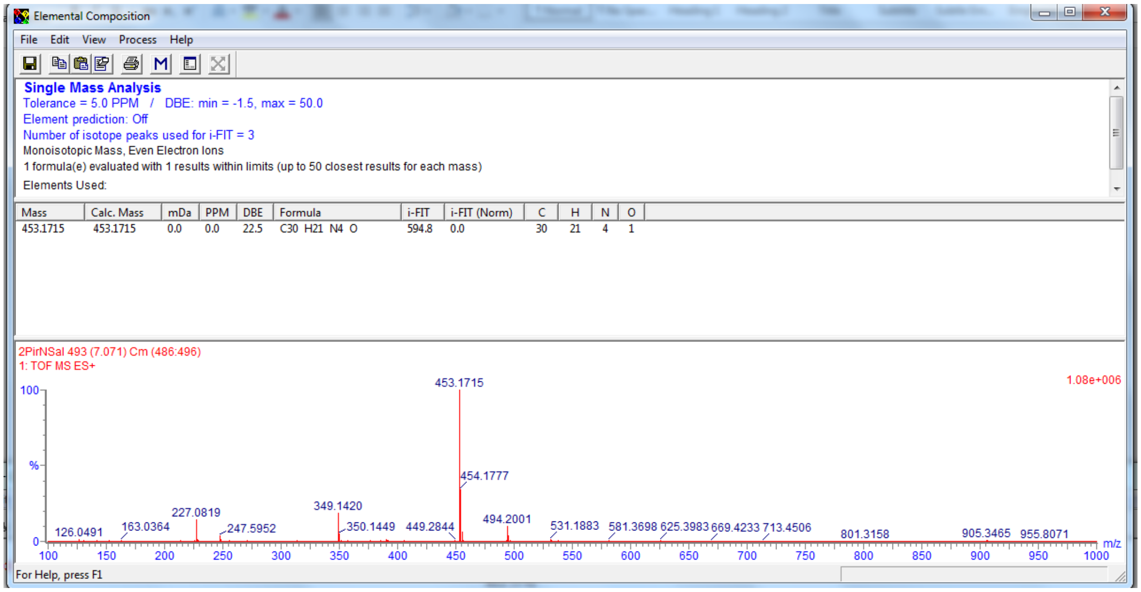

Figure S40: HRMS spectrum of dye **12**.

3. Calculated data

3.1. Optimized geometries of dyes **4–7** and **9–12**

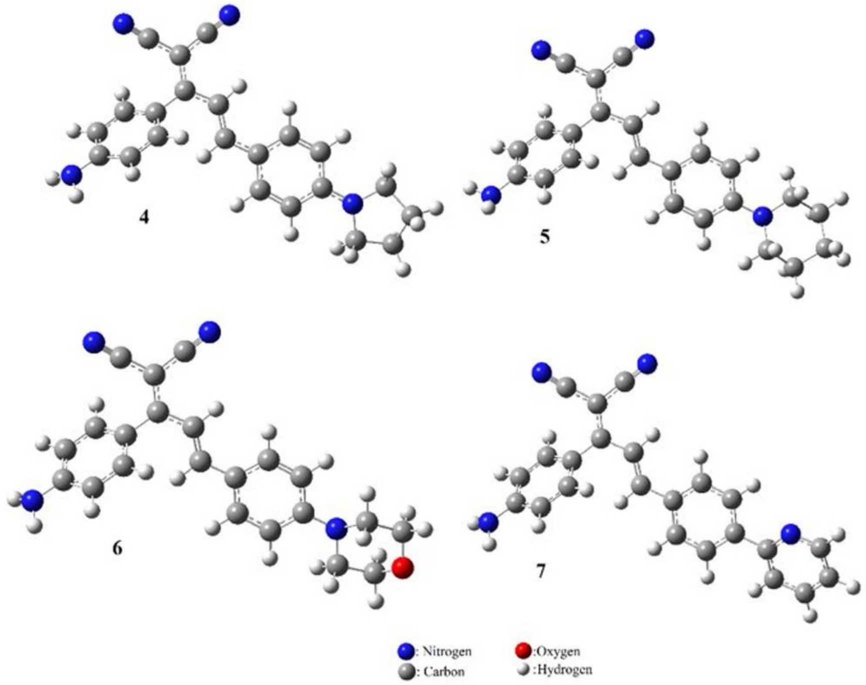

Figure S41: The optimized geometries of **4–7**.

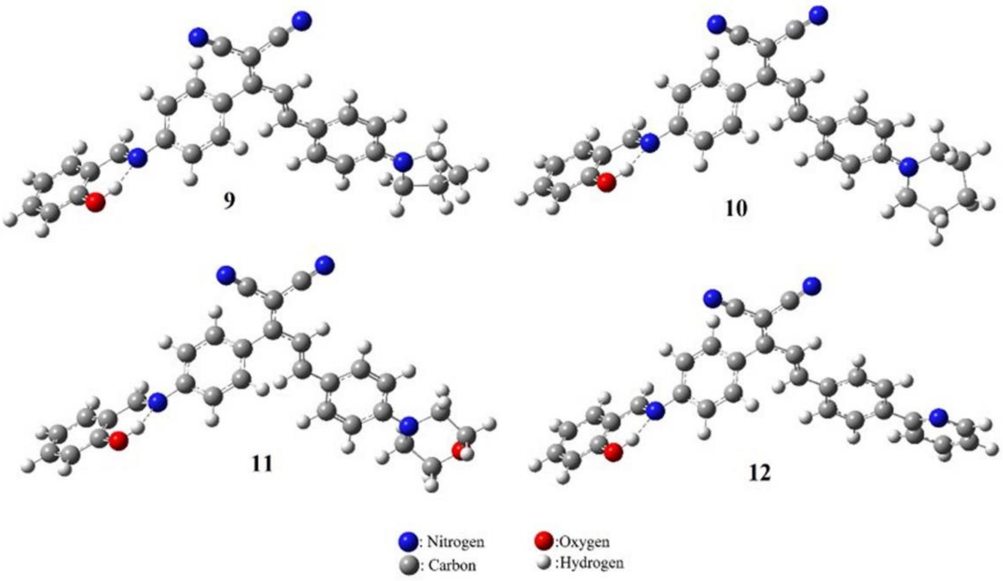

Figure S42: The optimized geometries of **9–12**.

3.2. The experimental and calculated photophysical properties of dyes 8-12

Table S1. Photophysical properties of dyes **8-12** in various solvents with different polarity and calculated absorption spectra data.

		Experimental						***Calculated***		
	Solvents	λ^{abs}_{max} (nm)	λ^{em}_{max} (nm)	*Stokes shift* (nm)	*Stokes shift* (cm^{-1})	$\Phi_F{}^a$	ε ($mM^{-1}cm^{-1}$)	λ^{abs}_{max} (nm)	*f*	Transitions, w(%)
**8**	ACN (45.6[c])	543	644	101	2893	<0.01	51.4	521	1.0973	HOMO→LUMO,98.1
	DMSO (45.1[c])	562	650	88	2434	<0.01	53.0	524	1.1264	HOMO→LUMO,98.4
	DCM (40.7[c])	546	636	90	2609	<0.01	60.2	520	1.1172	HOMO→LUMO,98.4
	THF (37.4[c])	534	630	96	2871	<0.01	58.5	518	1.1075	HOMO→LUMO,98.3
	PhMe (33.9[c])	529	610	81	2517	0.11	58.6	509	1.1082	HOMO→LUMO,98.4
**9**	ACN	510	623	113	3565	<0.01	58.3	501	1.1728	HOMO→LUMO,98.1
	DMSO	518	630	113	3461	<0.01	54.3	505	1.1971	HOMO→LUMO,98.3
	DCM	515	612	97	3086	0.01	40.5	501	1.1906	HOMO→LUMO,98.2
	THF	506	610	104	3389	<0.01	54.2	499	1.1827	HOMO→LUMO,98.1
	PhMe	504	581	77	2624	<0.01	55.8	491	1.1862	HOMO→LUMO,98.1
**10**	ACN	495	622	127	4156	<0.01	45.7	504	1.1696	HOMO→LUMO,98.2
	DMSO	491	636	145	4643	<0.01	46.9	508	1.1929	HOMO→LUMO,98.4
	DCM	500	614	114	3724	0.01	33.6	503	1.1865	HOMO→LUMO,98.3
	THF	491	612	121	4037	<0.01	41.6	501	1.1787	HOMO→LUMO,98.2
	PhMe	487	581	94	3331	<0.01	43.8	493	1.1808	HOMO→LUMO,98.2
**11**	ACN	466	618	152	5296	<0.01	42.0	486	1.1419	HOMO→LUMO,96.8
	DMSO	467	625	158	5446	<0.01	36.5	489	1.1618	HOMO→LUMO,97.2
	DCM	470	598	128	4565	0.01	28.4	485	1.1593	HOMO→LUMO,96.8
	THF	467	602	135	4802	0.01	39.5	483	1.1535	HOMO→LUMO,96.8
	PhMe	464	563	99	3783	<0.01	41.6	476	1.1661	HOMO→LUMO,96.4
**12**	ACN	377	459	82	4783	n.d	52.7	428	1.1826	HOMO-1→LUMO,52.4<br>HOMO→LUMO,46.0
	DMSO	385	496	111	5796	n.d	50.5	430	1.1910	HOMO-1→LUMO,51.1<br>HOMO→LUMO,47.6
	DCM	381	473	92	5114	n.d	30.4	430	1.2153	HOMO-1→LUMO,55.8<br>HOMO→LUMO,42.7
	THF	380	458	78	4501	n.d	56.9	429	1.2175	HOMO-1→LUMO,56.7<br>HOMO→LUMO,41.7
	PhMe	384	578	194	8729	n.d	53.1	428	1.2858	HOMO-1→LUMO,58.3<br>HOMO→LUMO,39.8

[a]Solvents arranged in order of decreasing $E_T{}^{30}$ values. [b]Fluorescence quantum yield (±10%) determined relative to fluorescein in pH=9 solution (Φ_F= 0.95) as standard. [c]The values for relative polarity are taken from [1]. n.d, it couldn't be determined.

4. Photophysical properties of dyes 3–12

4.1. Calculation formula for relative quantum yield

Relative Quantum Yields of all synthesized compound are measured using equation below by taking Fluorescein (Φ_F = 0.95 in pH 9) as a refence.

$$\Phi_s = \Phi_r \times \frac{A_r}{A_s} \times \frac{I_s}{I_r} \times \frac{n_s^2}{n_r^2}$$

Φ	*: Quantum Yield*
A	*: Maximum absorbance value (0.055 < A < 0.095)*
I	*: Maximum Fluorescence value*
n	*: Solvent's refractive index*
Subscript "s"	*: Sample*
Subscript "r"	*: Reference*

4.2. Photoluminescence properties of dye 3

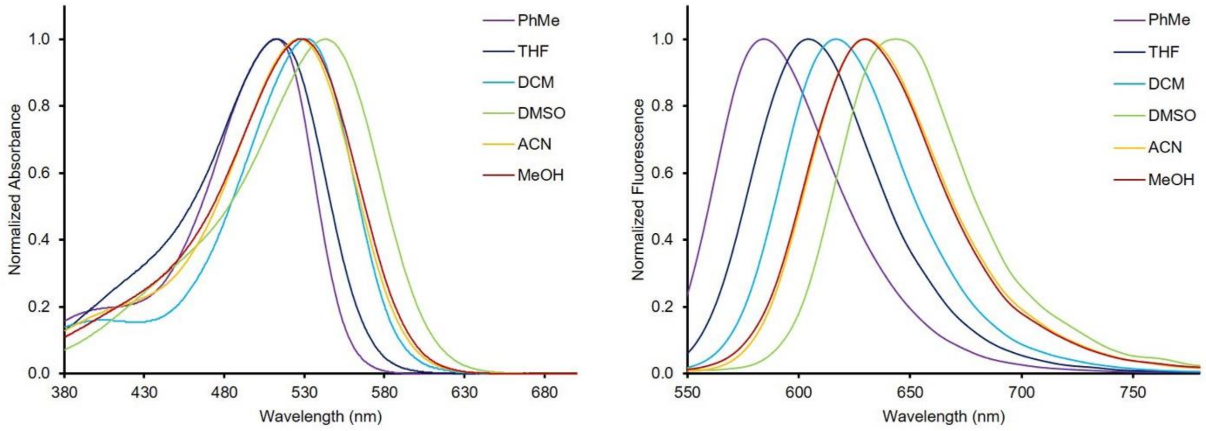

Figure S43: Normalized absorption (left) and fluorescence (right) spectra of dye **3** in various solvents with different polarity.

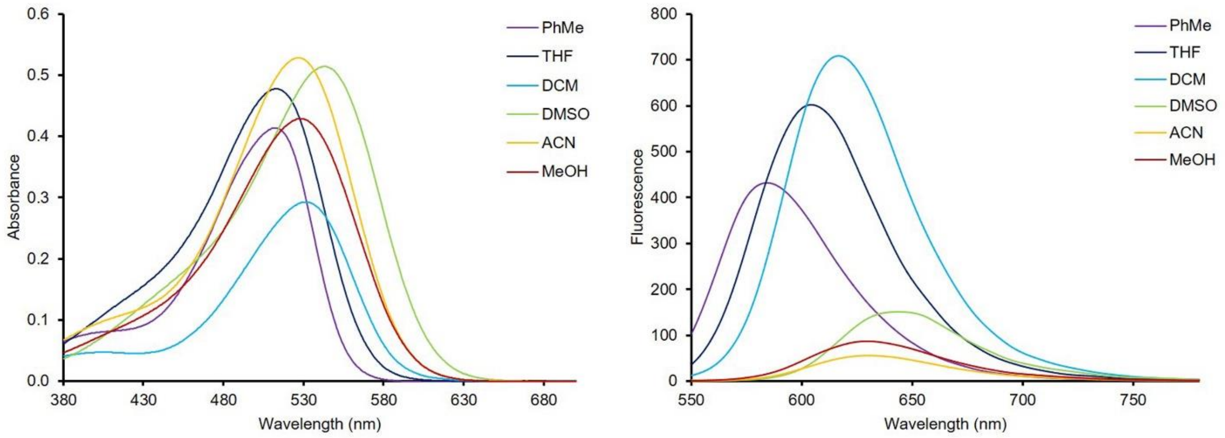

Figure S44: Absorption (left) and fluorescence (right) spectra of dye **3** in various solvents with different polarity.

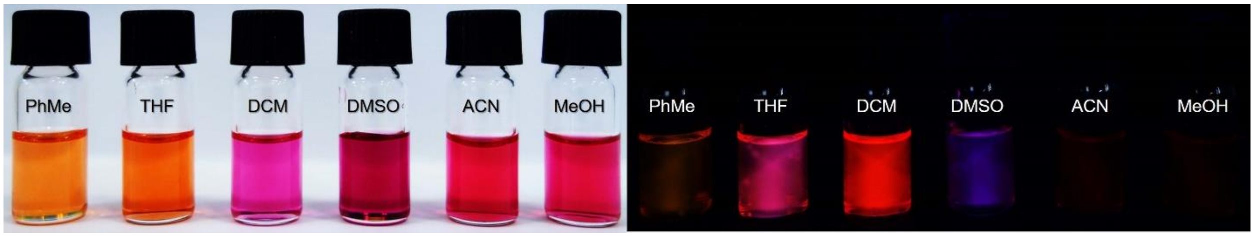

Figure S45: Photographs of dye **3** under ambient light (left) and 365 nm UV lamp (right) in various solvents with different polarity.

Photophysical properties of dye **3** in various solvents with different polarity

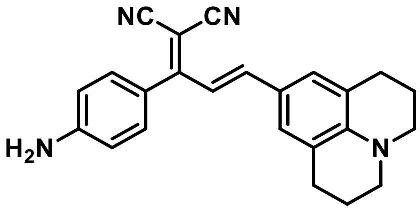

Dye **3**

Solvent	λ^{abs}_{max} (nm)	λ^{em}_{max} (nm)	*Stokes shift* (nm)	*Stokes shift* (cm^{-1})	Φ_F	ε ($mM^{-1}cm^{-1}$)
MeOH	528	630	102	3074	<0.01	42.6
ACN	527	630	103	3125	<0.01	51.7
DMSO	543	644	101	2879	<0.01	49.8
DCM	531	617	86	2632	0.01	36.8
THF	513	604	91	2961	0.01	45.9
PhMe	512	584	72	2414	<0.01	41.4

4.3. Photoluminescence properties of dye 4

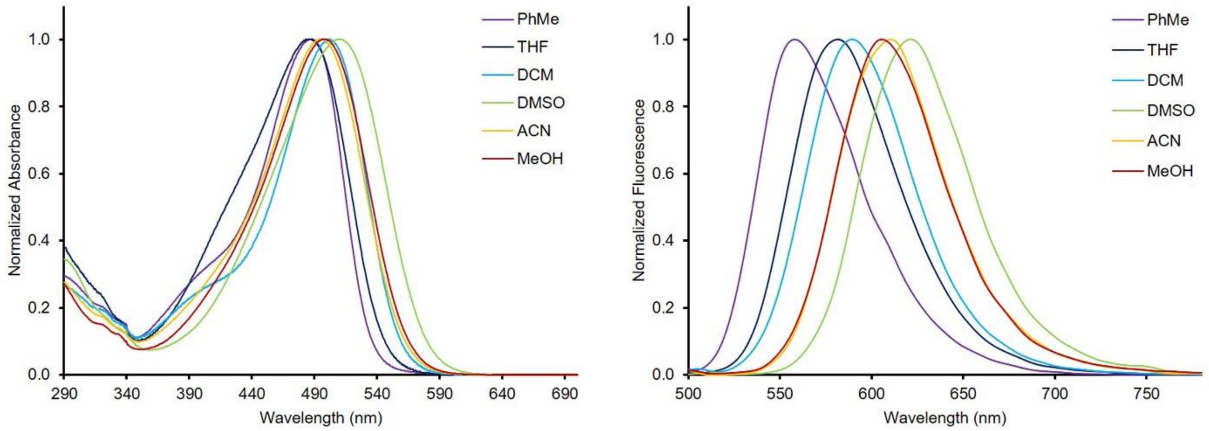

Figure S46: Normalized absorption (left) and fluorescence (right) spectra of dye **4** in various solvents with different polarity.

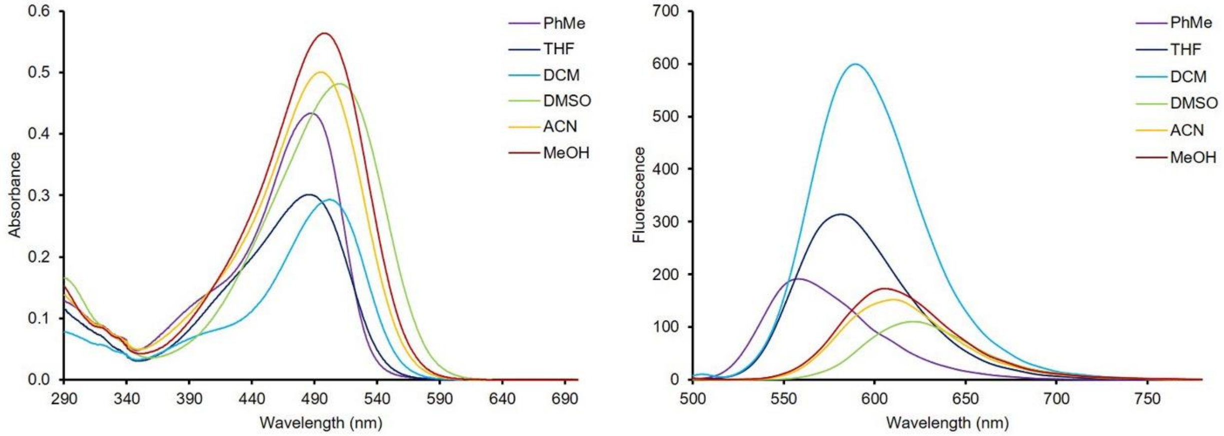

Figure S47: Absorption (left) and fluorescence (right) spectra of dye **4** in various solvents with different polarity.

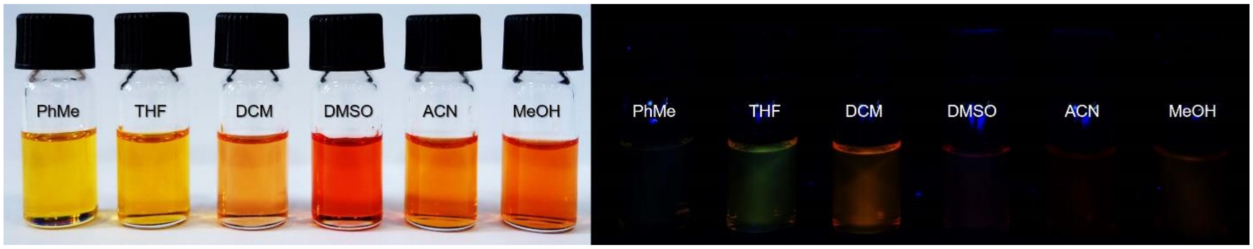

Figure S48: Photographs of dye **4** under ambient light (left) and 365 nm UV lamp (right) in various solvents with different polarity.

Photophysical properties of dye **4** in various solvents with different polarity

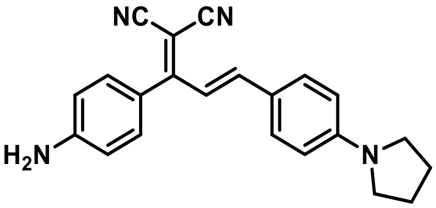

Dye **4**

Solvent	λ^{abs}_{max} (nm)	λ^{em}_{max} (nm)	*Stokes shift* (nm)	*Stokes shift* (cm^{-1})	Φ_F	ε ($mM^{-1}cm^{-1}$)
MeOH	498	606	108	3568	<0.01	55.4
ACN	495	611	116	3819	<0.01	48.6
DMSO	510	622	112	3515	<0.01	49.6
DCM	502	589	87	2954	0.01	30.1
THF	486	582	96	3403	<0.01	33.7
PhMe	488	558	70	2592	<0.01	41.9

4.4. Photoluminescence properties of dye 5

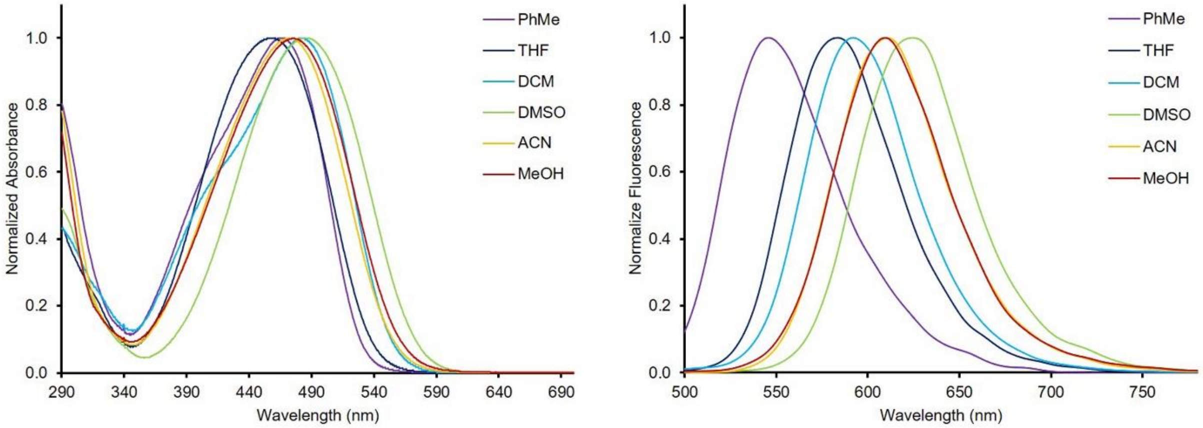

Figure S49: Normalized absorption (left) and fluorescence (right) spectra of dye **5** in various solvents with different polarity.

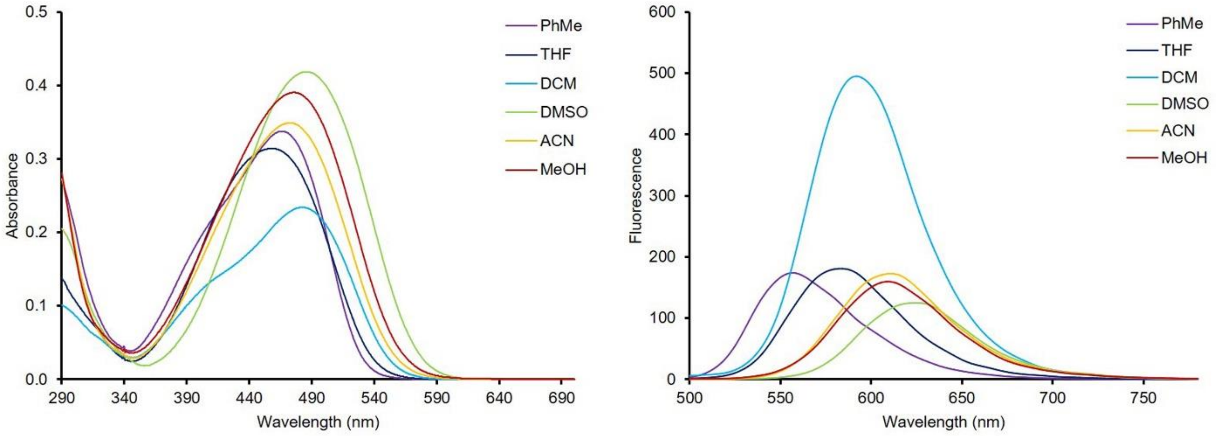

Figure S50: Absorption (left) and fluorescence (right) spectra of dye **5** in various solvents with different polarity.

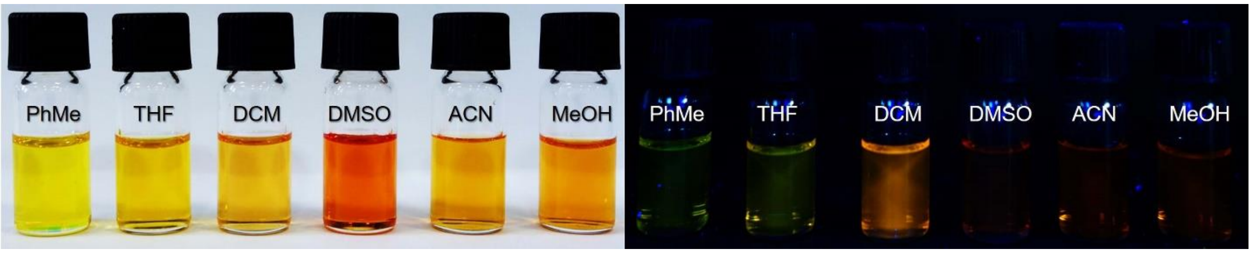

Figure S51: Photographs of dye **5** under ambient light (left) and 365 nm UV lamp (right) in various solvents with different polarity.

Photophysical properties of dye **5** in various solvents with different polarity

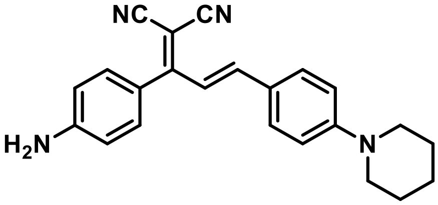

Dye **5**

Solvent	λ^{abs}_{max} (nm)	λ^{em}_{max} (nm)	*Stokes shift* (nm)	*Stokes shift* (cm^{-1})	Φ_F	ε ($mM^{-1}cm^{-1}$)
MeOH	476	609	133	4599	<0.01	39.9
ACN	473	611	134	4770	<0.01	35.9
DMSO	486	624	138	4561	<0.01	42.1
DCM	483	592	109	3828	0.01	25.3
THF	457	583	126	4741	<0.01	31.4
PhMe	467	557	90	3476	<0.01	33.9

4.5. Photoluminescence properties of dye 6

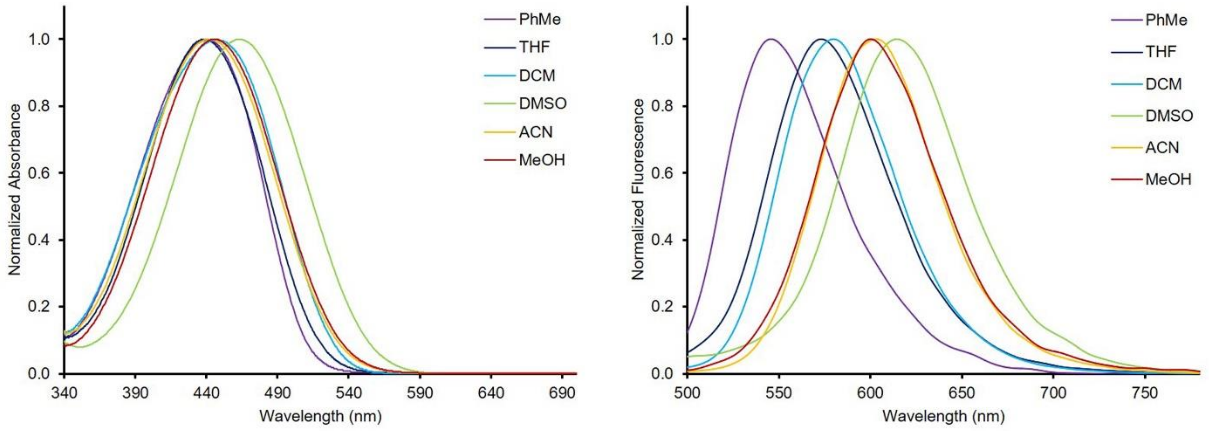

Figure S52: Normalized absorption (left) and fluorescence (right) spectra of dye **6** in various solvents with different polarity.

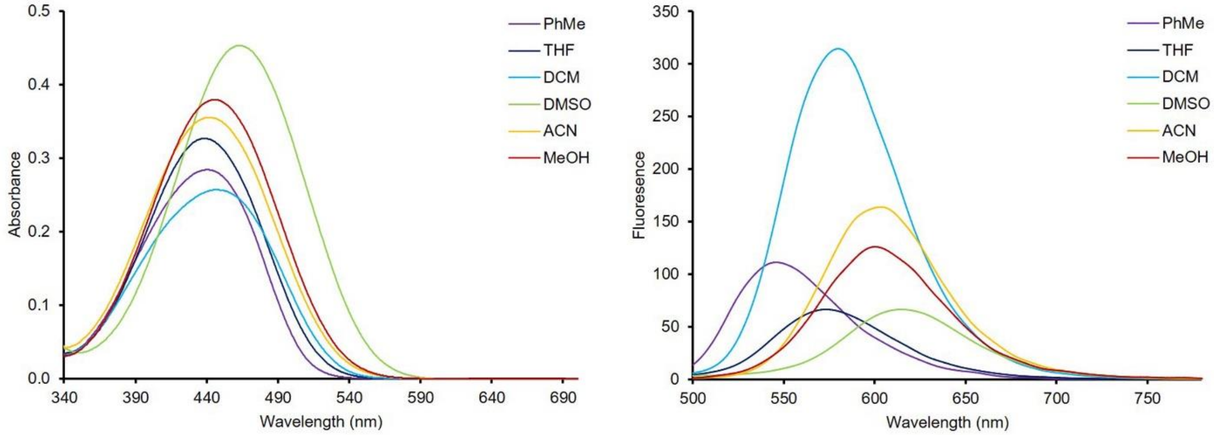

Figure S53: Absorption (left) and fluorescence (right) spectra of dye **6** in various solvents with different polarity.

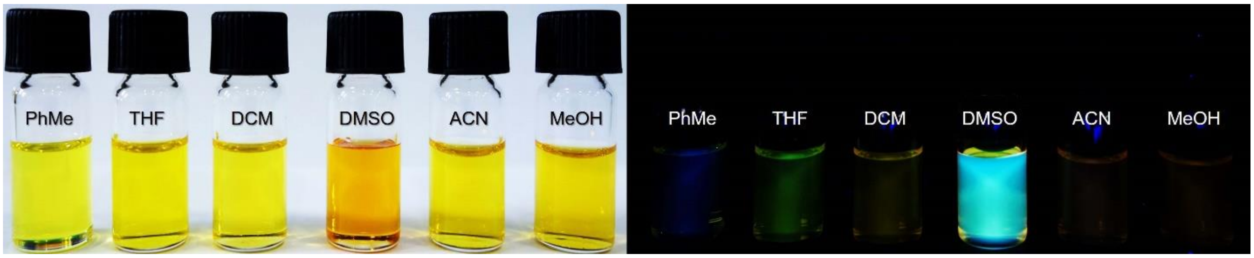

Figure S54: Photographs of dye **6** under ambient light (left) and 365 nm UV lamp (right) in various solvents with different polarity.

Photophysical properties of dye **6** in various solvents with different polarity

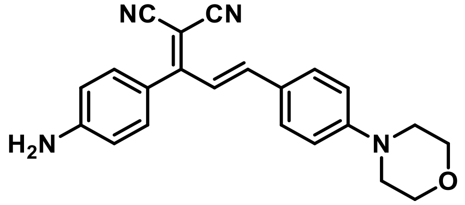

Dye **6**

Solvent	λ^{abs}_{max} (nm)	λ^{em}_{max} (nm)	*Stokes shift* (nm)	*Stokes shift* (cm^{-1})	Φ_F	ε ($mM^{-1}cm^{-1}$)
MeOH	446	600	154	5786	<0.01	40.8
ACN	442	603	161	6052	<0.01	34.6
DMSO	463	614	151	5346	<0.01	42.7
DCM	448	580	132	5099	<0.01	27.8
THF	439	573	134	5359	<0.01	34.7
PhMe	440	546	106	4399	<0.01	30.6

4.6. Photoluminescence properties of dye 7

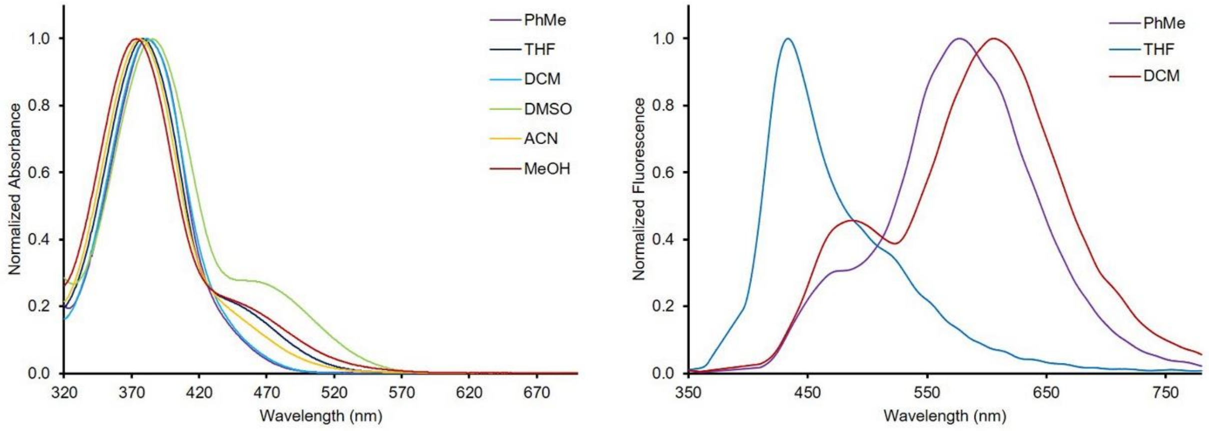

Figure S55: Normalized absorption (left) and fluorescence (right) spectra of dye **7** in various solvents with different polarity.

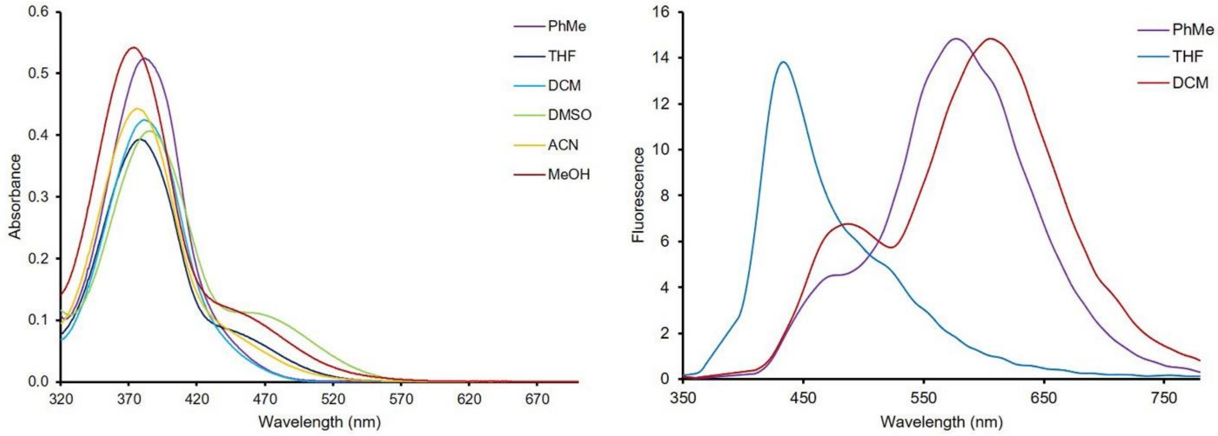

Figure S56: Absorption (left) and fluorescence (right) spectra of dye **7** in various solvents with different polarity.

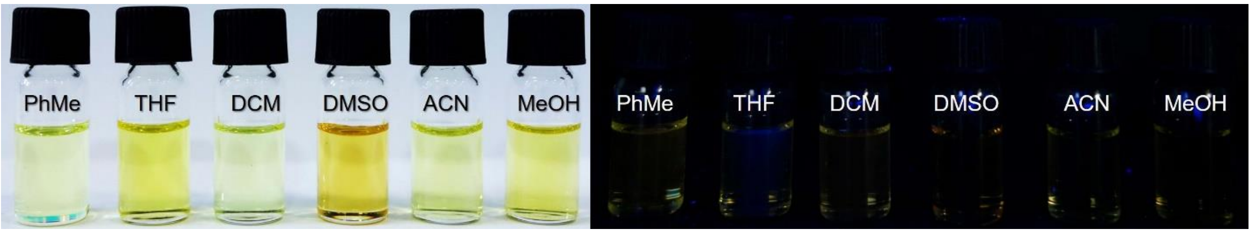

Figure S57: Photographs of dye **7** under ambient light (left) and 365 nm UV lamp (right) in various solvents with different polarity.

Photophysical properties of dye **7** in various solvents with different polarity

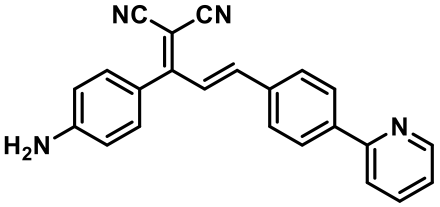

Dye **7**

Solvent	λ^{abs}_{max} (nm)	λ^{em}_{max} (nm)	*Stokes shift* (nm)	*Stokes shift* (cm^{-1})	Φ_F	ε (mM^{-1}cm^{-1})
MeOH	374	-	-	-	-	51.4
ACN	376	-	-	-	-	43.4
DMSO	386	-	-	-	-	40.4
DCM	382	606	224	9705	n.d	42.1
THF	379	434	55	3357	n.d	40.4
PhMe	382	577	195	8853	n.d	53.1

n.d, it couldn't be determined.

4.7. Photoluminescence properties of dye 8

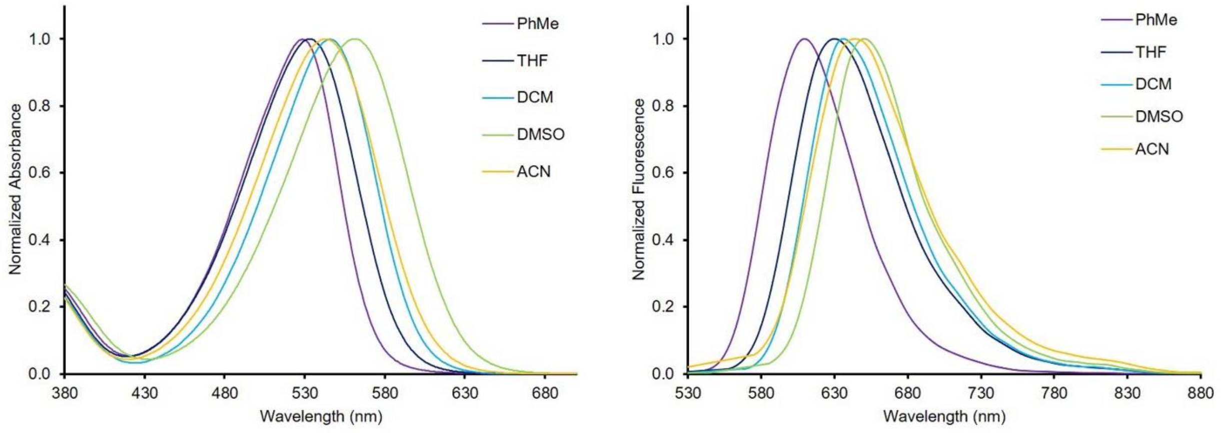

Figure S58: Normalized absorption (left) and fluorescence (right) spectra of dye **8** in various solvents with different polarity.

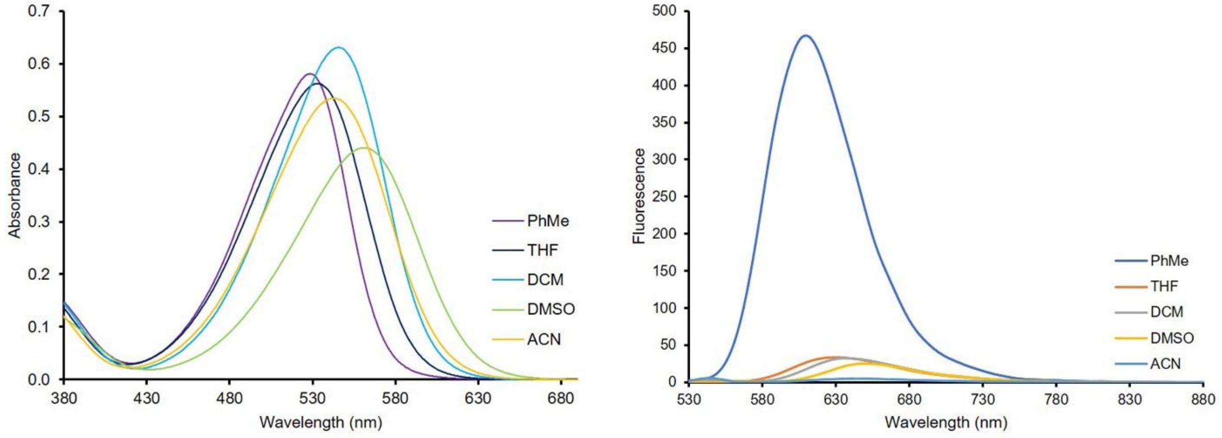

Figure S59: Absorption (left) and fluorescence (right) spectra of dye **8** in various solvents with different polarity.

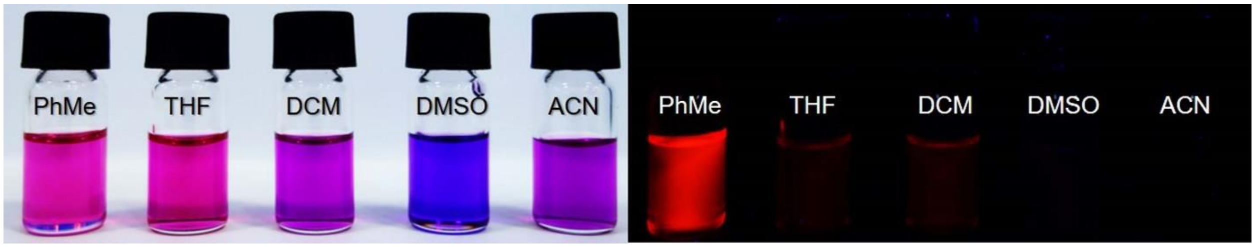

Figure S60: Photographs of dye **8** under ambient light (left) and 365 nm UV lamp (right) in various solvents with different polarity.

Photophysical properties of dye **8** in various solvents with different polarity

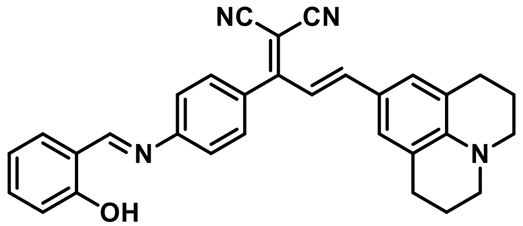

Dye **8**

Solvent	λ^{abs}_{max} (nm)	λ^{em}_{max} (nm)	*Stokes shift* (nm)	*Stokes shift* (cm^{-1})	Φ_F	ε ($mM^{-1}cm^{-1}$)
ACN	543	644	101	2893	<0.01	51.4
DMSO	562	650	88	2434	<0.01	53.0
DCM	546	636	90	2609	<0.01	60.2
THF	534	630	96	2871	<0.01	58.5
PhMe	529	610	81	2517	0.01	58.6

4.8. Photoluminescence properties of dye 9

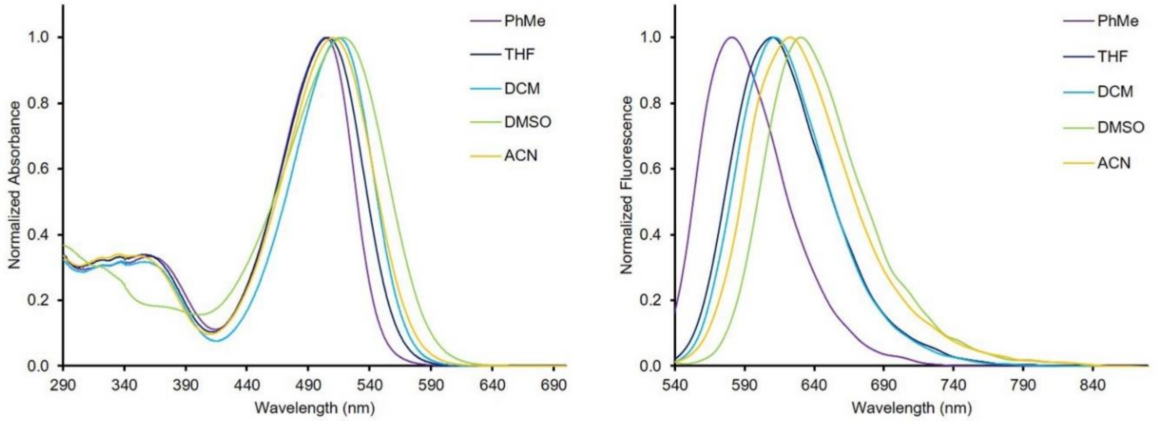

Figure S61: Normalized absorption (left) and fluorescence (right) spectra of dye **9** in various solvents with different polarity.

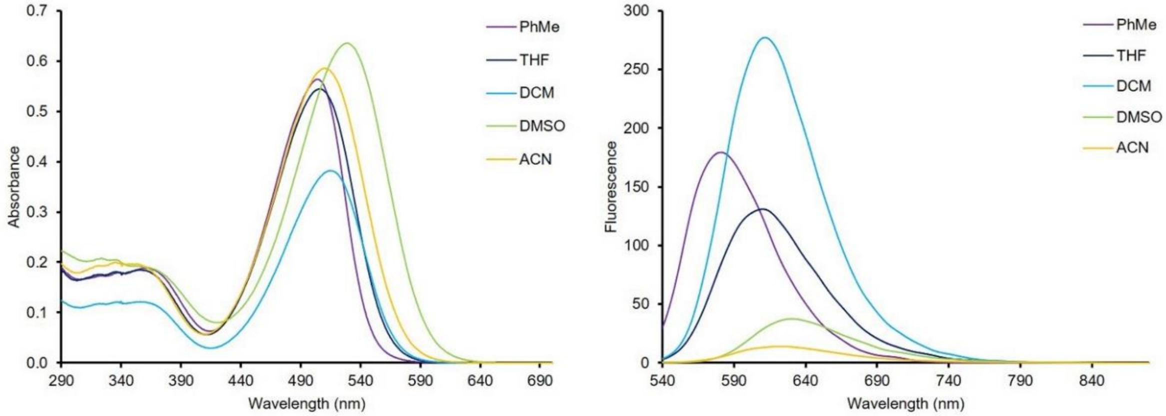

Figure S62: Absorption (left) and fluorescence (right) spectra of dye **9** in various solvents with different polarity.

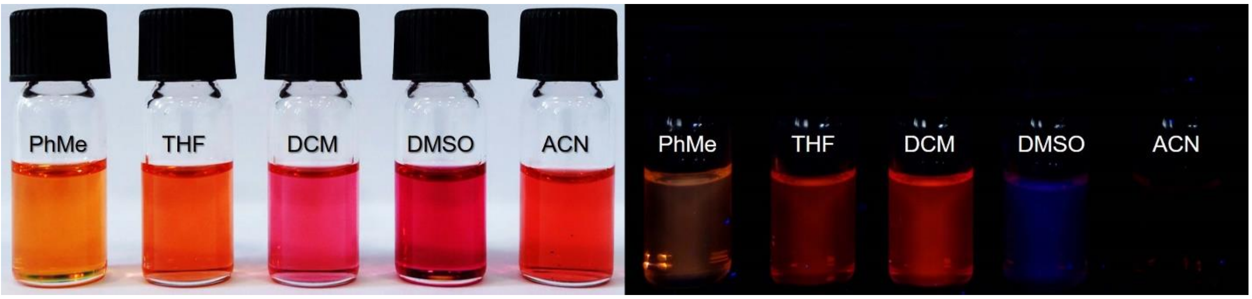

Figure S63: Photographs of dye **9** under ambient light (left) and 365 nm UV lamp (right) in various solvents with different polarity.

Photophysical properties of dye **9** in various solvents with different polarity

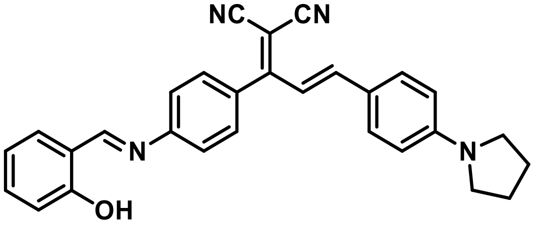

Dye **9**

Solvent	λ^{abs}_{max} (nm)	λ^{em}_{max} (nm)	*Stokes shift* (nm)	*Stokes shift* (cm^{-1})	Φ_F	ε ($mM^{-1}cm^{-1}$)
ACN	510	623	113	3565	<0.01	58.2
DMSO	518	630	113	3461	<0.01	54.3
DCM	515	612	97	3086	0.01	40.5
THF	506	610	104	3389	<0.01	54.2
PhMe	504	581	77	2624	<0.01	55.8

4.9. Photoluminescence properties of dye 10

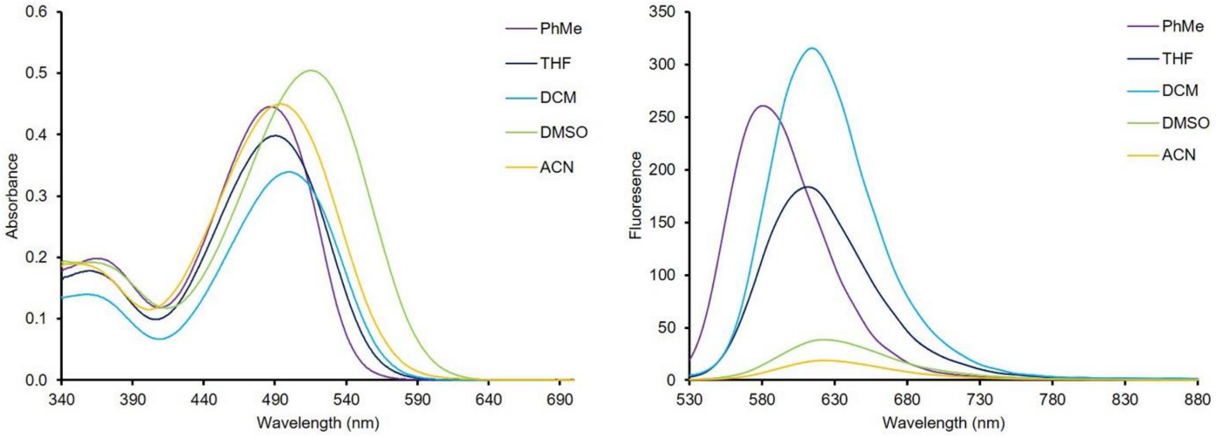

Figure S64: Normalized absorption (left) and fluorescence (right) spectra of dye **10** in various solvents with different polarity.

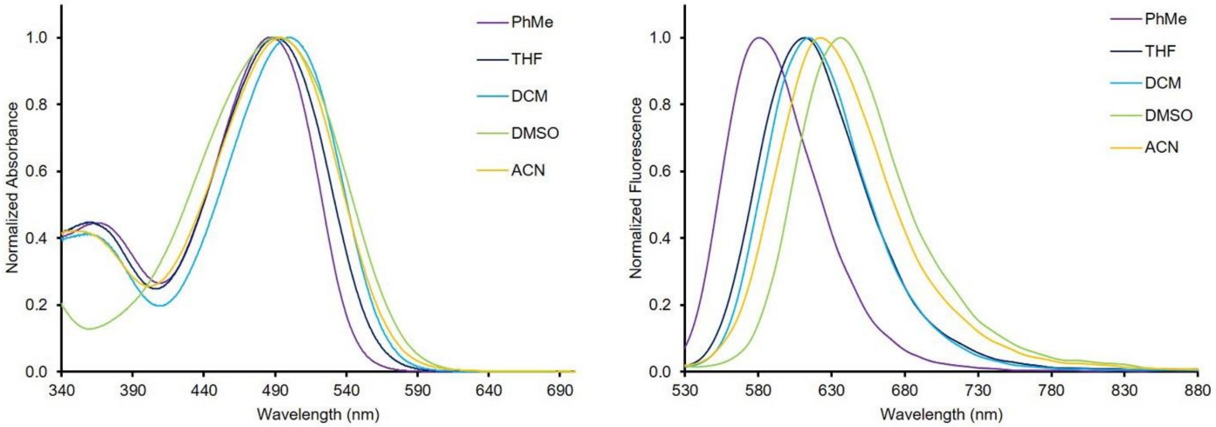

Figure S65: Absorption (left) and fluorescence (right) spectra of dye **10** in various solvents with different polarity.

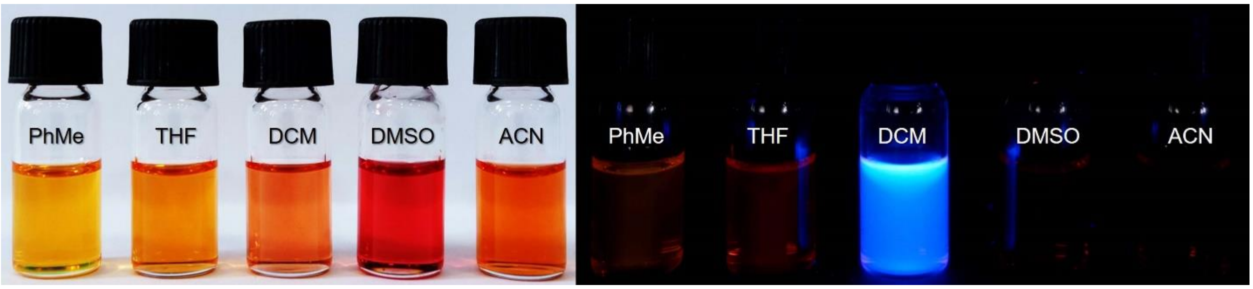

Figure S66: Photographs of dye **10** under ambient light (left) and 365 nm UV lamp (right) in various solvents with different polarity.

Photophysical properties of dye **10** in various solvents with different polarity

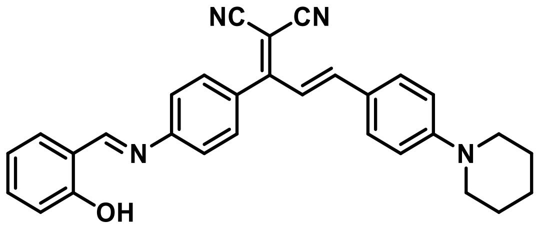

Dye **10**

Solvent	λ^{abs}_{max} (nm)	λ^{em}_{max} (nm)	*Stokes shift* (nm)	*Stokes shift* (cm^{-1})	Φ_F	ε ($mM^{-1}cm^{-1}$)
ACN	495	622	127	4156	<0.01	45.7
DMSO	491	636	145	4643	<0.01	46.9
DCM	500	614	114	3724	<0.01	33.6
THF	491	612	121	4037	<0.01	41.6
PhMe	487	581	94	3331	<0.01	43.8

4.10. Photoluminescence properties of dye 11

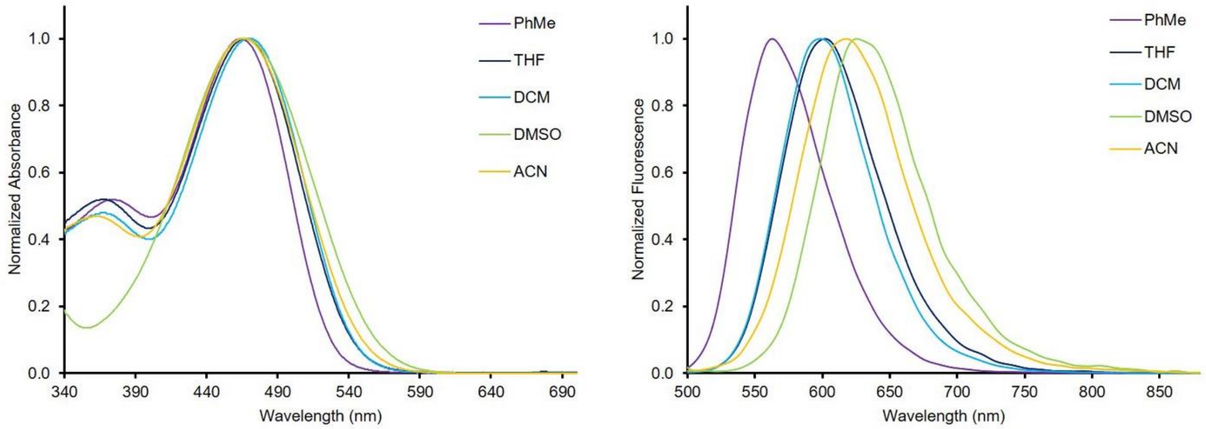

Figure S67: Normalized absorption (left) and fluorescence (right) spectra of dye **11** in various solvents with different polarity.

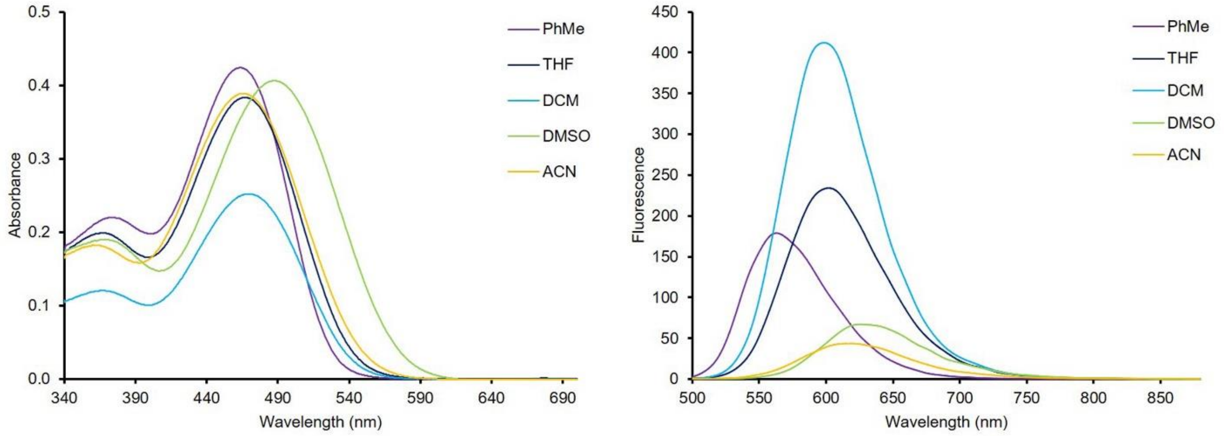

Figure S68: Absorption (left) and fluorescence (right) spectra of dye **11** in various solvents with different polarity.

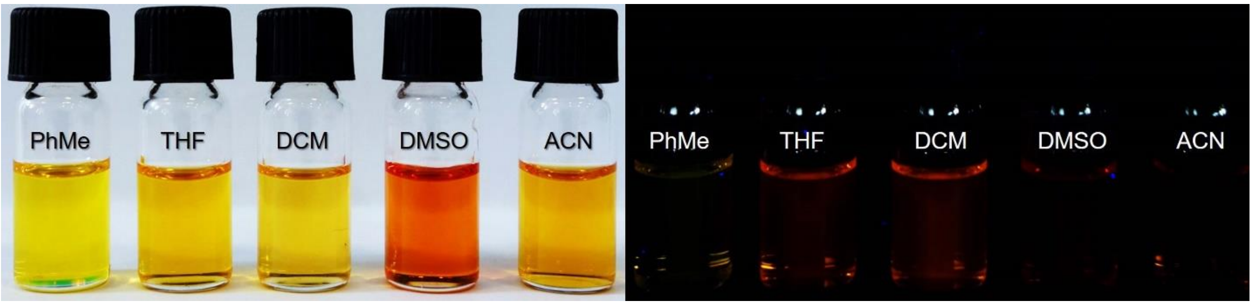

Figure S69: Photographs of dye **11** under ambient light (left) and 365 nm UV lamp (right) in various solvents with different polarity

Photophysical properties of dye **11** in various solvents with different polarity

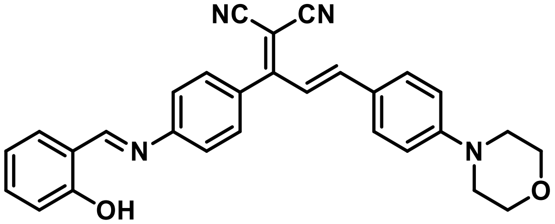

Dye **11**

Solvent	λ^{abs}_{max} (nm)	λ^{em}_{max} (nm)	*Stokes shift* (nm)	*Stokes shift* (cm^{-1})	Φ_F	ε ($mM^{-1}cm^{-1}$)
ACN	466	618	152	5296	0.01<	42.0
DMSO	467	625	158	5446	0.01<	36.5
DCM	470	598	128	4565	0.01<	28.4
THF	467	602	135	4802	0.01	39.5
PhMe	464	563	99	3783	0.01<	41.6

4.11. Photoluminescence properties of dye 12

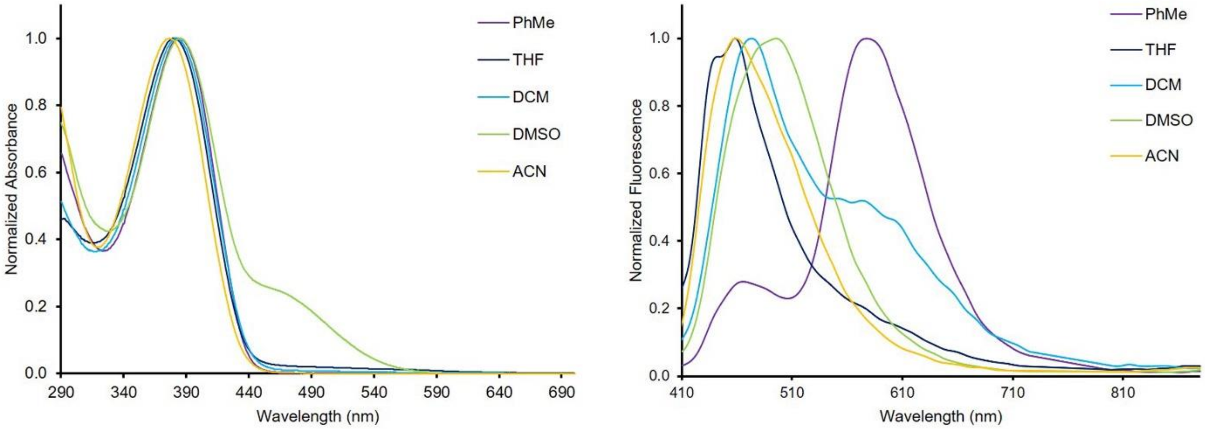

Figure S70: Normalized absorption (left) and fluorescence (right) spectra of dye **12** in various solvents with different polarity.

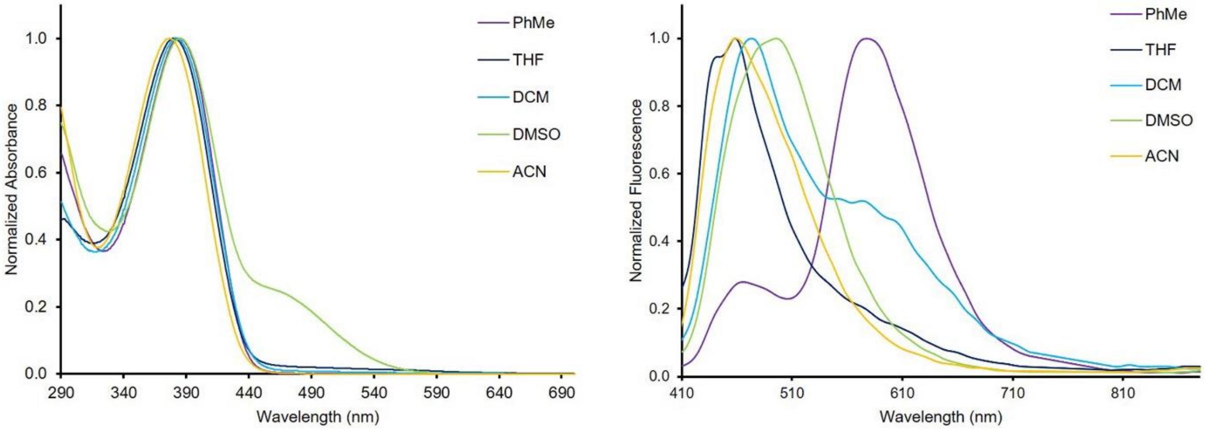

Figure S71: Absorption (left) and fluorescence (right) spectra of dye **12** in various solvents with different polarity.

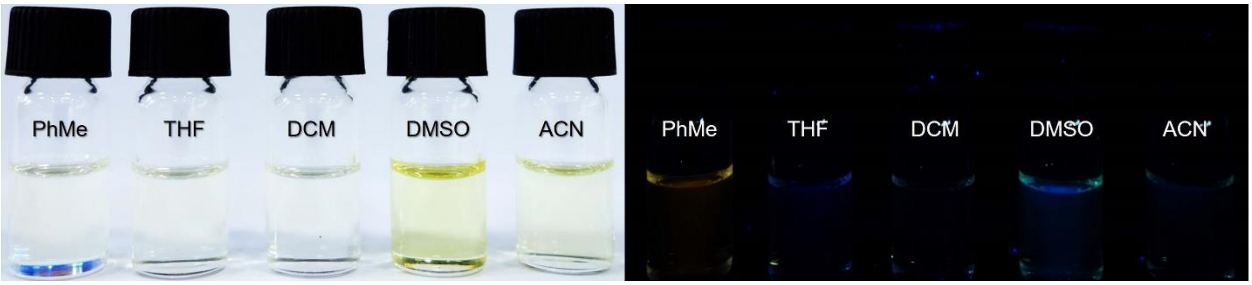

Figure S72: Photographs of dye **12** under ambient light (left) and 365 nm UV lamp (right) in various solvents with different polarity.

Photophysical properties of dye **12** in various solvents with different polarity

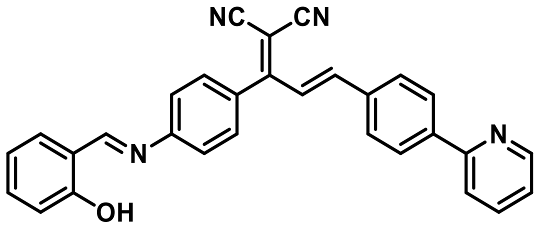

Dye **12**

Solvent	λ^{abs}_{max} (nm)	λ^{em}_{max} (nm)	*Stokes shift* (nm)	*Stokes shift* (cm^{-1})	Φ_F	ε ($mM^{-1}cm^{-1}$)
ACN	377	459	82	4783	n.d	52.7
DMSO	385	496	111	5796	n.d	50.5
DCM	381	473	92	5114	n.d	30.4
THF	380	458	78	4501	n.d	56.9
PhMe	384	578	194	8729	n.d	53.1

n.d, it couldn't be determined.

5. Interaction of dyes 8–12 with hydroxide anion

5.1. Interaction of dye 8 with hydroxide anion

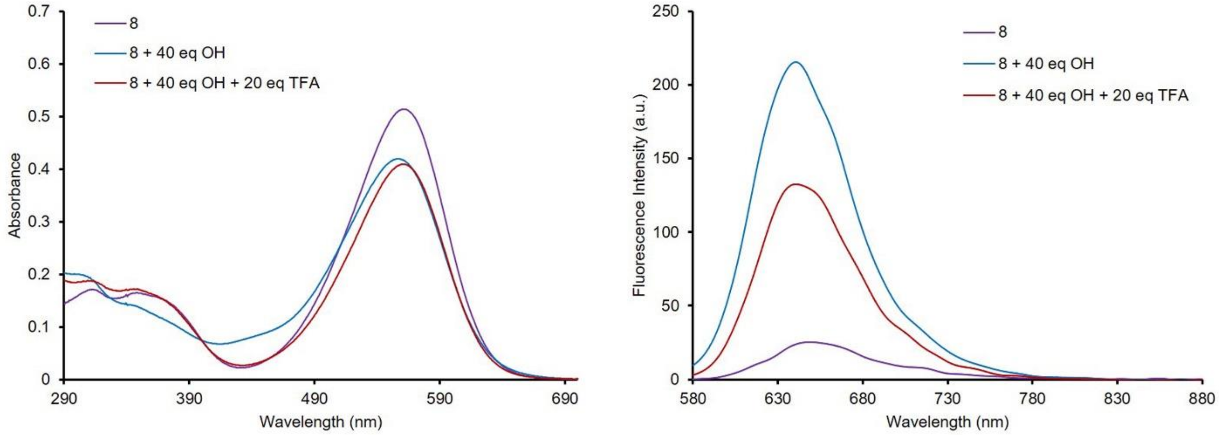

Figure S73: UV-Vis absorption (left) and Fluorescence (right) spectrum of dye **8** in DMSO while being treated with 40 equiv of Tetrabutylammonium hydroxide (TBAOH) and 20 equiv of Trifluoroacetic acid (TFA).

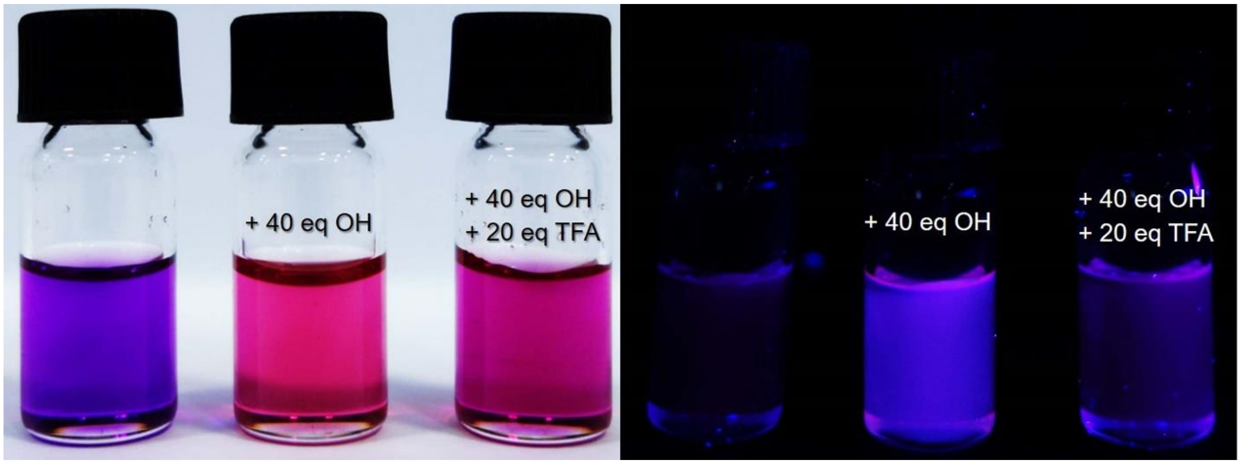

Figure S74: Photographs of dye **8** in DMSO under ambient light (left) and 365 nm UV lamp (right) while being treated with 40 equiv of Tetrabutylammonium hydroxide (TBAOH) and 20 equiv of Trifluoroacetic acid (TFA).

5.2. Interaction of dye 9 with hydroxide anion

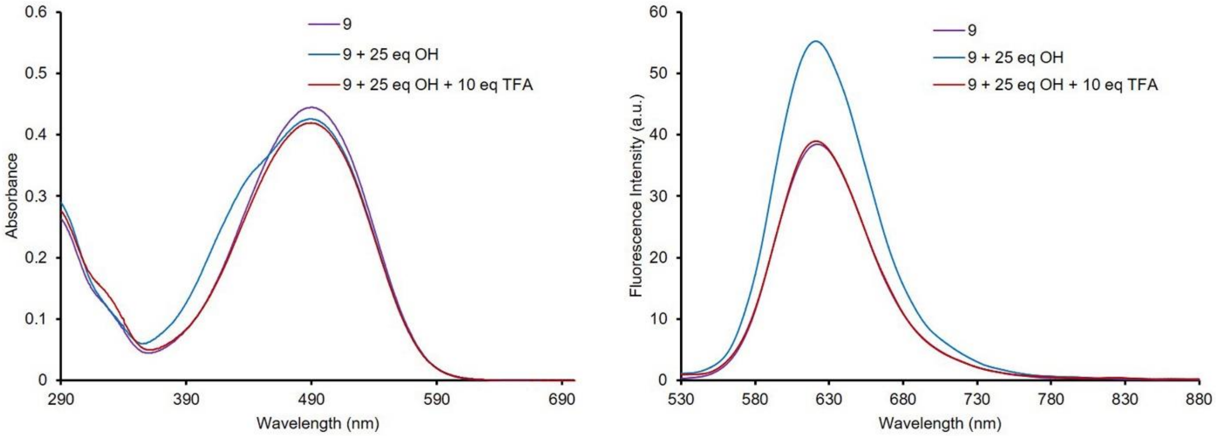

Figure S75: UV-Vis absorption (left) and Fluorescence (right) spectrum of dye **9** in DMSO while being treated with 25 equiv of Tetrabutylammonium hydroxide (TBAOH) and 10 equiv of Trifluoroacetic acid (TFA).

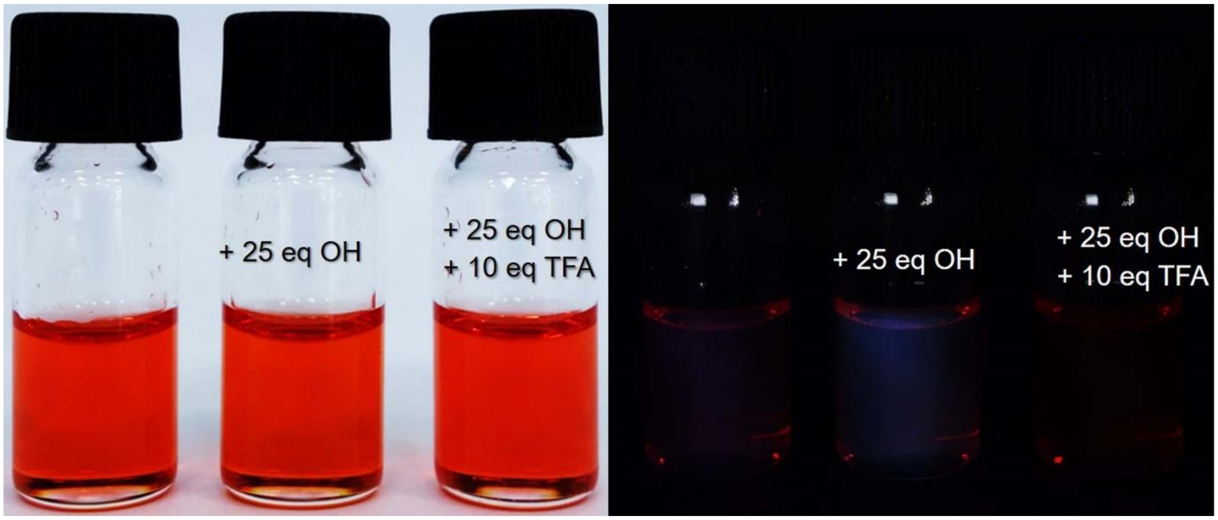

Figure S76: Photographs of dye **9** in DMSO under ambient light (left) and 365 nm UV lamp (right) while being treated with 25 equiv of Tetrabutylammonium hydroxide (TBAOH) and 10 equiv of Trifluoroacetic acid (TFA).

5.3. Interaction of dye 10 with hydroxide anion

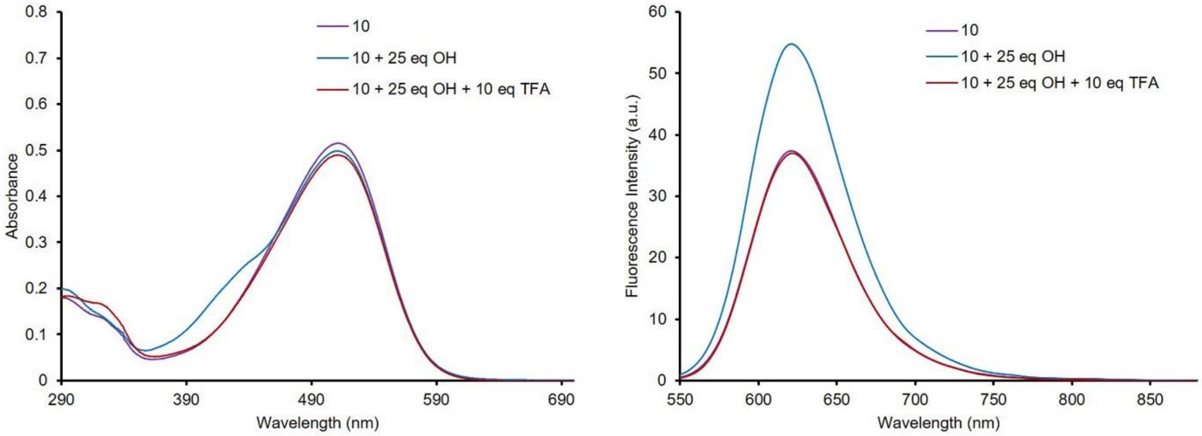

Figure S77: UV-Vis absorption (left) and Fluorescence (right) spectrum of dye **10** in DMSO while being treated with 25 equiv of Tetrabutylammonium hydroxide (TBAOH) and 10 equiv of Trifluoroacetic acid (TFA).

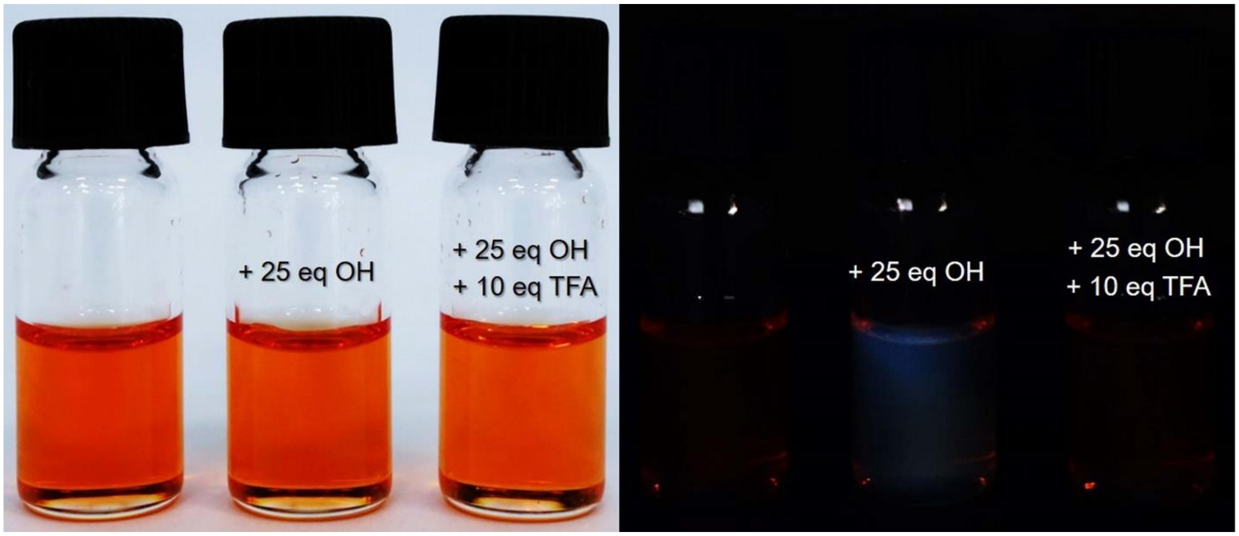

Figure S78: Photographs of dye **10** in DMSO under ambient light (left) and 365 nm UV lamp (right) while being treated with 25 equiv of Tetrabutylammonium hydroxide (TBAOH) and 10 equiv of Trifluoroacetic acid (TFA).

5.4. Interaction of dye 11 with hydroxide anion

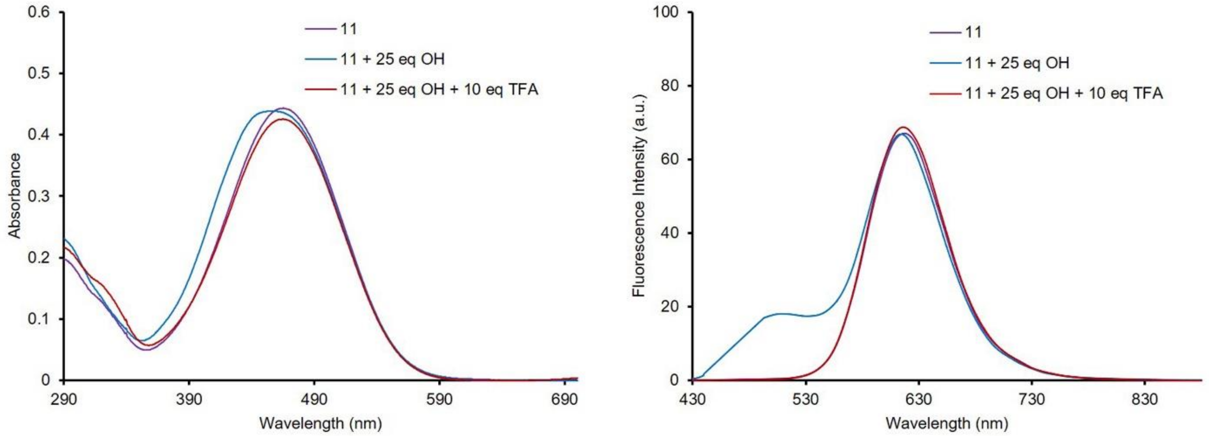

Figure S79: UV-Vis absorption (left) and Fluorescence (right) spectrum of dye **11** in DMSO while being treated with 25 equiv of Tetrabutylammonium hydroxide (TBAOH) and 10 equiv of Trifluoroacetic acid (TFA).

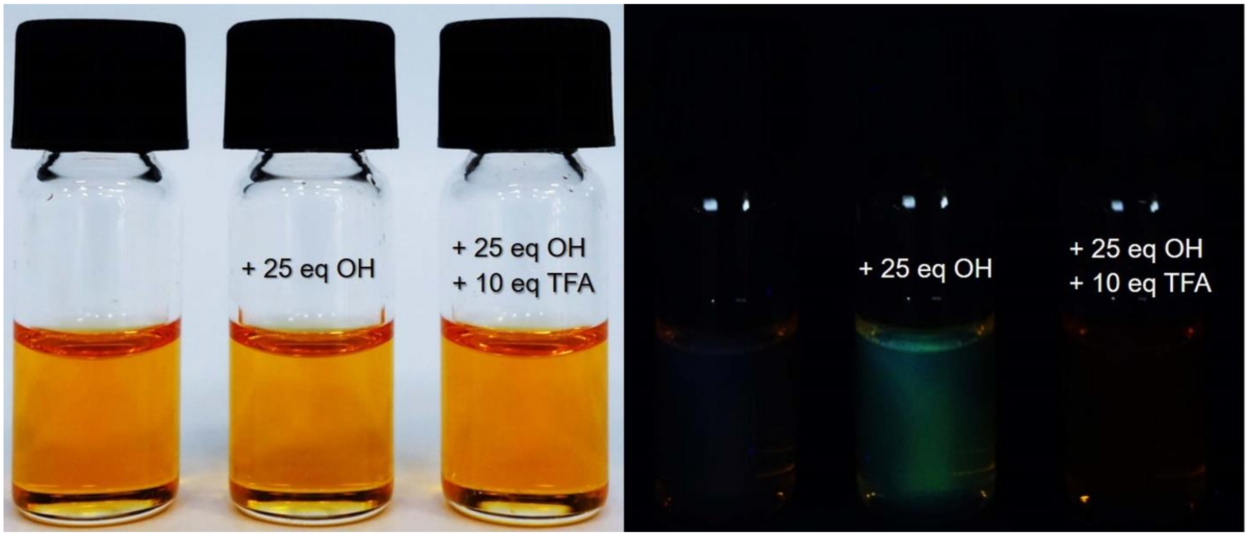

Figure S80: Photographs of dye **11** in DMSO under ambient light (left) and 365 nm UV lamp (right) while being treated with 25 equiv of Tetrabutylammonium hydroxide (TBAOH) and 10 equiv of Trifluoroacetic acid (TFA).

5.5. Interaction of dye 12 with hydroxide anion

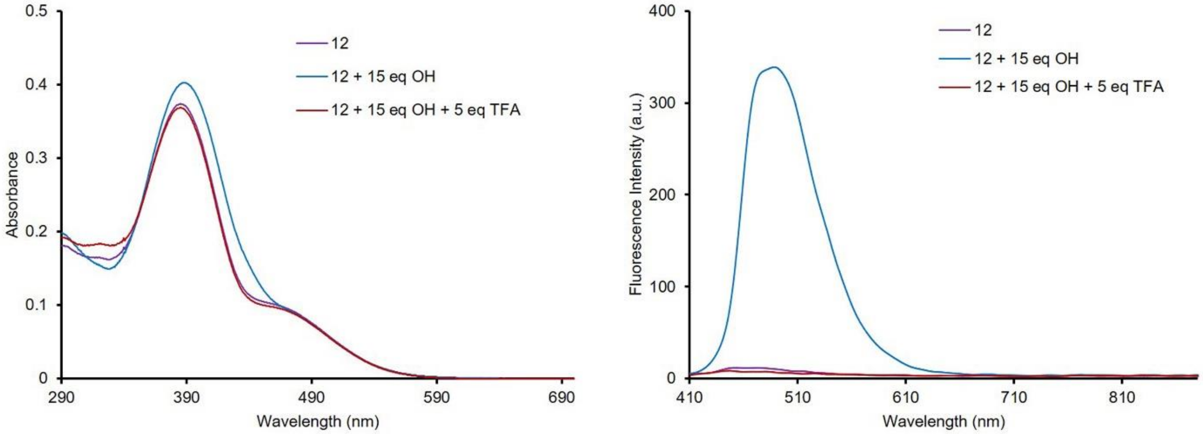

Figure S81: UV-Vis absorption (left) and Fluorescence (right) spectrum of dye **12** in DMSO while being treated with 15 equiv of Tetrabutylammonium hydroxide (TBAOH) and 5 equiv of Trifluoroacetic acid (TFA).

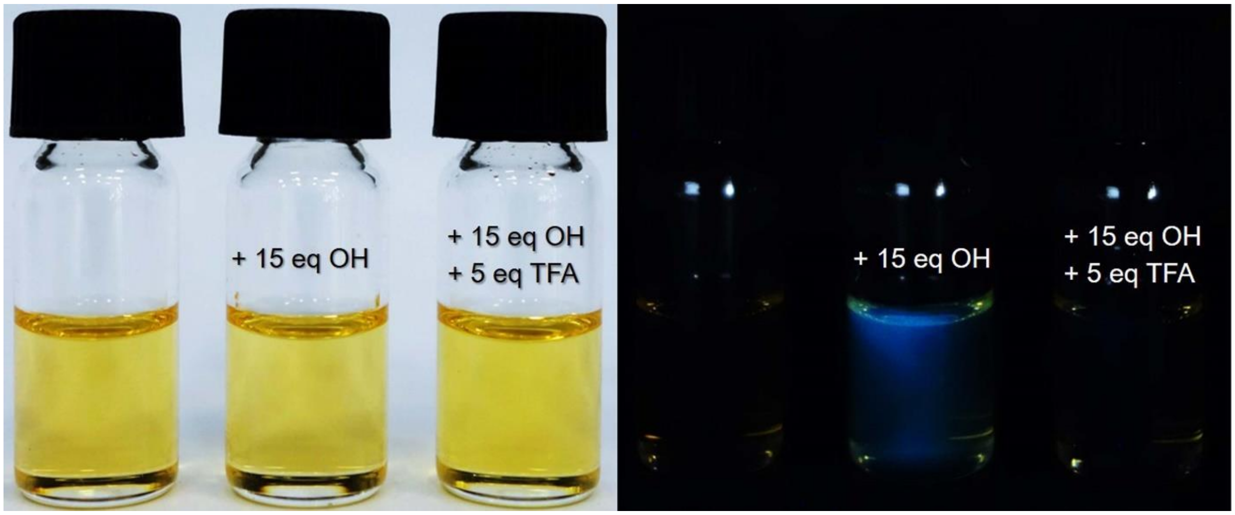

Figure S82: Photographs of dye **12** in DMSO under ambient light (left) and 365 nm UV lamp (right) while being treated with 15 equiv of Tetrabutylammonium hydroxide (TBAOH) and 5 equiv of Trifluoroacetic acid (TFA).

6. p*K*a calculation and pH properties of dye 8

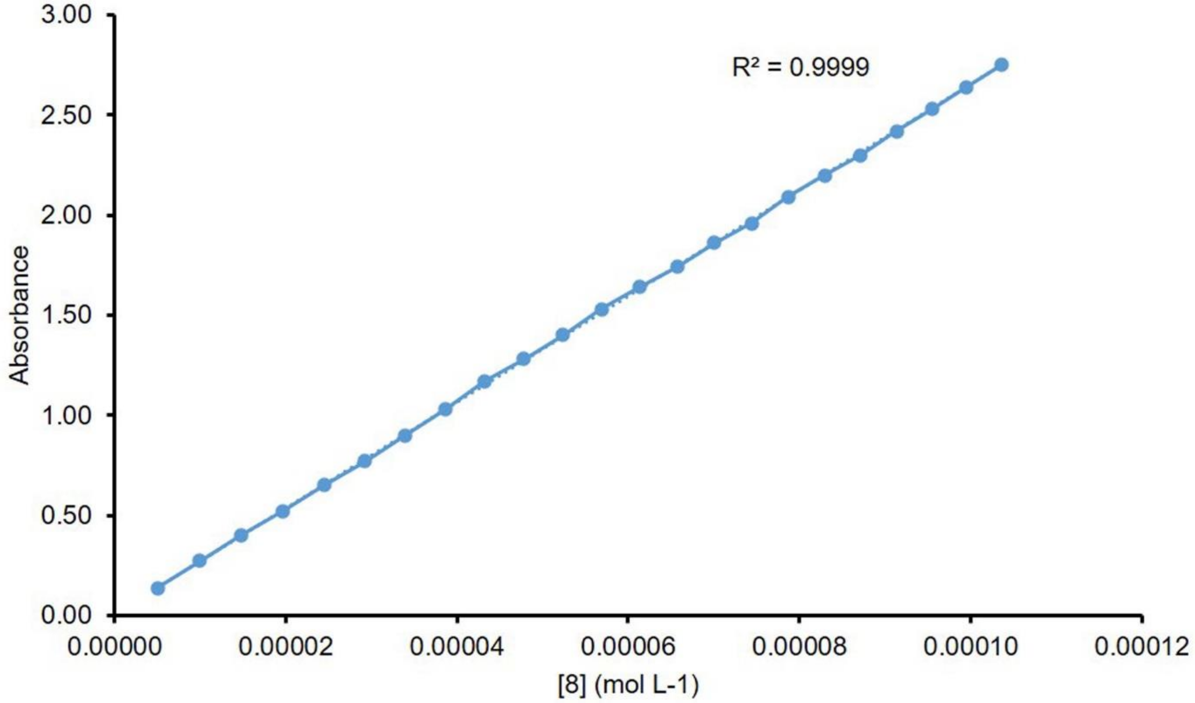

Figure S83: The calibration graph of dye **8** in water.

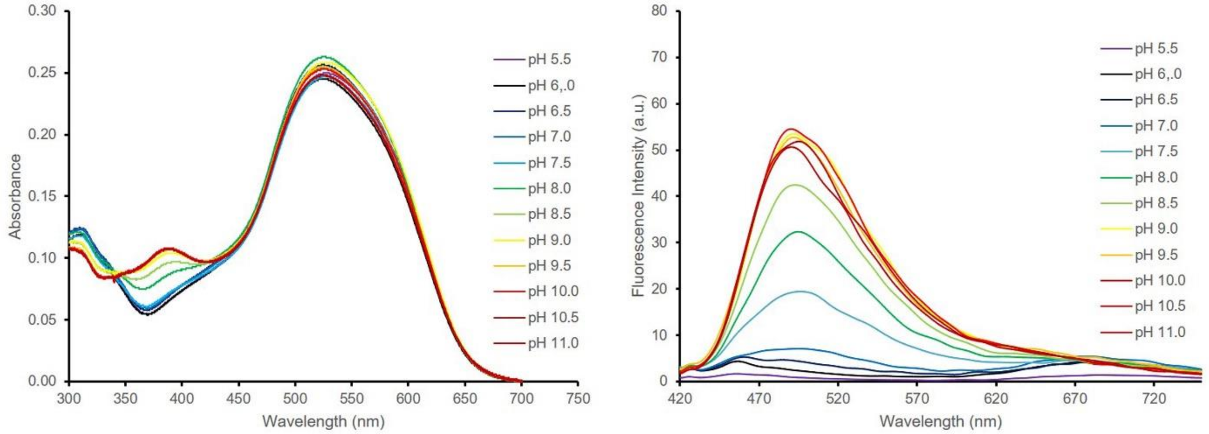

Figure S84: UV-Vis absorption (left) and Fluorescence (right) spectra of dye **8** in Britton-Robinson buffers with different pH values.

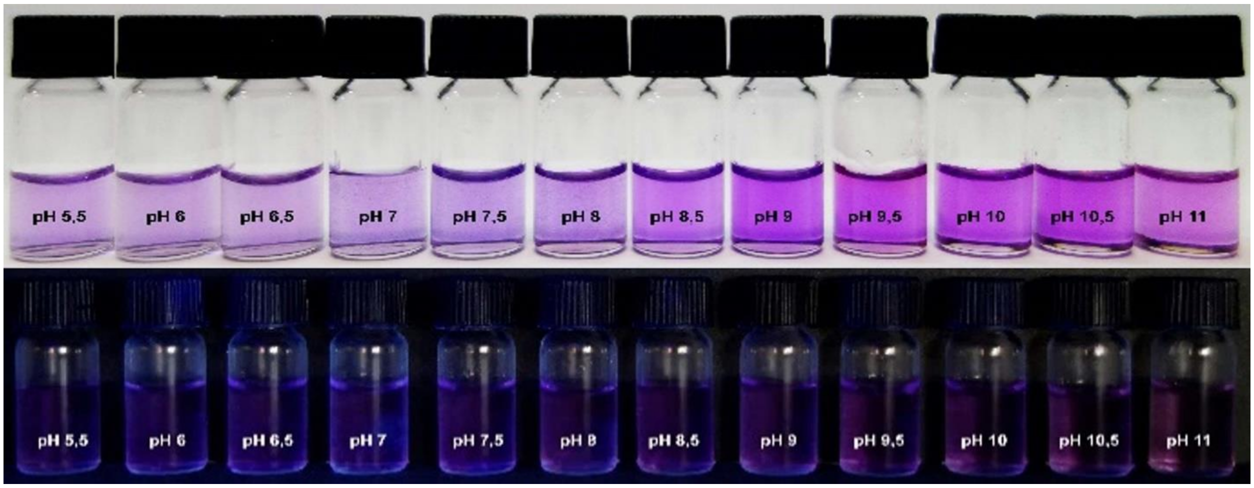

Figure S85: Photographs of dye **8** in Britton-Robinson buffers with different pH values .

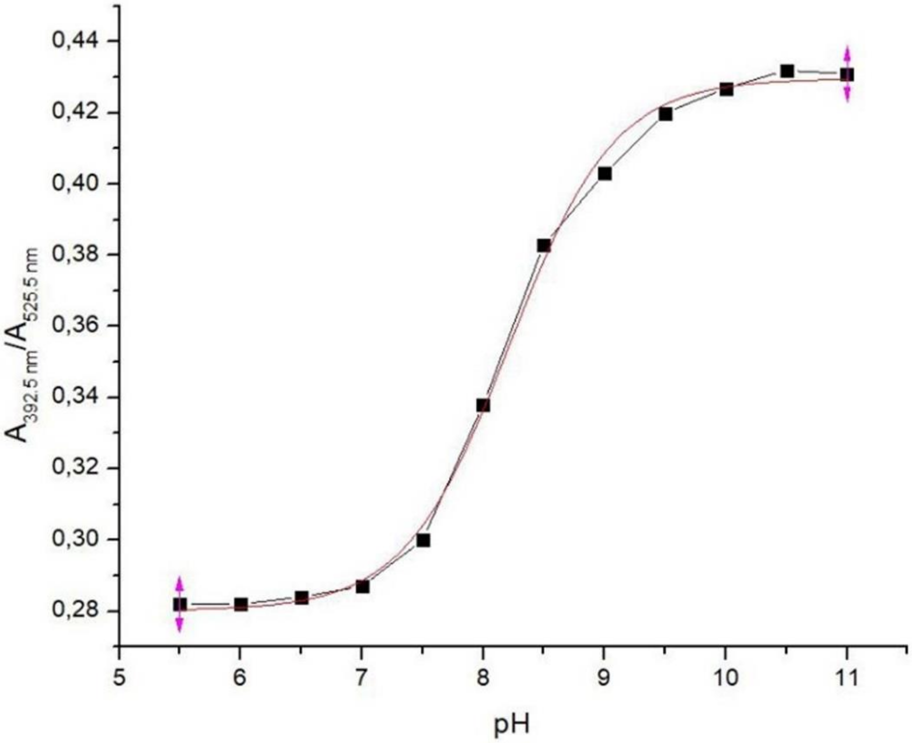

Figure S86: Sigmoid function obtained from dye **8** absorption spectra during pH investigation.

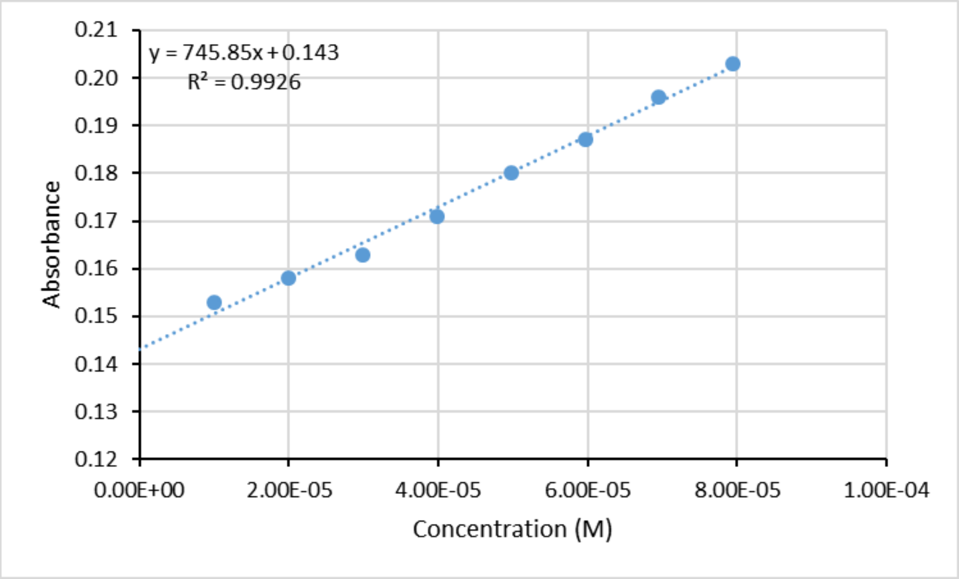

Figure S87: The absorbance calibration graph of OH^- for dye **8** in DMSO.

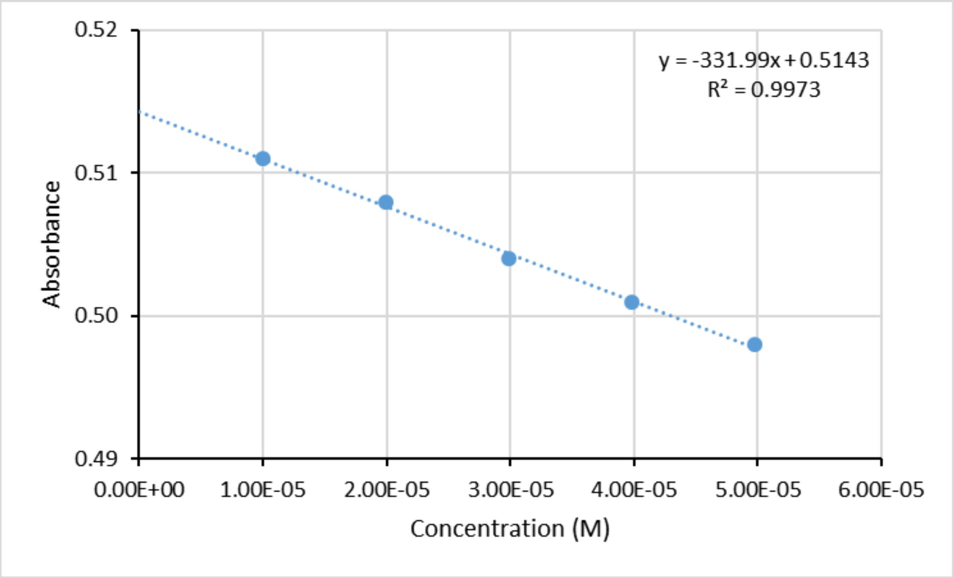

Figure S88: The absorbance calibration graph of OH^- for dye **9** in DMSO.

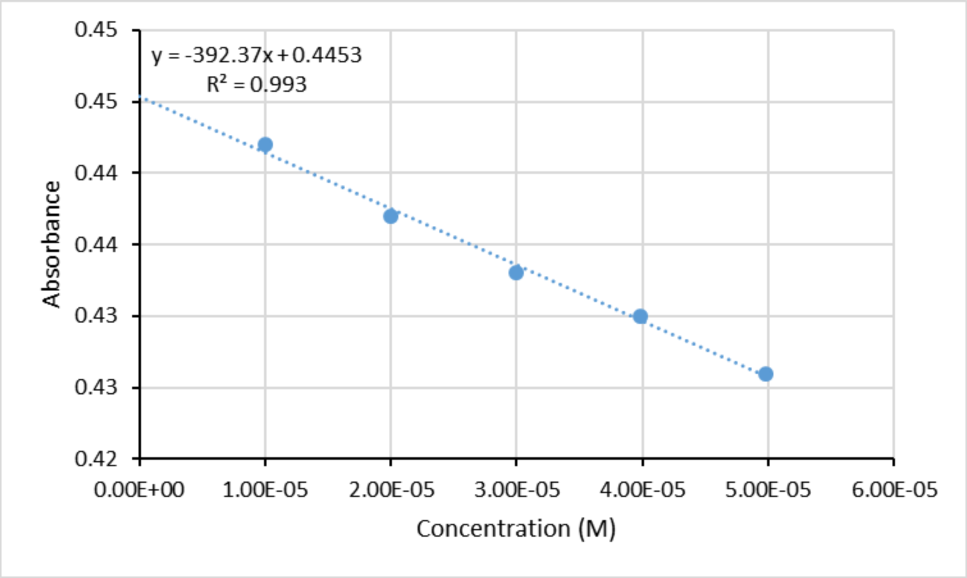

Figure S89: The absorbance calibration graph of OH^- for dye **10** in DMSO.

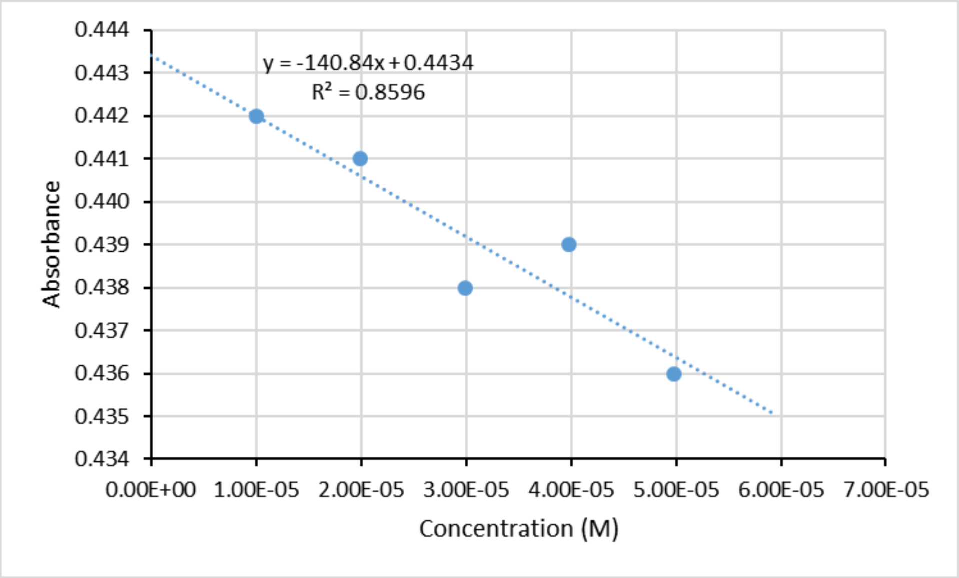

Figure S90: The absorbance calibration graph of OH^- for dye **11** in DMSO.

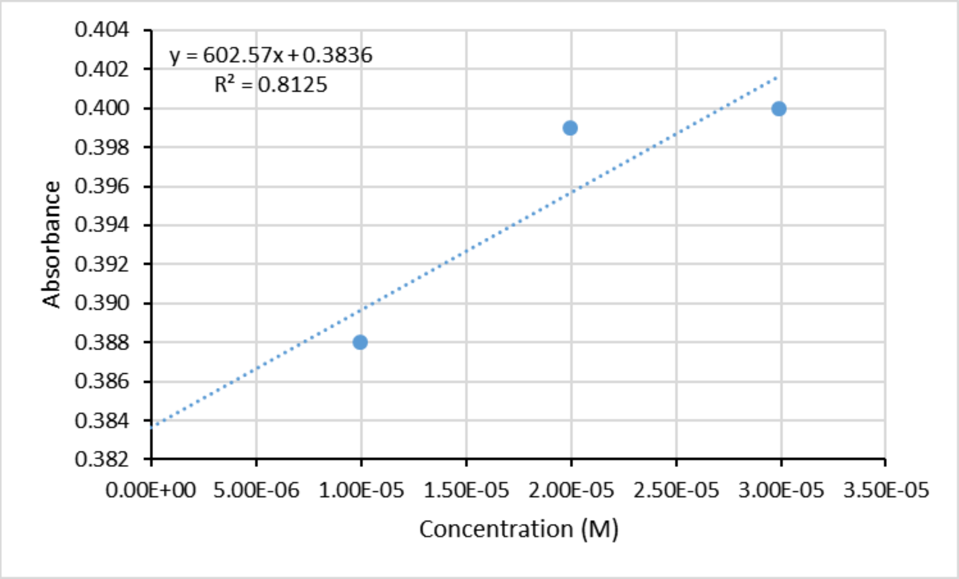

Figure S91: The absorbance calibration graph of OH^- for dye **12** in DMSO.

7. The Frontier Orbitals of dyes 3–12

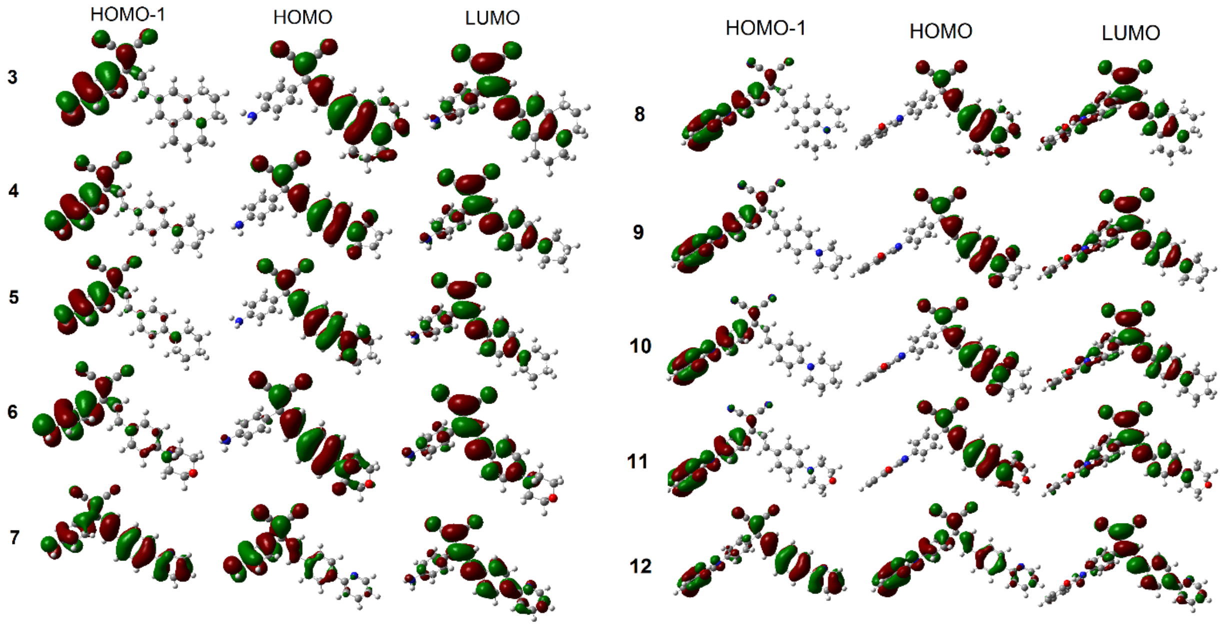

Figure S92: The frontier orbitals of dyes **3–7** and **8–12**.

8. The Calculated NLO Properties of dyes 3–12

Table S2. For dyes **3**-**7**, the electric dipole moment (*μ*) and the first hyperpolarizability (*β*) and their components calculated at B3LYP/6-31+G(d,p) level in $CHCl_3$ phase for all molecules. The component values are in a.u.

	**3**	**4**	**5**	**6**	**7**
μ_x	5.6926706	5.3691559	5.3873764	-4.0010258	-2.6429821
μ_y	-5.0934748	-4.9953649	-4.9429526	-4.8844605	-5.5436563
μ_z	-0.1588806	-0.1860062	-0.1252521	0.3016477	0.2053963
**μ (D)**	**19.42**	**18.65**	**18.59**	**16.07**	**15.62**
β_{xxx}	-42353.89934	-40753.23502	-43074.92636	39970.01067	18241.47831
β_{xxy}	9253.323169	8661.358561	8406.78102	8354.212588	5777.423659
β_{xyy}	4944.215718	5355.846317	5462.856917	-5410.250701	-5695.714921
β_{yyy}	2244.621108	2799.928022	2988.15785	3272.455142	4910.262606
β_{xxz}	-138.7166433	-59.3299487	-32.4921614	-91.5773624	-82.1969544
β_{xyz}	21.9511138	-28.6343988	-144.6699602	224.7418672	-43.7483347
β_{yyz}	-210.1739704	-188.6519652	-261.2191842	106.0693994	73.8419203
β_{xzz}	85.295379	92.2381221	38.5383168	-56.3878318	-44.8895418
β_{yzz}	-145.9765794	-162.1035225	-147.2250783	-168.0835493	-179.4030649
β_{zzz}	-9.918861	-16.035333	-14.1630701	26.276116	13.6302135
**β ($\times10^{-30}$) esu**	**337.1**	**320.3**	**338.9**	**314.1**	**141.1**

Table S3. For dyes **8–12**, the electric dipole moment (μ) and the first hyperpolarizability (β) and their components calculated at the B3LYP/6-31+G(d,p) level in $CHCl_3$ phase. The component values are in a.u.

	**8**	**9**	**10**	**11**	**12**
μ_x	3.7924911	3.7906052	3.4980186	3.3261378	1.2851961
μ_y	-6.5693216	-6.1665709	-5.7320051	-6.0617519	-5.1738506
μ_z	-1.0039384	-0.9597765	-0.7532799	0.2234769	-1.3860644
**μ (D)**	**19.45**	**18.56**	**17.18**	**17.59**	**14.00**
β_{xxx}	-18930.84765	-20156.66861	-23576.99201	-20567.67172	-7081.453568
β_{xxy}	21061.94446	21115.85202	22170.42169	20514.82425	14462.24417
β_{xyy}	-12080.54044	-10108.34981	-9362.886521	-8803.794064	-3542.006075
β_{yyy}	5957.890881	4533.609379	4304.142003	3686.920776	3017.223993
β_{xxz}	-638.8925296	-848.1828417	-1279.140573	938.5067506	-868.357574
β_{xyz}	-187.4450288	-177.4661063	98.8794399	-141.3188612	-323.454651
β_{yyz}	-339.3493952	-305.2196307	-361.542821	22.4412112	-249.4746136
β_{xzz}	-106.6930768	-79.2209604	71.6384865	-28.5927489	-124.9090923
β_{yzz}	-254.0791918	-276.116813	-377.5483294	-257.0611126	-253.9652693
β_{zzz}	-239.3627572	-230.7984379	-37.3557272	26.6287166	-245.8979212
**β(x10^{-30}) (esu)**	**354.8**	**341.9**	**362.9**	**327.7**	**175.8**

References

1. Reichardt, C. *Chem. Rev.* **1994**, *94* (8), 2319–2358. doi:10.1021/cr00032a005.
